# Mapping of ^1^H NMR chemical shifts relationship with chemical similarities for the acceleration of metabolic profiling: Application on blood products

**DOI:** 10.1002/mrc.5392

**Published:** 2023-09-04

**Authors:** Panteleimon G. Takis, Varvara A. Aggelidou, Caroline J. Sands, Alexandra Louka

**Affiliations:** ^1^ Section of Bioanalytical Chemistry, Division of Systems Medicine, Department of Metabolism, Digestion and Reproduction Imperial College London London UK; ^2^ National Phenome Centre, Department of Metabolism, Digestion and Reproduction Imperial College London London UK; ^3^ Department of Biological Applications and Technologies University of Ioannina Ioannina Greece; ^4^ Department of Clinical and Experimental Epilepsy, Queen Square Institute of Neurology University College London London UK

**Keywords:** ^1^H NMR, automation, biofluid, blood, metabolites identification, metabolomics

## Abstract

One‐dimensional (1D) proton‐nuclear magnetic resonance (^1^H‐NMR) spectroscopy is an established technique for the deconvolution of complex biological sample types via the identification/quantification of small molecules. It is highly reproducible and could be easily automated for small to large‐scale bioanalytical, epidemiological, and in general metabolomics studies. However, chemical shift variability is a serious issue that must still be solved in order to fully automate metabolite identification. Herein, we demonstrate a strategy to increase the confidence in assignments and effectively predict the chemical shifts of various NMR signals based upon the simplest form of statistical models (i.e., linear regression). To build these models, we were guided by chemical homology in serum/plasma metabolites classes (i.e., amino acids and carboxylic acids) and similarity between chemical groups such as methyl protons. Our models, built on 940 serum samples and validated in an independent cohort of 1,052 plasma‐EDTA spectra, were able to successfully predict the ^1^H NMR chemical shifts of 15 metabolites within ~1.5 linewidths (Δ*v*_1/2_) error range on average. This pilot study demonstrates the potential of developing an algorithm for the accurate assignment of ^1^H NMR chemical shifts based solely on chemically defined constraints.

## Graphical abstract

Mapping the chemical shifts of various metabolites' classes such as amino acids, fatty acids, alcohols, and sugars in a 1 K serum cohort led to the construction of linear models with high predicting accuracy of ^1^H NMR chemical shifts, minimal computational cost, and complexity, significantly expediting NMR metabolomics pipeline. The proposed strategy/algorithm, validated in an independent dataset of 1 K plasma samples, sets the grounds of a highly accurate chemical shifts' predictor for numerous metabolites via chemical similarities.

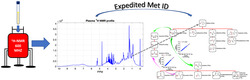

## 1 INTRODUCTION

Proton nuclear magnetic resonance (^1^H NMR) spectroscopy is one of the dominant analytical tools for complex mixture analysis and metabolic profiling of biofluids.^1^ The main assets of NMR are the minimal requirements for sample preparation; the intact biological matrix analysis; the direct quantification of metabolites via ^1^H NMR signals integration; and the ease of automation for high‐throughput analysis of large cohorts mainly owing to the high reproducibility of NMR data.^2^, ^3^, ^4^ Altogether, these advantages make NMR an ideal tool for metabolomics including clinical applications for diseases biomarkers research, health monitoring, and large population screening studies.

The effective application of NMR for diseases diagnosis requires the fast and thorough interpretation of the NMR data into metabolites concentrations. This can be achieved by (1) the accurate identification of signals from the maximum number of metabolites (i.e., potential biomarkers) signals and (2) their subsequent integration.^5^ It is widely known that quantification of metabolites can be achieved by NMR peaks deconvolution via known mathematical approaches^6^, ^7^; however, accurate and rapid automated identification of metabolites is still challenging.^8^, ^9^ Complex mixtures (e.g., biofluids) consist of up to several thousands of chemical compounds and macromolecules in variable concentrations,^10^ experiencing various chemical interactions, pH changes, and so forth that can influence the position of the resulting ^1^H NMR signals chemical shifts (*δ*).^11^ Several bioinformatic tools have been developed with the aim to semi‐automate or fully automate metabolite signal annotation and integration for both urine and blood serum/plasma biofluids and to deliver the relative and/or absolute concentrations of a panel of metabolites; these include BATMAN,^7^ Bayesil,^12^ AQuA,^13^ ASICS,^14^ and rDolphin.^15^ The majority of these software employs metabolites databases (e.g., HMDB^10^ and BMRB^16^) for the extraction of metabolites ^1^H NMR fingerprints, using them as an input for pattern recognition in the biofluids NMR profiles. This “matching” procedure, often coupled with traditional NMR constraints such as *J*‐coupling constants, not only requires a lot of computational time and cost especially for over‐crowded spectral regions (e.g., methyl region) but also is frequently prone to mis‐assignments due to matrix and/or experimental conditions effects on the lineshape of NMR signals.^8^ The risk of mis‐assignments is further increased for any singlets, as the *J*‐coupling constraint does not apply. To mitigate these problems, various solutions have been proposed so far, such as the acquisition of multi‐nuclear, multi‐dimensional NMR spectra alongside the routine one‐dimensional (1D) spectra,^17^ machine learning approaches, and the acquisition of multiplatform experiments. Although these approaches can minimize the risk of mis‐assignments, they require either high experimental cost or the construction of extra databases and often more complex algorithms/cheminformatics tools, which are not easily operated and reproduced by non‐experts.

Recently, we have shown that *δ* from NMR signals of various serum/plasma metabolites can be correlated without using chemistry‐related rules, by minimizing the spectral search window (<0.0060 ppm) for their automated identification,^18^ provided that NMR profiles are first calibrated to the glucose anomeric proton signal. Building upon these observations and the concept of employing chemical similarities to map relationships between chemically homologous protons δ (a method utilized in natural products research^19^, ^20^), we tried to construct the simplest models that could predict *δ* from various metabolites' signals. To achieve this, we assumed that protons of similar chemical groups from the same class of metabolites (i.e., amino‐acids and carboxylic acids) experience analogous matrix effects; thus, they should be highly correlated. Models were constructed on 940 serum samples ^1^H NMR profiles, and their predicting accuracy was successfully tested on an independent cohort of 1,052 plasma samples. Based upon our results, we proposed potential strategies for the efficient and rapid assignment of 16 serum/plasma metabolites via the combination of the constructed *δ* models (i.e., building maps of *δ* models), setting the grounds of a prospective, fully automated computational pipeline for metabolites identification, relying solely on chemistry and matrix related homologies and effects, respectively.

## 2 EXPERIMENTAL

### 2.1 Serum/plasma NMR samples preparation

All reagents used for spiking experiments and NMR sample preparation (e.g., for buffer composition) were purchased from Sigma‐Aldrich. Serum and EDTA‐plasma NMR samples for both employed studies were prepared under common standard operating procedures (SOPs).^2^, ^21^ In detail, NMR samples consisted of 50% plasma/serum buffer (75 mM Na_2_HPO_4_; 6.2 mM NaN_3_; 4.6 mM sodium trimethylsilyl [2,2,3,3‐d4]propionate [TMSP] in H_2_O with 20% [v/v] ^2^H_2_O; pH 7.4) and 50% of blood serum/plasma.

### 2.2 NMR spectra acquisition/processing

Serum NMR spectra (*n* = 940) were downloaded from the Metabolights repository (https://www.ebi.ac.uk/metabolights) (MTBLS395),^22^ and the plasma‐EDTA ^1^H NMR spectra (*n* = 1,052) were collected from internal databases (see Takis et al.^18^, ^23^). For both multicenter/independently collected cohorts, solution ^1^H NMR spectra were acquired using a Bruker 600 MHz spectrometer (Bruker BioSpin). Serum spectra were acquired by an NMR spectrometer equipped with a 5 mm CPTCI 1H‐13C‐31P and 2H‐decoupling cryoprobe including a *z*‐axis gradient coil, an automatic tuning‐matching (ATM), and an automatic sample changer and plasma spectra by an instrument equipped with a 5 mm BBI probe with 2H decoupling probe including a *z*‐axis gradient coil, an automatic tuning‐matching (ATM), automated shimming by Bruker TopShim along *Z* and *XY* plane, and an automatic refrigerated sample handling robot (Sample‐Jet). Temperature was regulated to 310 ± 0.1 K for both studies.

Two types of 1D ^1^H‐NMR experiments were acquired for each serum/plasma sample consisted of the standard 1D nuclear Overhauser effect spectroscopy pulse sequence NOESY (noesygppr1d; Bruker Biospin) and the standard spin echo Carr‐Purcell‐Meiboom‐Gill (CPMG) (cpmgpr1d; Bruker BioSpin) pulse sequence. NMR experimental details of serum and plasma spectra acquisition/processing are described in detail in Vignoli et al.^22^ and Dona et al.,^2^ respectively. In addition to the experiments, SMolESY^23^ profiles were also produced for increasing resolution and facilitating the assignment of any partially overlapped signals, while suppressing the macromolecular background.

### 2.3 Computational details

The recording of *δ* values (up to the 4th decimal of ppm) was achieved by via MATLAB function “findpeaks.m” (https://www.mathworks.com/help/signal/ref/findpeaks.html) and Topspin 4.0, after importing NMR spectra by getNMRdata.m function (https://github.com/pantakis/SMolESY_platform/blob/master/internal_functions/getNMRdata.m). Modern NMR‐based metabolomics hardware (e.g., Bruker IVDr^24^) provide high quality spectra (e.g., >65 k datapoints resolution, with spectral width ~20 ppm for the 600 MHz), allowing to record NMR peaks maxima within high accuracy (less than the third decimal of ppm). This is crucial not only for facilitating the identification of closely resonating signals in overcrowded spectral regions but also for allowing to minimize the error ranges of *δ* models predictions.^9^ The construction of *δ* models and the statistical analyses (e.g., calculation of root mean squared error [RMSE], relative RMSE [rRMSE], and goodness of fit [R^2^]) were performed in MATLAB (Mathworks, version 2021b) programming environment, with fitlm.m linear function (https://uk.mathworks.com/help/stats/fitlm.html) and homemade scripts. Furthermore, part of the statistical analyses and plotting was performed by Prism 9.4.1 (GraphPad Software, Inc, 2022).

## 3 RESULTS AND DISCUSSION

### 3.1 Chemical shifts matrices and models construction

**FIGURE 1:**
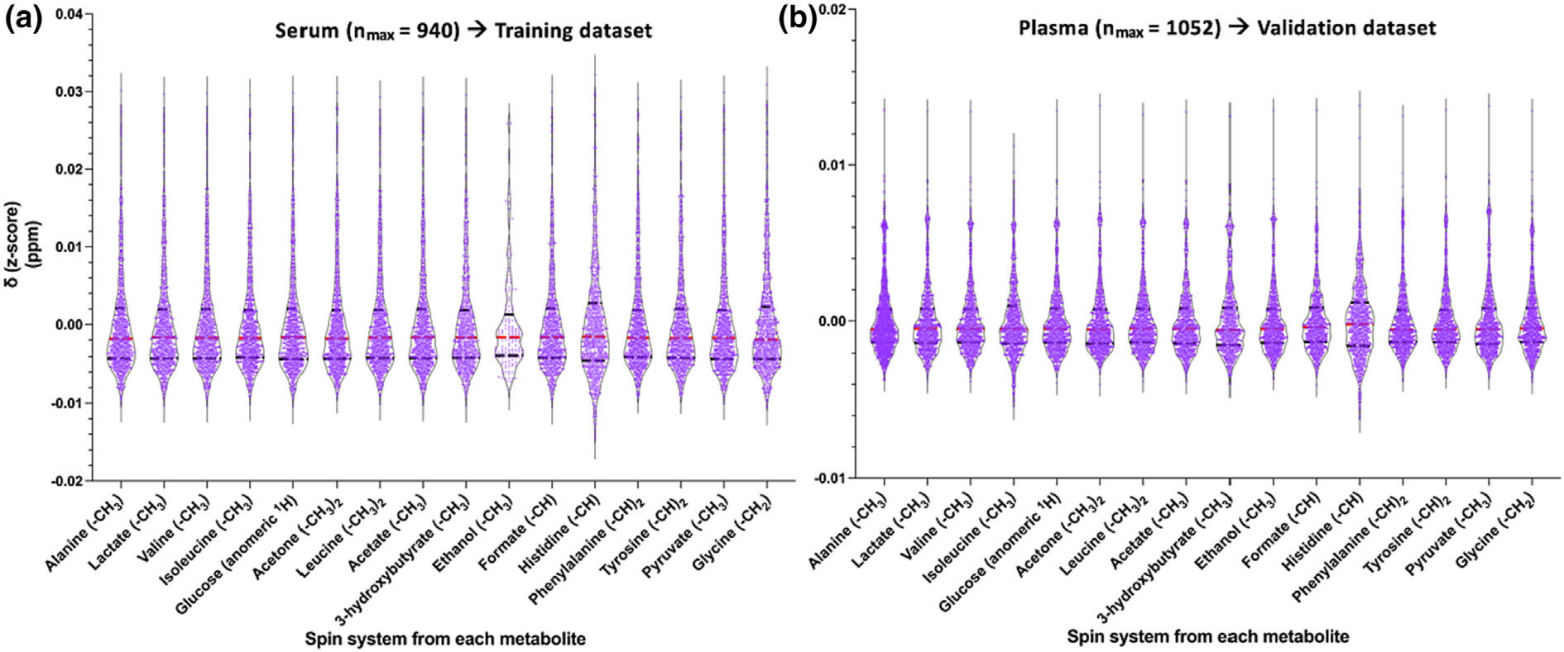
Sixteen spin systems' chemical shifts (*δ*) distribution plots for the (a) serum and (b) plasma‐EDTA datasets. For the data homogeneity, the mean value was subtracted by each spin system *δ* (i.e., *z*‐scored data).

Initially, we tried to assign at least one signal of the ^1^H NMR spin systems from common serum/plasma metabolites. For our pilot study, the selection of the metabolites was random; however, we tried to record ^1^H NMR signals *δ* values from a range of chemical groups and different classes of compounds. Sixteen ^1^H NMR spin systems *δ* from 16 metabolites (Table S1) were recorded (up to the 4th decimal of ppm) in almost 2,000 NMR spectra (940 serum and 1,052 plasma). For the multiplets, the average ppm value of all components was recorded. In case of any partial overlap, the average ppm value of multiplets can be calculated by taking into consideration the *J*‐coupling constants and the visible component(s) *δ* of the multiplet or taking advantage of the 2D *J*‐res spectra projections.^25^ The assignment of the selected spin systems resonating as multiplets was achieved mainly by the use of 2D *J*‐res spectra (only for the validation dataset) and statistical correlation spectroscopy (STOCSY)^26^ (see examples in Figure S1). As previously shown,^18^ for the metabolites exhibiting only one singlet, the assignment was validated (when needed) via spiking experiments (Figure S1) along with already published tools (i.e., SMolESY‐select,^18^ Chenomx NMR suite 8.1 [evaluation license] [www.chenomx.com]). All assignments were performed by an experienced NMR spectroscopist^27^ corroborated by the above software when needed (several examples of spiking experiments as well as assignments are described in Figure S1). Figure 1a,b depicts the distribution and *z*‐scored ranges of each metabolite *δ* for both serum and plasma matrices. It is immediately apparent that for both serum and plasma metabolites the chemical shift values are highly variable, commonly up to ~0.05 ppm range (i.e., ~50 linewidths [Δ*v*_1/2_] for the 600 MHz instrument) owing to different matrix effects such as changes in pH and variable metabolites' composition.^8^, ^9^ The maximum variability was observed in the serum cohort, hinting to its selection as training dataset, whereas plasma cohort was employed for the validation of models' predicting ability.

**FIGURE 2:**
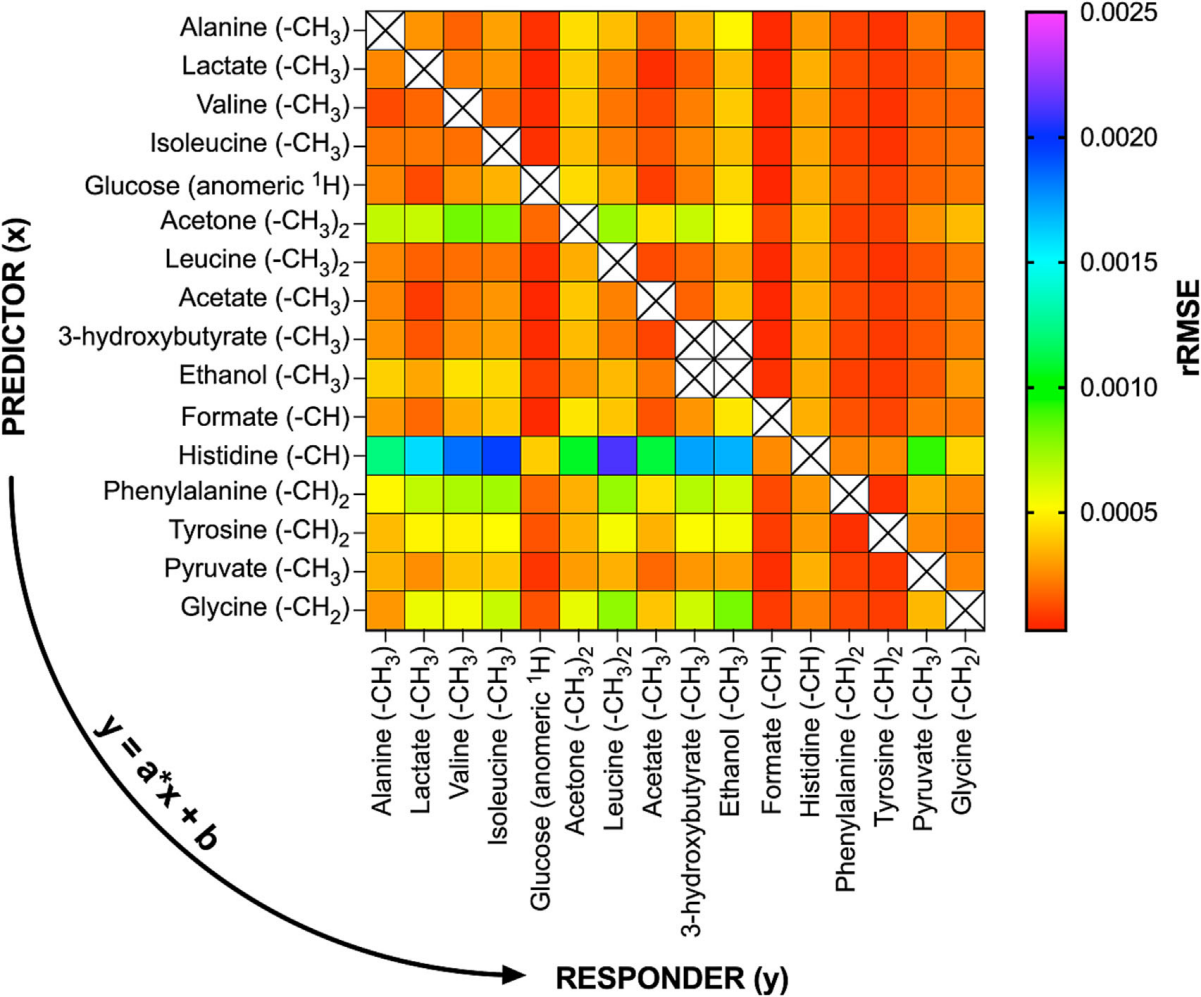
A heatmap summarizing the calculated relative root‐mean‐square error (rRMSE) values for each constructed linear regression model (*n* = 240) with all combinations of response (y) and predictor (x) spin systems *δ*. Between ethanol and 3‐hydroxybutyrate metabolites, regression models were statistically unstable due to the low number of assigned δ in serum spectra for both metabolites.

**FIGURE 3:**
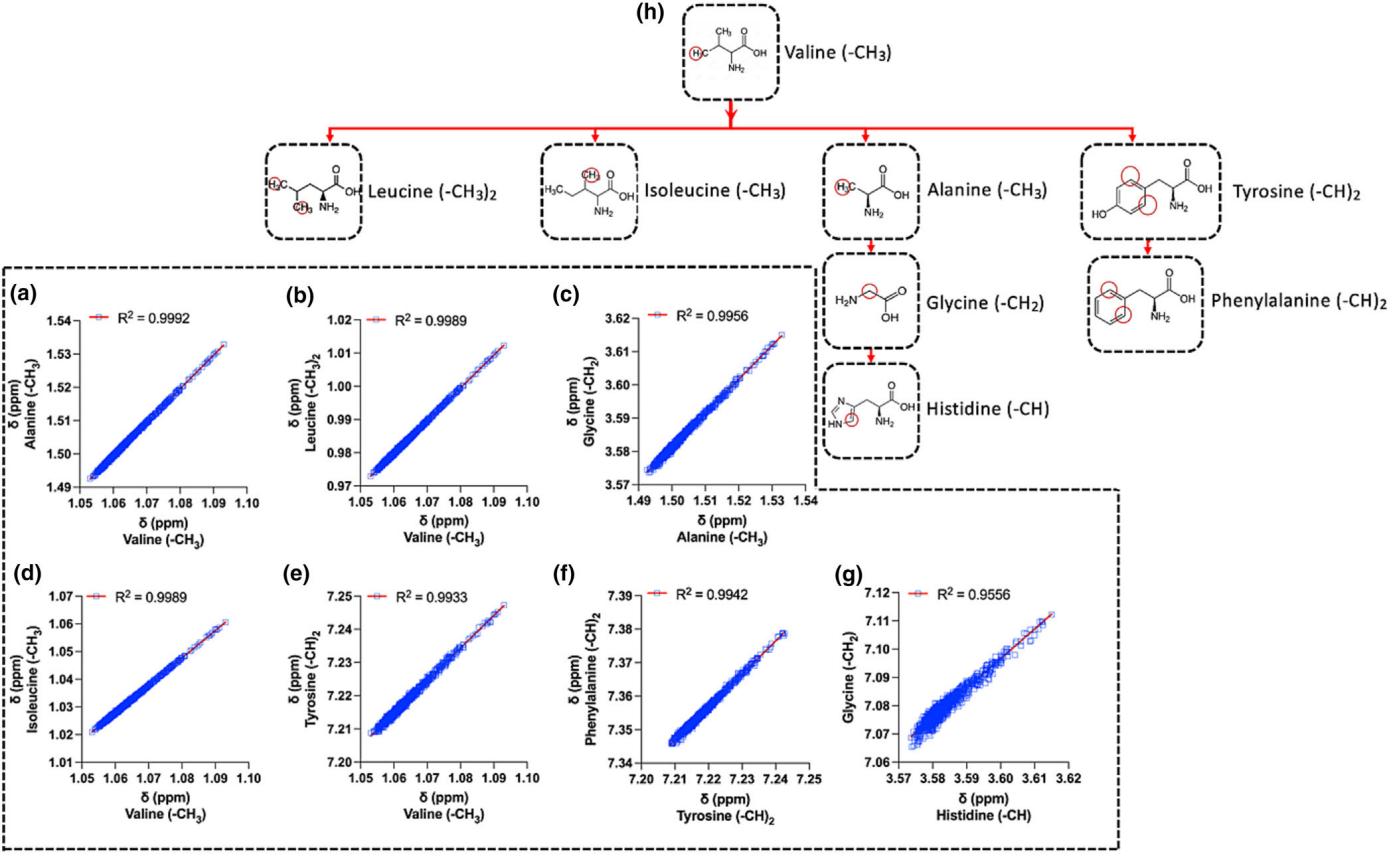
Linear regression models performance between valine's methyl protons δ and (a) alanine (‐CH_3_), (b) leucine (‐CH_3_)_2_, (c) glycine (‐CH_2_), (d) isoleucine (‐CH_3_), and (e) tyrosine (‐CH)_2_ as well as between (f) tyrosine (‐CH)_2_ and phenylalanine (‐CH)_2_ and (g) glycine (‐CH_2_) and histidine (‐CH). The combination of these models results in a (H) “map” of linear regressions, which requires only valine for the δ prediction of the remaining seven metabolites (i.e., amino acids).

The *δ* values of all assigned spin systems from the 16 serum metabolites were used to fit 240 linear regression models of the form: y = a*x + b, where y is the response and the x the predictor variable, respectively. After thorough inspection of the linear regression statistics for each fitted model (Figures S2–S17), it is clear that there is at least one fitted linear model for each spin system *δ* with R^2^ > 0.985 and high predicting ability (rRMSE < ±0.01%) with less than 1.5 linewidth error (<0.0015 ppm) (Figure 2). It should be noted that for histidine ‐CH model (*δ* range was up to ~50 Δ*v*_1/2_), the best predictor was glycine (R^2^ ~ 0.96, rRMSE ~ ±0.02%). Methyl protons from the aliphatic amino acids show the greatest degree of correlation among them and the corresponding linear regression models demonstrate the lowest rRMSE values (Figure 2). Linear regression models' performance indicates that valine's methyl proton *δ* could predict *δ* from alanine, leucine, isoleucine methyl protons, and tyrosine's ‐(CH)_2_ with less than one Δ*v*_1/2_ error range (i.e., <0.0010 ppm) (Figures 2 and 3a–e). Linear model from the aromatic ring protons of phenylalanine (responder) and tyrosine (predictor) shows the best association (Figures 2 and 3f), whereas interestingly, glycine's ‐CH_2_ protons correlate the most with the ‐CH from histidine's imidazole group (R^2^ ~ 0.96) and could predict the latter with the lowest error (rRMSE < ±0.03%) (Figure 3g). The combination of the best predictive models of the amino acids aliphatic protons results into a “map” of linear regression functions (Figure 3h), where the prior identification of valine's ‐CH_3_ doublet could lead to the predictions of six ^1^H NMR spin systems *δ* from six amino acids, respectively, within <1.5 Δ*v*_1/2_ accuracy and <3 Δ*v*_1/2_ for histidine. Therefore, following this map, the risk of mis‐assignments for the described spin systems is diminished, requiring infinitesimal computational time and cost.

**FIGURE 4:**
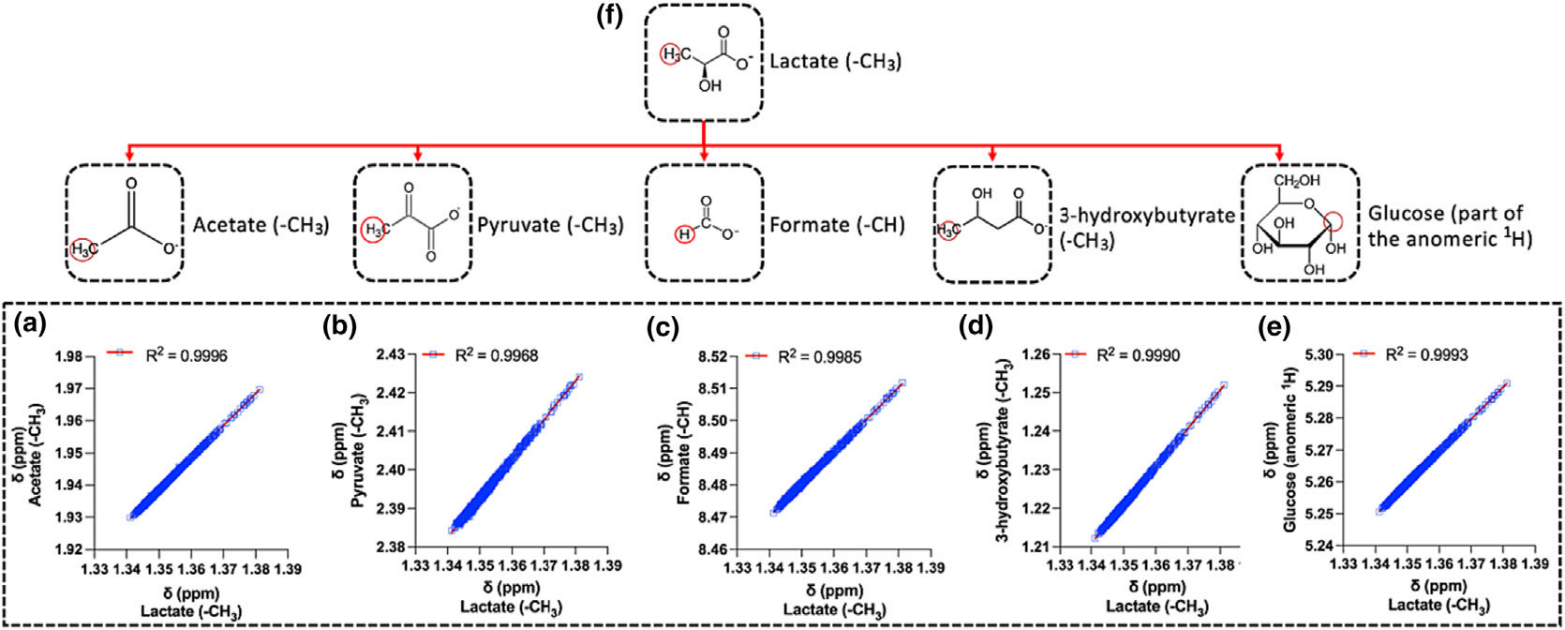
Linear regression models performance between lactate's methyl protons δ (as predictor, x) and (a) acetate (‐CH_3_), (b) pyruvate (‐CH_3_), (c) formate (‐CH_2_), (d) 3‐hydroxybutyrate (‐CH_3_), and (e) glucose (part of the anomeric proton). The combination of these models results into a (F) “map” of linear regressions, which requires only lactate for the δ prediction of the remaining five metabolites (i.e., carboxylic acids/sugar).

Another group of metabolites in our study consists of carboxylic acids, including lactate, acetate, formate, and 3‐hydroxybutyrate. The linear models (Figures 2 and 4a–d) among these metabolites' aliphatic protons showed the best performance (R^2^ > 0.997) and predicting accuracy (rRMSE < 0.02%, error range < 0.0014 ppm). Regression models showed that methyl group of lactate is the best predictor for the aliphatic protons *δ* of the other acids (Figure 4a–d). Lactate is an abundant (>0.2 mM), highly occurrent (>99%) serum/plasma metabolite, and its methyl protons' signal has a very characteristic pattern (i.e., a doublet; see Table S1) that facilitates its detection in the serum/plasma ^1^H NMR profiles.^10^ In contrast, 3‐hydroxybutyrate appears less frequently above the limit of detection via routine NMR experiments, whereas its methyl protons signal resonates in a more crowed spectral region. Furthermore, pyruvate, acetate, and formate protons exhibit only one singlet, increasing the risk of mis‐assignments. Consequently, in this group, the lactate methyl protons are ideal predictors of the remaining carboxylic acids. Interestingly, lactate showed an excellent linear correlation (R^2^ ~ 1) with the glucose anomeric proton (Figure 4e), showing potential for further extension to predict the chemical shift of other sugars. These observations allowed the construction of another regression models “map” (Figure 4f), indicating that *δ* of lactate's methyl group could significantly increase confidence in the assignment of several carboxylic acids as well as glucose (<1.5 Δ*v*_1/2_ error range).

**FIGURE 5:**
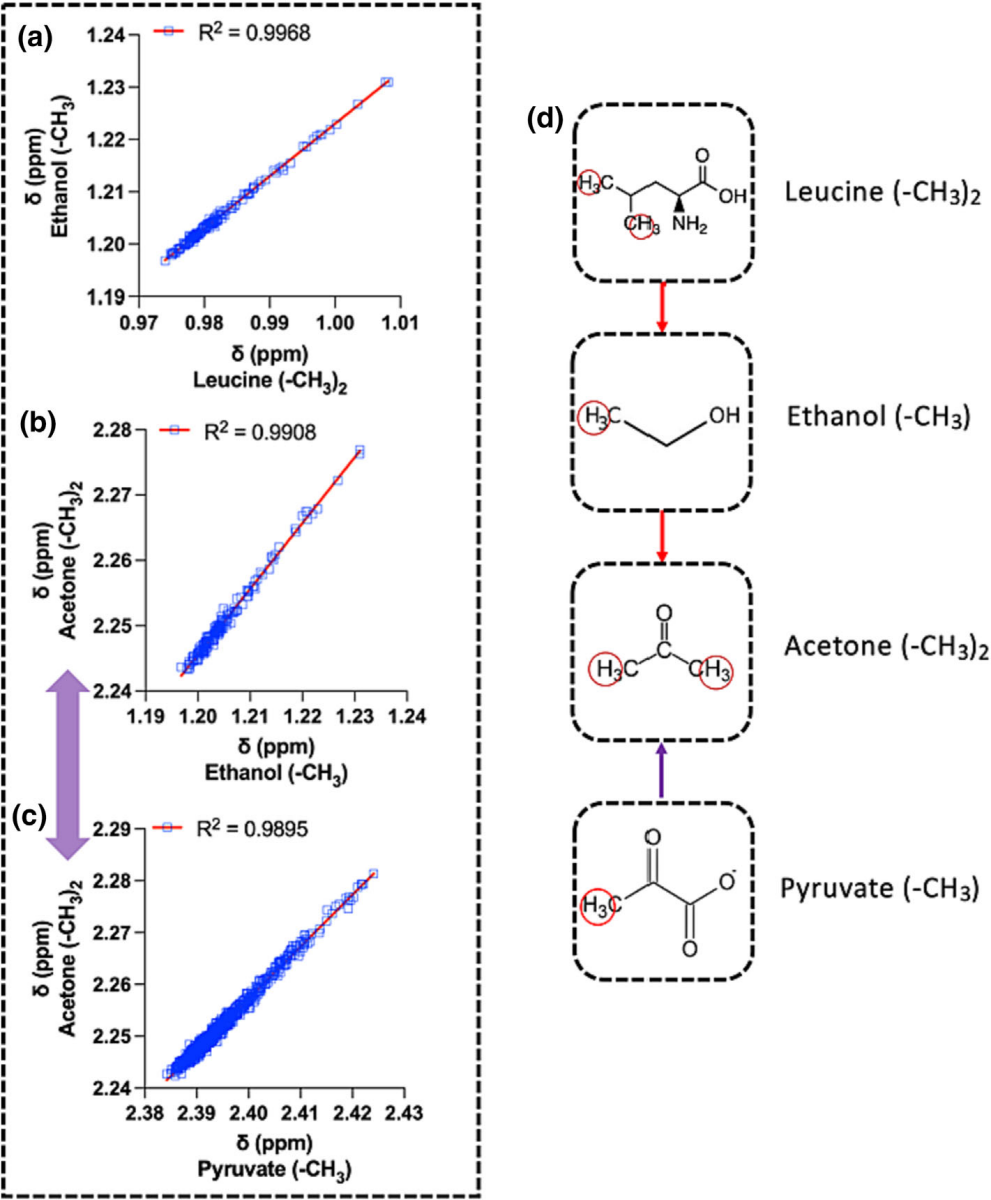
Linear regression models performance between (a) leucine's methyl protons δ (as predictor, x) and ethanol (‐CH_3_) and (b) ethanol's methyl protons δ (as predictor, x) and acetone (‐CH_3_)_2_. Since ethanol is mainly an exogenous metabolite, the linear model between (c) pyruvate (‐CH_3_) (as predictor, x) and acetone is depicted (purple double‐sided arrow), which is slightly outperformed by ethanol as acetone's predictor. The combination of these models results into a (d) “map” of linear regressions, which requires only leucine for the *δ* prediction of the remaining two metabolites (i.e., alcohols/ketones).

Ethanol is, on the whole, an exogenous metabolite (although it is endogenously produced in some pathological conditions^28^, ^29^) and was detected in 157 out of the 940 serum spectra. The *δ* of the ethanol methyl protons showed an near perfect linear correlation (R^2^ = 0.997) with the methyl protons of leucine (Figure 5a). Additionally, ethanol performed as an excellent predictor of acetone's methyl protons singlet within one Δ*v*_1/2_ (Figure 5b). Owing to the scarcity of ethanol's presence in serum/plasma NMR profiles, pyruvate methyl protons could be an alternative choice for predicting acetone within slightly lower accuracy (R^2^ = 0.99 and error range < 0.0020 ppm) (Figure 5c). Thus, leucine methyl protons could predict the *δ* values of ethanol and subsequently of acetone within one Δ*v*_1/2_ error range (i.e., within 0.0010 ppm), practically eliminating any mis‐assignments for these cases (Figure 5d).

**FIGURE 6:**
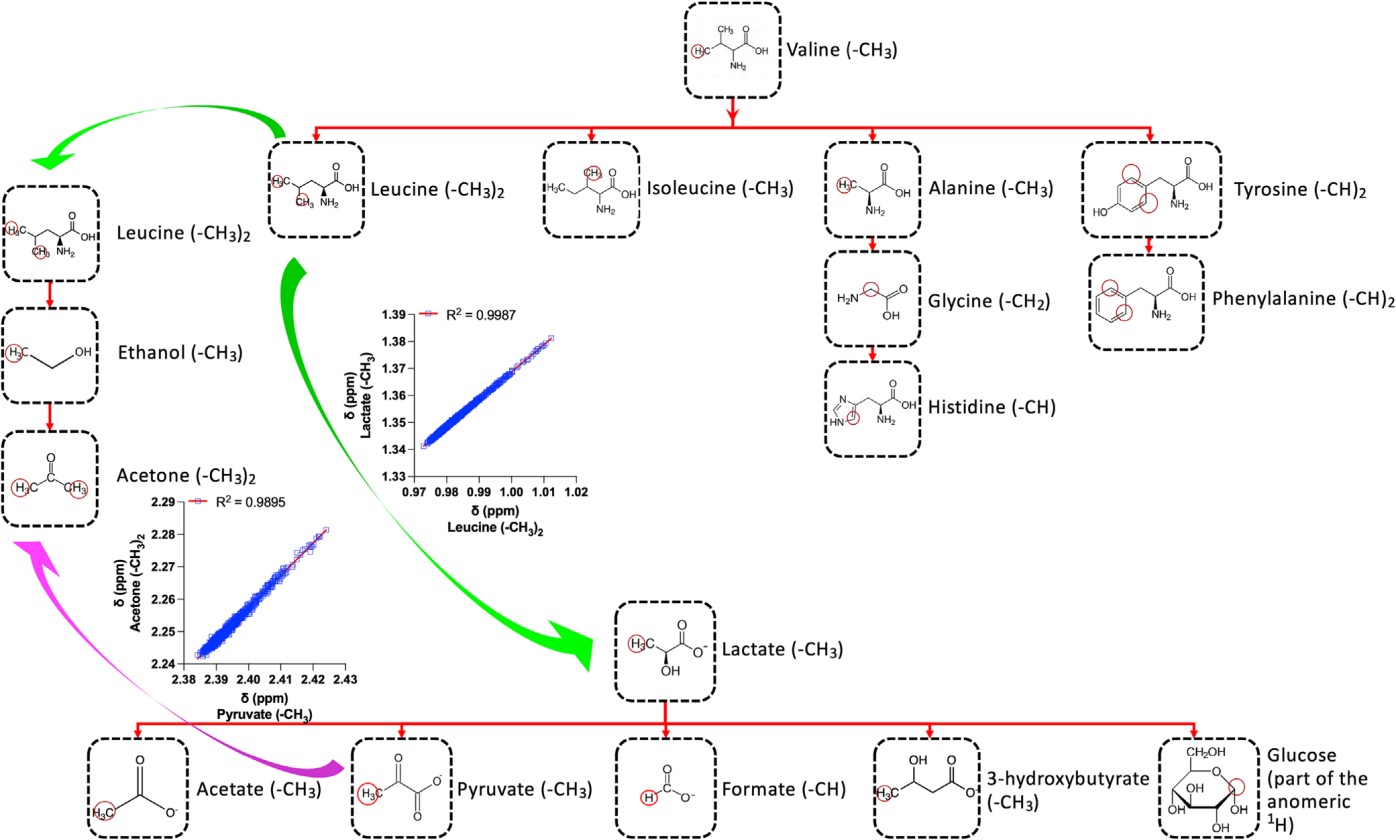
The scheme of the combined “sub‐maps” from various classes of the 15 serum/plasma metabolites, resulting into a final algorithm based upon the models with the highest predicting ability for each ^1^H NMR spin system δ. Green arrows along with the fitted linear regression lines plots point at the joints of connecting sub‐maps to the final algorithm, namely, at leucine methyl protons δ as predictors (x) of the lactate (R^2^ ~ 1) for carboxylic acids and ethanol for alcohols methyl protons δ. Purple arrow highlights the alternative way of predicting acetone and the linear regression plot of pyruvate as a predictor of acetone methyl protons δ is shown (R^2^ ~ 0.99).

To this point, the constructed linear regression “maps” could be adopted independently for the robust and reliable assignment of several metabolites, including amino‐acids, carboxylic acids, and alcohols/ketones. These three individual maps require only the *δ* values of the methyl protons from valine and lactate, since leucine can be predicted by valine (Figure 3b). Extending this further, the *δ* model between leucine (predictor) and lactate (responder) revealed that the latter could be directly predicted by leucine within less than 0.0010 ppm error (<1 Δ*v*_1/2_). As such, and as depicted in Figure 6, this allows the combination of all regression “maps” into one. The final “map” includes the most accurate *δ* models based upon the training dataset and predicts the *δ* of 15 ^1^H NMR signals from 15 metabolites with infinitesimal error and requiring only the initial position of valine's methyl group *δ*. It should be noted that all estimated errors per prediction step are based upon the prior assignment of each signal as each model's input. Nevertheless, results show that there is at least one key chemical group of at least one metabolite that “unlocks” the NMR signals positions from chemically homologous protons from a class of metabolites. It is also noted that mapping these underlying relationships between spin systems *δ* not only diminished the risk of misidentification but also significantly expedited signals assignment.

### 3.2 Models validation on independent datasets

**FIGURE 7:**
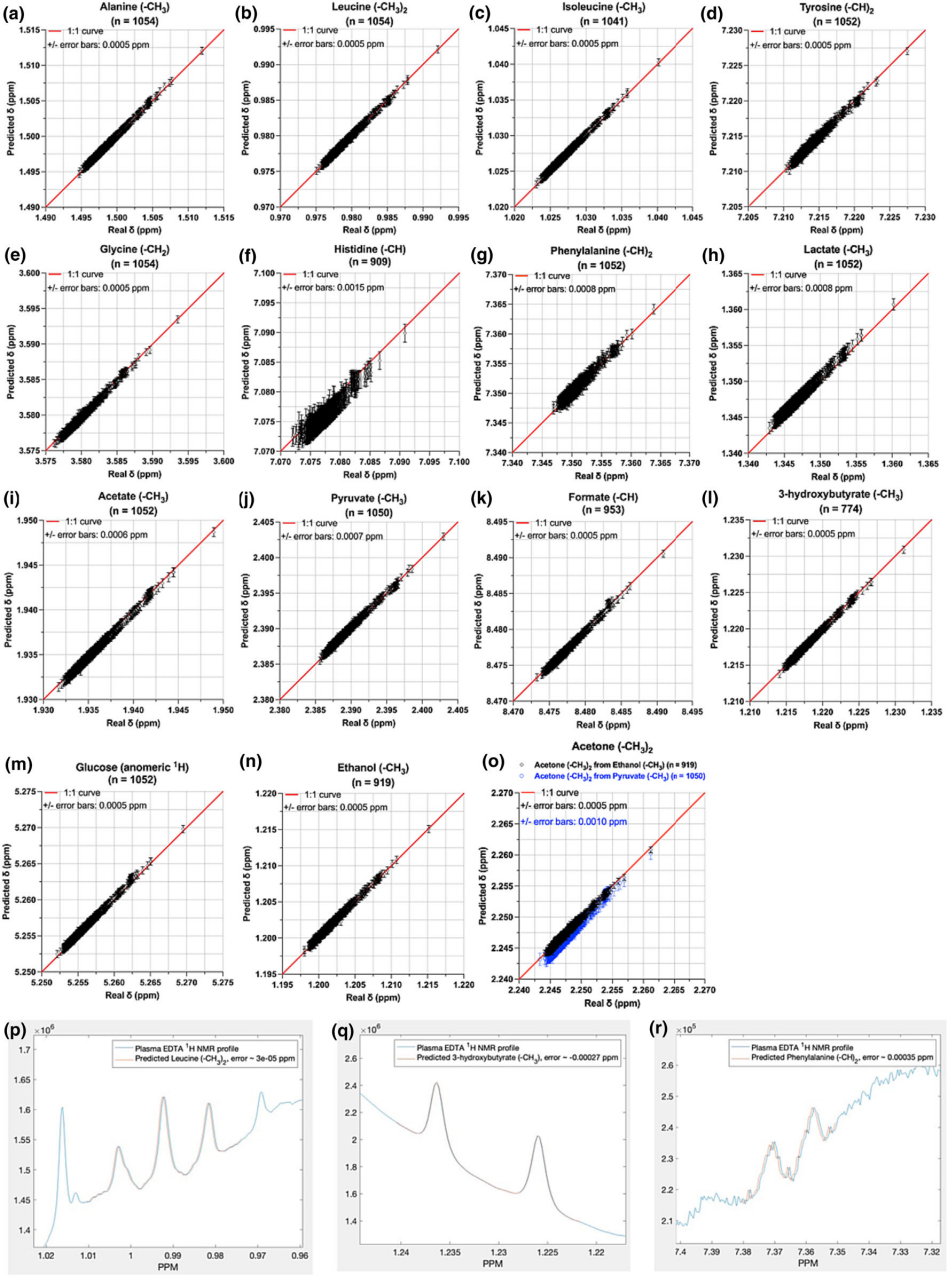
The semi‐automated *δ* prediction results from the plasma‐EDTA (validation) dataset, based upon the linear regression models that are described in Figure 6 versus the observed δ values (i.e., assigned *δ*) for each spin system. The red line represents the 1:1 curve (i.e., perfect line). Error bars correspond to the maximum ± error observed in each case. The plots of observed versus the predicted *δ* correspond to the spin systems: (a) alanine ‐CH_3_ doublet, (b) leucine (‐CH_3_)_2_ triplet, (c) isoleucine ‐CH_3_ doublet, (d) tyrosine (‐CH)_2_ multiplet, (e) glycine ‐CH_2_ singlet, (f) histidine ‐CH singlet, (g) phenylalanine (‐CH)_2_ doublet, (h) lactate ‐CH_3_ doublet, (i) acetate ‐CH_3_ singlet, (j) pyruvate ‐CH_3_ singlet, (k) formate ‐CH singlet, (l) 3‐hydroxybutyrate ‐CH_3_ doublet, (m) glucose anomeric proton doublet, (n) ethanol ‐CH_3_ triplet, and (o) acetone (‐CH_3_)_2_ singlet from both ethanol (black diamonds) and pyruvate (blue diamonds). (p, q, r) Examples of plotted predicted chemical shifts for various spin systems on the validation spectra (extra examples are depicted in Figure S19). Plotting was achieved by calibrating the spectrum (blue line) at the predicted (orange line) ppm value of each spin system.

To further validate the performance of the linear regression models, we employed an independent cohort, consisting of 1,052 plasma‐EDTA samples/NMR spectra. Initially, the algorithm was applied in a semi‐automated/user‐guided way, namely, the input of each model was the assigned *δ* (i.e., real *δ*) for each spin system in the 1,052 spectra. For example, the observed valine ‐CH_3_ real *δ* was used for the prediction of leucine, alanine, isoleucine, and tyrosine, and the predicted values were compared with the assigned values (Figure 7a–d), demonstrating an accuracy within ±0.5 Δ*v*_1/2_. As previously mentioned, the sequential predictions were based upon the real *δ* of each predictor; namely, lactate was predicted by the assigned ‐CH_3_ group of leucine, and so forth. The results of the semi‐automated predictions are summarized in Figure 7, where the maximum error range was <1.5 Δ*v*_1/2_ for all spins systems in all cases (Figure 7a–e,g–o), except for histidine that was predicted within 3 Δ*v*_1/2_ (Figure 7f), as expected from the accuracy of the corresponding model (Figures 2 and 3g).

Strikingly, the automated prediction of 15 metabolites' *δ*, relying solely on the ‐CH_3_ of valine, resulted into the same accuracy (Figure S18a–k) as in the case of the semi‐automated procedure. Predicted *δ*s of histidine (Figure S18b) and glucose (Figure S18i) were slightly worse, increasing the error margin up to ±0.0008 and ±0.0020 ppm compared to previous ±0.0005 and ±0.0015 ppm, respectively. However, acetone prediction from pyruvate was improved from ±0.0010 to ±0.0008 ppm error range, and all other predictions remained as precise as before or even slightly improved.

Even though our algorithm was built upon serum NMR profiles, validation results on plasma samples supported our models highly accurate predicting ability and corroborated our strategy for metabolites' signals automated detection via “mapping” spins systems relationships through chemistry related homologies/criteria. Moreover, the automated *δ* predictions indicate that the constructed linear regression functions could be applicable to any serum/plasma dataset provided that the NMR samples/spectra are prepared/acquired under the same SOPs. Yet, in theory (needs to be explored), the developed linear relationships between the dependent variables (y) and the regressors (x) should be pertinent for the spin systems relationships from similar classes of metabolites regardless of the applied SOPs. It is noteworthy that the algorithm requires minimal computational resources, that is, <5 s to predict 15‐till now‐spin systems *δ* from, for example, 2,000 serum/plasma spectra using a conventional laptop.

### 3.3 Limitations of models' applicability and future challenges

The main goal of our study was to prove that the chemical shifts of several chemically homologous protons from similar groups of metabolites experience the same matrix effects and therefore that linear regression models can be employed to predict ^1^H NMR *δ* within high accuracy. Indeed, the current results supported this concept. One limitation is that our models can currently be applied only in serum/plasma NMR spectra following the above‐mentioned SOPs (see Section 2). The proposed strategy applies for known metabolites, whose signals are previously assigned in plenty NMR spectra of the same matrix, and it requires the average ppm value for the multiplets. The expansion of our models to the prediction of a larger number of chemical shifts (on‐going work) could provide either more or less accurate models (even for the present metabolites), depending on the kind of matrix and its composition complexity. Additionally, where metabolite signals fall in regions of the spectrum containing a high density of peaks, the training dataset would require multi‐dimensional NMR experiments for accurate assignment and modeling. Despite the high number of spectra in the validation dataset, the automated application of our models should be further tested in more spectra to further validate their accuracy. Overall, our proposed “maps” could be followed to validate and even expedite the assignment of metabolites by currently existing software, even though further future work is needed for this to be widely applicable.

## 4 CONCLUSIONS

In summary, we have introduced a strategy to simplify and expedite the robust assignment of metabolite ^1^H NMR chemical shifts in an automated way. Our strategy relies upon the assumption that similar chemical groups from the same class of molecules should experience similarly any matrix effects and therefore be linearly correlated. The training dataset of the assigned 16 spin systems *δ* from 16 metabolites based upon 940 serum spectra allowed us to construct 240 1:1 spin system *δ* linear regression models, demonstrating high accuracy and predicting ability. The successful combination of these models led to the construction of a general strategy, presented as a “map” of combined linear models, that allows the prediction of the chemical shifts from 15 metabolites, depending on valine's methyl protons signal. Our methodology sets the grounds of a general future algorithm, enriched with several kinds of metabolites *δ* models, guided by chemically as well as matrix effects‐based criteria upon the described limitations. The proposed method has exclusive advantages and significantly contributes to the NMR‐based metabolomics pipeline by the sizable decrease of computational time required to reliably identify several serum/plasma metabolites, without computationally expensive algorithms, construction of databases, and laborious manual assignments. Extensive validation of both the fully automated and semi‐automated metabolite identification strategies on an independent plasma cohort showed high accuracy, while requiring on average 5 s or less per ~2,000 spectra on a conventional laptop. Finally, our study also verifies that simple relationships between chemically homologous protons from various classes of molecules, beyond the studied metabolites, could be established (work in progress) to predict their chemical shifts in various biofluids. The presented methodology/models could be used as such for serum/plasma data acquired under the same SOPs as herein.

## CONFLICT OF INTEREST STATEMENT

The authors declare no conflict of interest.

### PEER REVIEW

The peer review history for this article is available at https://www.webofscience.com/api/gateway/wos/peer-review/10.1002/mrc.5392.

## Supporting information


**Table S1.** The 16 metabolites' structure, ^1^H NMR spins systems (red circles) fingerprint, and their signals multiplicity employed for the study.
**Figure S1.** Examples of statistical correlation spectroscopy (STOCSY) application for the assignment of various metabolites signals used for the study (black boxes): (A) 3‐hydroxybutyrate, (B) histidine, (C) ethanol, (D) phenylalanine and (E) tyrosine. Examples of spiking experiments for the assignment of metabolites exhibiting only singlets: (F) acetone, (G) acetate and (H) pyruvate.
**Figure S2.** Scatter plots and fitted liner regression lines (y = a*x + b) for all spins systems with alanine ‐CH_3_ *δ* as the predictor (x). For each fitted model, the calculated R^2^ and RMSE values are depicted.
**Figure S3.** Scatter plots and fitted liner regression lines (y = a*x + b) for all spins systems with lactate ‐CH_3_ *δ* as the predictor (x). For each fitted model, the calculated R^2^ and RMSE values are depicted.
**Figure S4.** Scatter plots and fitted liner regression lines (y = a*x + b) for all spins systems with valine ‐CH_3_ *δ* as the predictor (x). For each fitted model, the calculated R^2^ and RMSE values are depicted.
**Figure S5.** Scatter plots and fitted liner regression lines (y = a*x + b) for all spins systems with isoleucine ‐CH_3_ *δ* as the predictor (x). For each fitted model, the calculated R^2^ and RMSE values are depicted.
**Figure S6.** Scatter plots and fitted liner regression lines (y = a*x + b) for all spins systems with glucose anomeric proton *δ* as the predictor (x). For each fitted model, the calculated R^2^ and RMSE values are depicted.
**Figure S7.** Scatter plots and fitted liner regression lines (y = a*x + b) for all spins systems with acetone (‐CH_3_)_2_ *δ* as the predictor (x). For each fitted model, the calculated R^2^ and RMSE values are depicted.
**Figure S8.** Scatter plots and fitted liner regression lines (y = a*x + b) for all spins systems with leucine (‐CH_3_)_2_ *δ* as the predictor (x). For each fitted model, the calculated R^2^ and RMSE values are depicted.
**Figure S9.** Scatter plots and fitted liner regression lines (y = a*x + b) for all spins systems with acetate ‐CH_3_ *δ* as the predictor (x). For each fitted model, the calculated R^2^ and RMSE values are depicted.
**Figure S10.** Scatter plots and fitted liner regression lines (y = a*x + b) for all spins systems with 3‐hydroxybutyrate ‐CH_3_ *δ* as the predictor (x). For each fitted model, the calculated R^2^ and RMSE values are depicted.
**Figure S11.** Scatter plots and fitted liner regression lines (y = a*x + b) for all spins systems with ethanol ‐CH_3_ *δ* as the predictor (x). For each fitted model, the calculated R^2^ and RMSE values are depicted.
**Figure S12.** Scatter plots and fitted liner regression lines (y = a*x + b) for all spins systems with formate ‐CH *δ* as the predictor (x). For each fitted model, the calculated R^2^ and RMSE values are depicted.
**Figure S13.** Scatter plots and fitted liner regression lines (y = a*x + b) for all spins systems with histidine ‐CH *δ* as the predictor (x). For each fitted model, the calculated R^2^ and RMSE values are depicted.
**Figure S14.** Scatter plots and fitted liner regression lines (y = a*x + b) for all spins systems with phenylalanine (‐CH)_2_ *δ* as the predictor (x). For each fitted model, the calculated R^2^ and RMSE values are depicted.
**Figure S15.** Scatter plots and fitted liner regression lines (y = a*x + b) for all spins systems with tyrosine (‐CH)_2_ *δ* as the predictor (x). For each fitted model, the calculated R^2^ and RMSE values are depicted.
**Figure S16.** Scatter plots and fitted liner regression lines (y = a*x + b) for all spins systems with pyruvate ‐CH_3_ *δ* as the predictor (x). For each fitted model, the calculated R^2^ and RMSE values are depicted.
**Figure S17.** Scatter plots and fitted liner regression lines (y = a*x + b) for all spins systems with glycine ‐CH_2_ *δ* as the predictor (x). For each fitted model, the calculated R^2^ and RMSE values are depicted.
**Figure S18.** The performance of the final “map” (see Figure 6 of the main article) for the **automated** prediction of the studied spin systems tested in maximum 1,052 plasma‐EDTA spectra (i.e., the independent validation dataset). In particular, the real *δ* (i.e., assigned) values are plotted against the predicted values (in ppm). The ± error bars indicate the maximum calculated error of each model, based upon the validation datasets real *δ*. Red lines indicate the 1:1 line (i.e., perfect line). Results are for the following metabolites spin systems: (A) glycine, (B) histidine, (C) phenylalanine, (D) lactate, (E) acetate, (F) pyruvate, (G) formate, (H) 3‐hydroxybutyrate, (I) glucose, (J) ethanol and (K) acetone.
**Figure S19.** Examples of various predicted ^1^H NMR chemical shifts (red line) plotted versus the real NMR profile. Plotting was achieved by calibrating the spectrum at the predicted ppm value of each spin system.

## Data Availability

The linear regression functions of the described maps are included in the supporting information, and the employed spectra for the construction of our models are freely available in the Metabolights repository (https://www.ebi.ac.uk/metabolights) (MTBLS395).
