# Supplementary material for: Mapping of 1H NMR chemical shifts relationship with chemical similarities for the acceleration of metabolic profiling: Application on blood products
Source: Magn Reson Chem. 2023 Sep 4;61(12):759–69. doi: 10.1002/mrc.5392 (PMC10946494; doi:10.1002/mrc.5392)
Supplement: Supplementary file 1 — Table S1. The 16 metabolites' structure, 1H NMR spins systems (red circles) fingerprint, and their signals multiplicity employed for the study. Figure S1. Examples of statistical correlation spectroscopy (STOCSY) application for the assignment of various metabolites signals used for the study (black boxes): (A) 3‐hydroxybutyrate, (B) histidine, (C) ethanol, (D) phenylalanine and (E) tyrosine. Examples of spiking experiments for the assignment of metabolites exhibiting only singlets: (F) acetone, (G) acetate and (H) pyruvate. Figure S2. Scatter plots and fitted liner regression lines (y = a*x + b) for all spins systems with alanine ‐CH3 δ as the predictor (x). For each fitted model, the calculated R2 and RMSE values are depicted. Figure S3. Scatter plots and fitted liner regression lines (y = a*x + b) for all spins systems with lactate ‐CH3 δ as the predictor (x). For each fitted model, the calculated R2 and RMSE values are depicted. Figure S4. Scatter plots and fitted liner regression lines (y = a*x + b) for all spins systems with valine ‐CH3 δ as the predictor (x). For each fitted model, the calculated R2 and RMSE values are depicted. Figure S5. Scatter plots and fitted liner regression lines (y = a*x + b) for all spins systems with isoleucine ‐CH3 δ as the predictor (x). For each fitted model, the calculated R2 and RMSE values are depicted. Figure S6. Scatter plots and fitted liner regression lines (y = a*x + b) for all spins systems with glucose anomeric proton δ as the predictor (x). For each fitted model, the calculated R2 and RMSE values are depicted. Figure S7. Scatter plots and fitted liner regression lines (y = a*x + b) for all spins systems with acetone (‐CH3)2 δ as the predictor (x). For each fitted model, the calculated R2 and RMSE values are depicted. Figure S8. Scatter plots and fitted liner regression lines (y = a*x + b) for all spins systems with leucine (‐CH3)2 δ as the predictor (x). For each fitted model, the calculated R2 and RMSE values are depi [file MRC-61-759-s001.pdf]

Mapping of ^{1}H NMR chemical shifts relationship with chemical similarities for the acceleration of metabolic profiling – Application on blood products

Panteleimon G. Takis[a,b]*, Varvara A. Aggelidou[c], Caroline J. Sands[a,b] and Alexandra Louka[d]

[a] Section of Bioanalytical Chemistry, Division of Systems Medicine, Department of Metabolism, Digestion and Reproduction, Imperial College London, South Kensington Campus, London, SW7 2AZ, UK
[b] National Phenome Centre, Department of Metabolism, Digestion and Reproduction, Imperial College London, Hammersmith Campus, IRDB Building, London, W12 0NN, UK.
[c] Department of Biological Applications and Technologies, University of Ioannina, 45110 Ioannina, Greece
[d] Department of Clinical and Experimental Epilepsy, Queen Square Institute of Neurology, University College London, London, WC1N 3BG, UK

*To whom correspondence should be addressed: p.takis@imperial.ac.uk

Table of Contents

Structural/NMR characteristics of the studied metabolites ***3***

Table S1. 3

STOCSY, spiking examples for assignments validation ***5***

Alanine as predictor (x) ***8***

Figure S2. 10

Lactate as predictor (x) ***11***

Figure S3. 13

Valine as predictor (x) ***14***

Figure S4. 16

Isoleucine as predictor (x) ***17***

Figure S5. 19

Glucose as predictor (x) ***20***

Figure S6. 22

Acetone as predictor (x) ***23***

Figure S7. 25

Leucine as predictor (x) ***26***

Figure S8. 28

Acetate as predictor (x) ***29***

Figure S9. 31

3-hydroxybutyrate as predictor (x) ***32***

Figure S10. 34

Ethanol as predictor (x) ***35***

Figure S11. 37

Formate as predictor (x) ***38***

Figure S12. 40

Histidine as predictor (x) ***41***

Figure S13. 43

Phenylalanine as predictor (x) ***44***

Figure S14. 46

Tyrosine as predictor (x) ***47***

Figure S15. 49

Pyruvate as predictor (x) ***50***

Figure S16. 52

Glycine as predictor (x) ***53***

Figure S17. 55

Predicting δ in an automated way ***56***

Figure S18. 56

Examples of chemical shifts predictions on one validation spectrum ***57***

Figure S19. 57

Linear regression functions from the best models and corresponding statistics ***58***

Structural/NMR characteristics of the studied metabolites

Table S1. The 16 metabolites' structure, ^{1}H NMR spins systems (red circles) fingerprint, and their signals multiplicity employed for the study.

Metabolite	Molecular formula	NMR fingerprint	Type of Signal/ Peak's δ
Alanine (Ala)	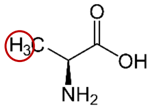	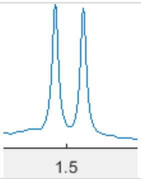	Doublet
Lactate (Lac)	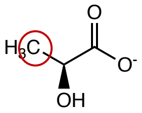	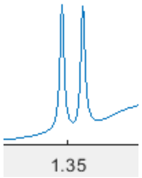	Doublet
Valine (Val)	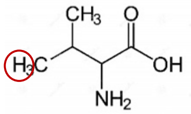	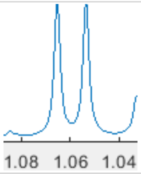	Doublet
Isoleucine (Ile)	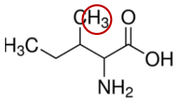	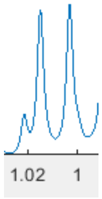	Doublet
Leucine (Leu)	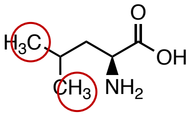	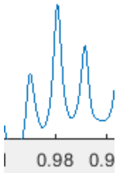	Triplet
Glucose (Glc)	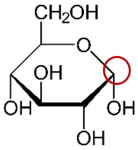	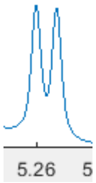	Doublet
Acetone	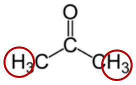	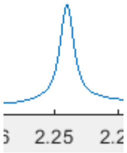	Singlet
Acetate	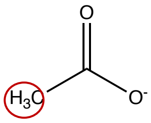	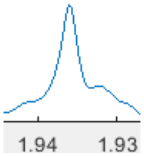	Singlet
3-hydroxybutyrate (BHB)	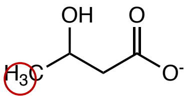	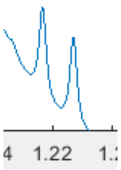	Doublet

Ethanol (EtOH)	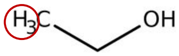	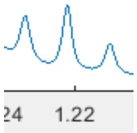	Triplet
Formate	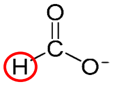	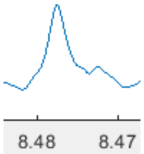	Singlet
Histidine (His)	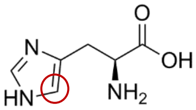	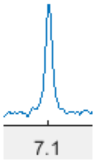	Histidine
Phenylalanine	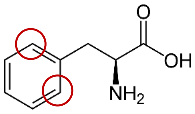	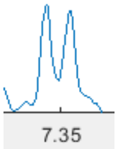	Doublet
Tyrosine	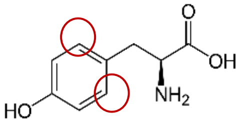	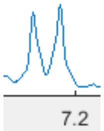	Doublet
Pyruvate	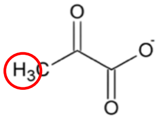	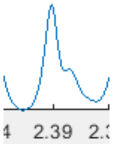	Singlet
Glycine	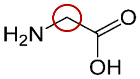	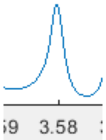	Singlet

STOCSY, spiking examples for assignments validation

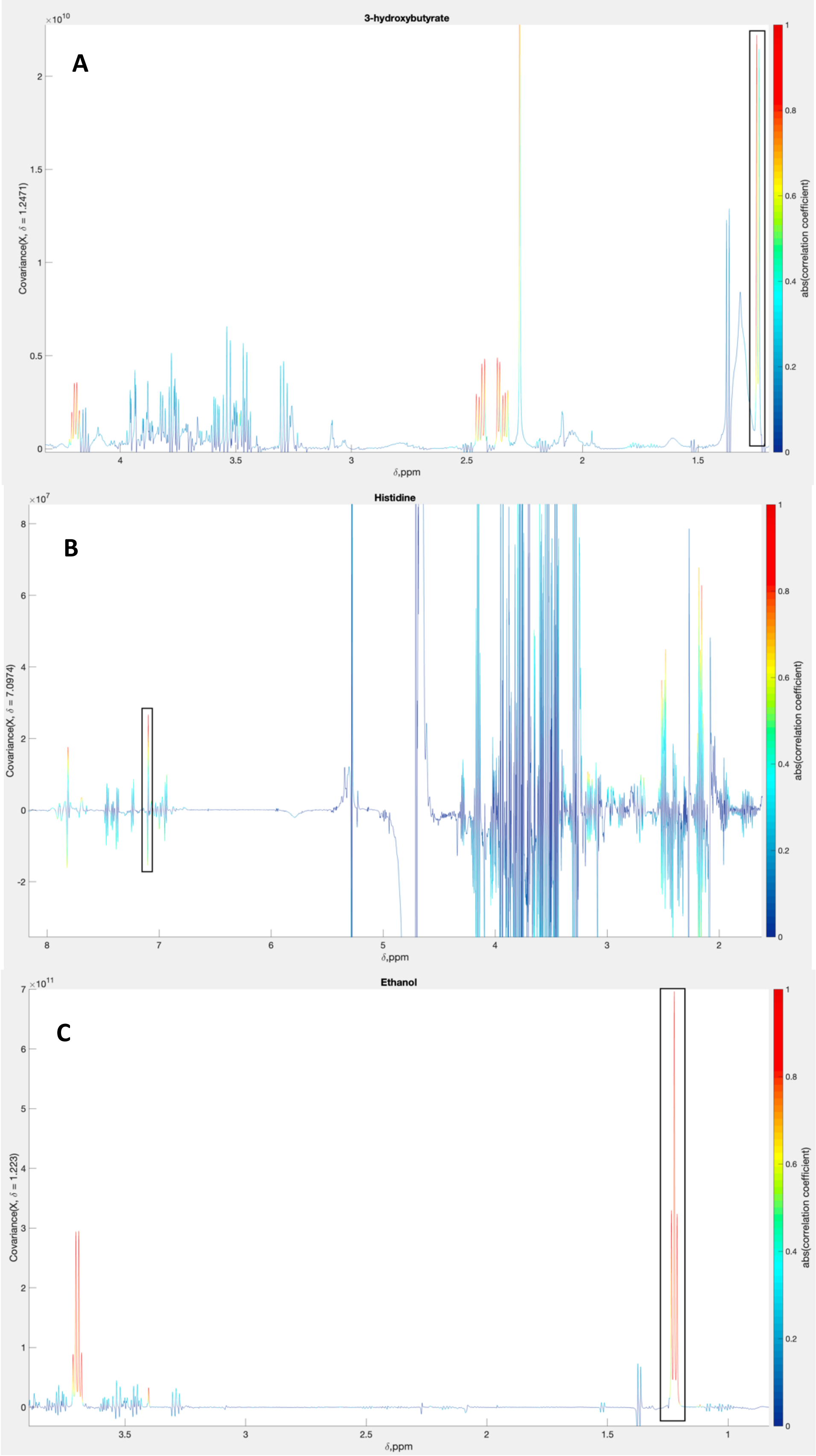

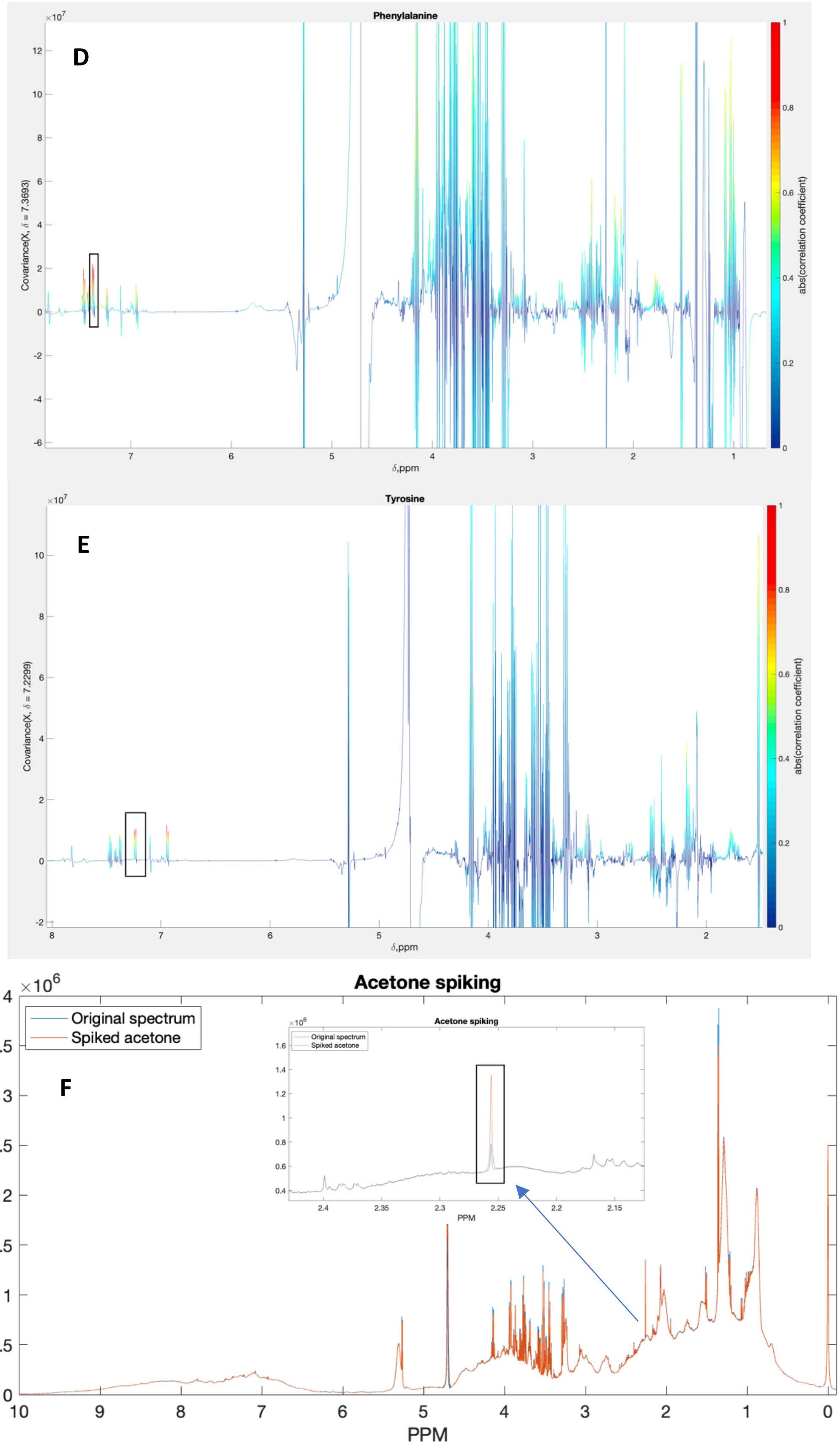
D
Phenylalanine
Covariance(X, δ = 7.3693)
abs(correlation coefficient)
δ,ppm
E
Tyrosine
Covariance(X, δ = 7.2299)
abs(correlation coefficient)
δ,ppm
F
Acetone spiking
Original spectrum
Spiked acetone
PPM

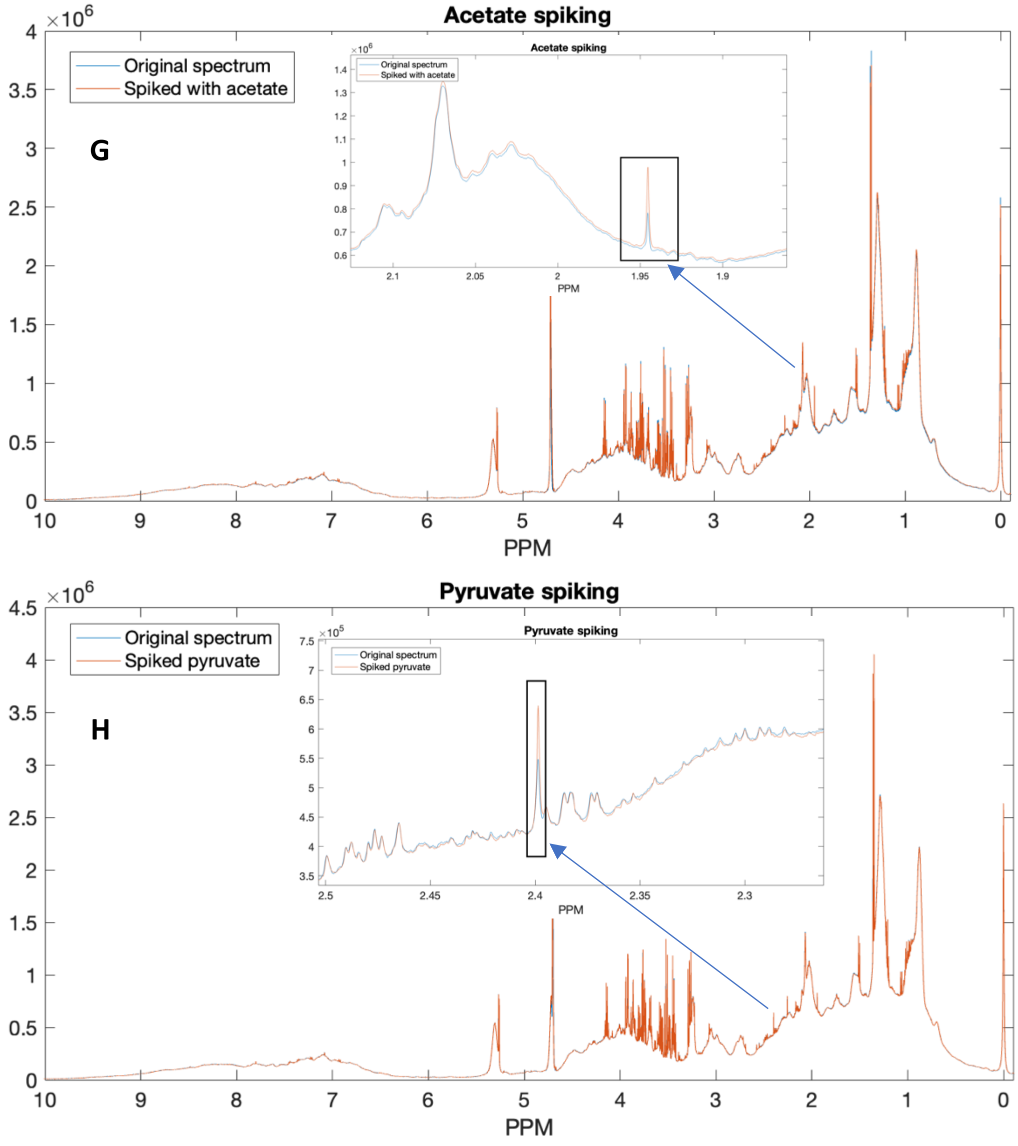

Figure S1. Examples of statistical correlation spectroscopy (STOCSY) application for the assignment of various metabolites signals used for the study (black boxes): (A) 3-hydroxybutyrate, (B) histidine, (C) ethanol, (D) phenylalanine and (E) tyrosine. Examples of spiking experiments for the assignment of metabolites exhibiting only singlets: (F) acetone, (G) acetate and (H) pyruvate.

Alanine as predictor (x)

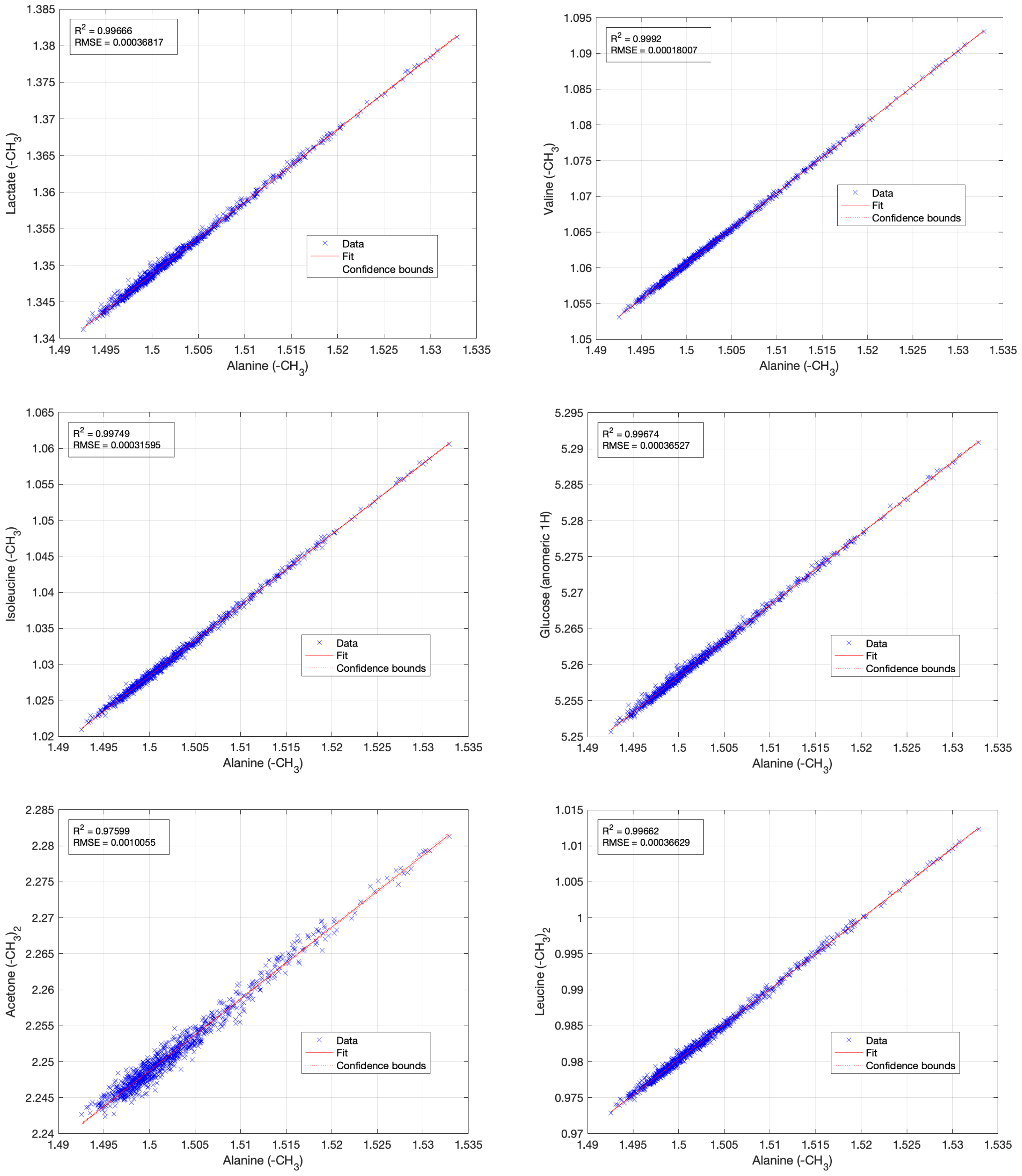

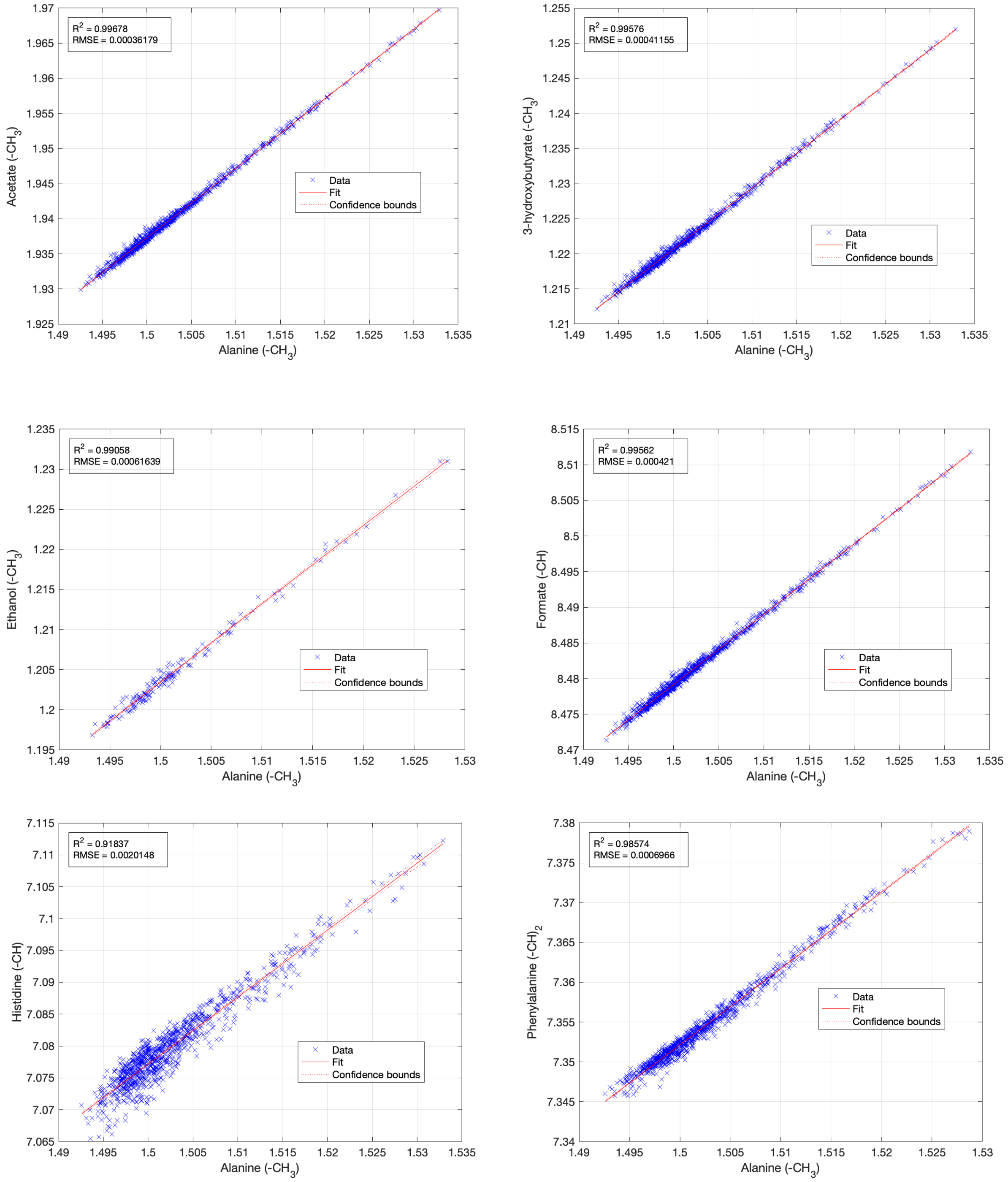
R2 = 0.99678
RMSE = 0.00036179
Acetate (-CH3)
Alanine (-CH3)
Data
Fit
Confidence bounds
R2 = 0.99576
RMSE = 0.00041155
3-hydroxybutyrate (-CH3)
Alanine (-CH3)
Data
Fit
Confidence bounds
R2 = 0.99058
RMSE = 0.00061639
Ethanol (-CH3)
Alanine (-CH3)
Data
Fit
Confidence bounds
R2 = 0.99562
RMSE = 0.000421
Formate (-CH)
Alanine (-CH3)
Data
Fit
Confidence bounds
R2 = 0.91837
RMSE = 0.0020148
Histidine (-CH)
Alanine (-CH3)
Data
Fit
Confidence bounds
R2 = 0.98574
RMSE = 0.0006966
Phenylalanine (-CH)2
Alanine (-CH3)
Data
Fit
Confidence bounds

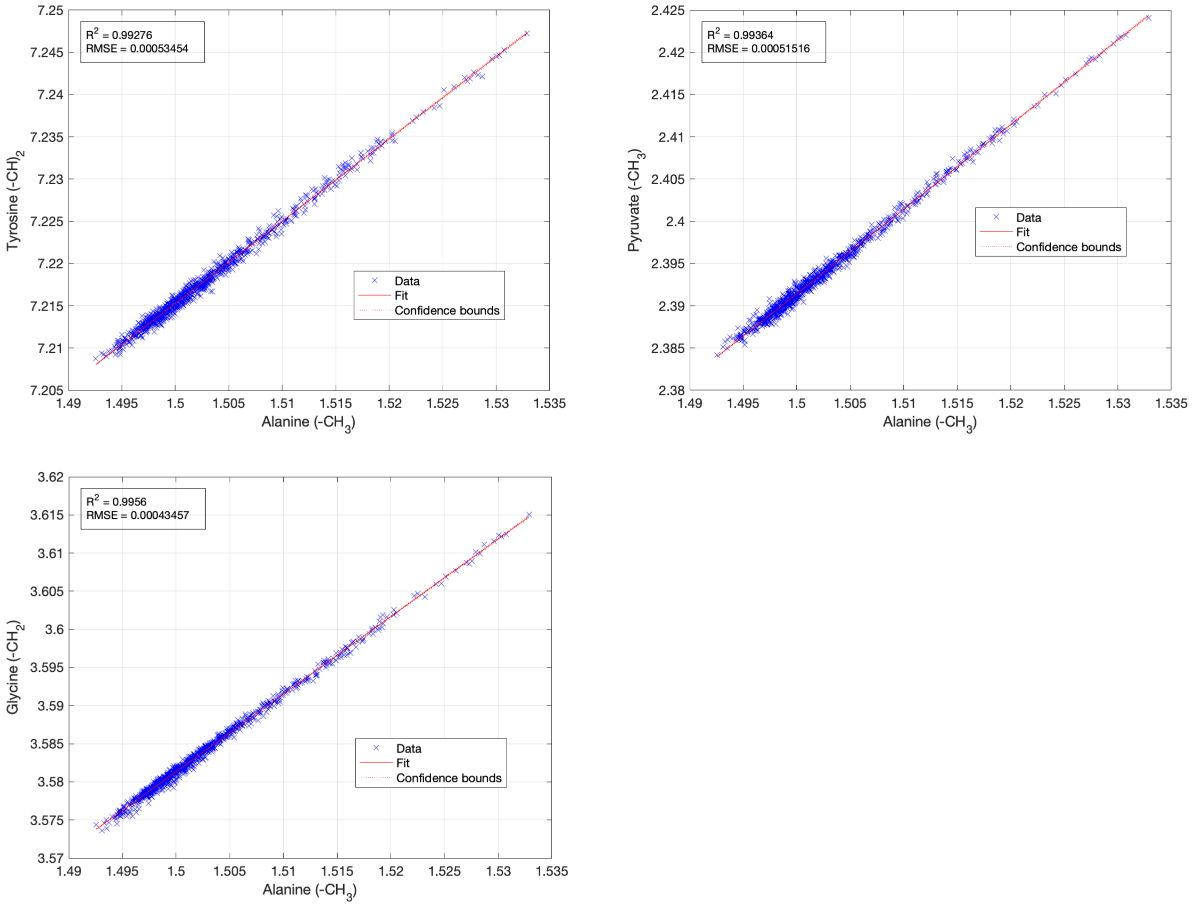

Figure S2. Scatter plots and fitted liner regression lines (y = a*x + b) for all spins systems with alanine $-CH_3$ δ as the predictor(x). For each fitted model, the calculated R^2 and RMSE values are depicted.

Lactate as predictor (x)

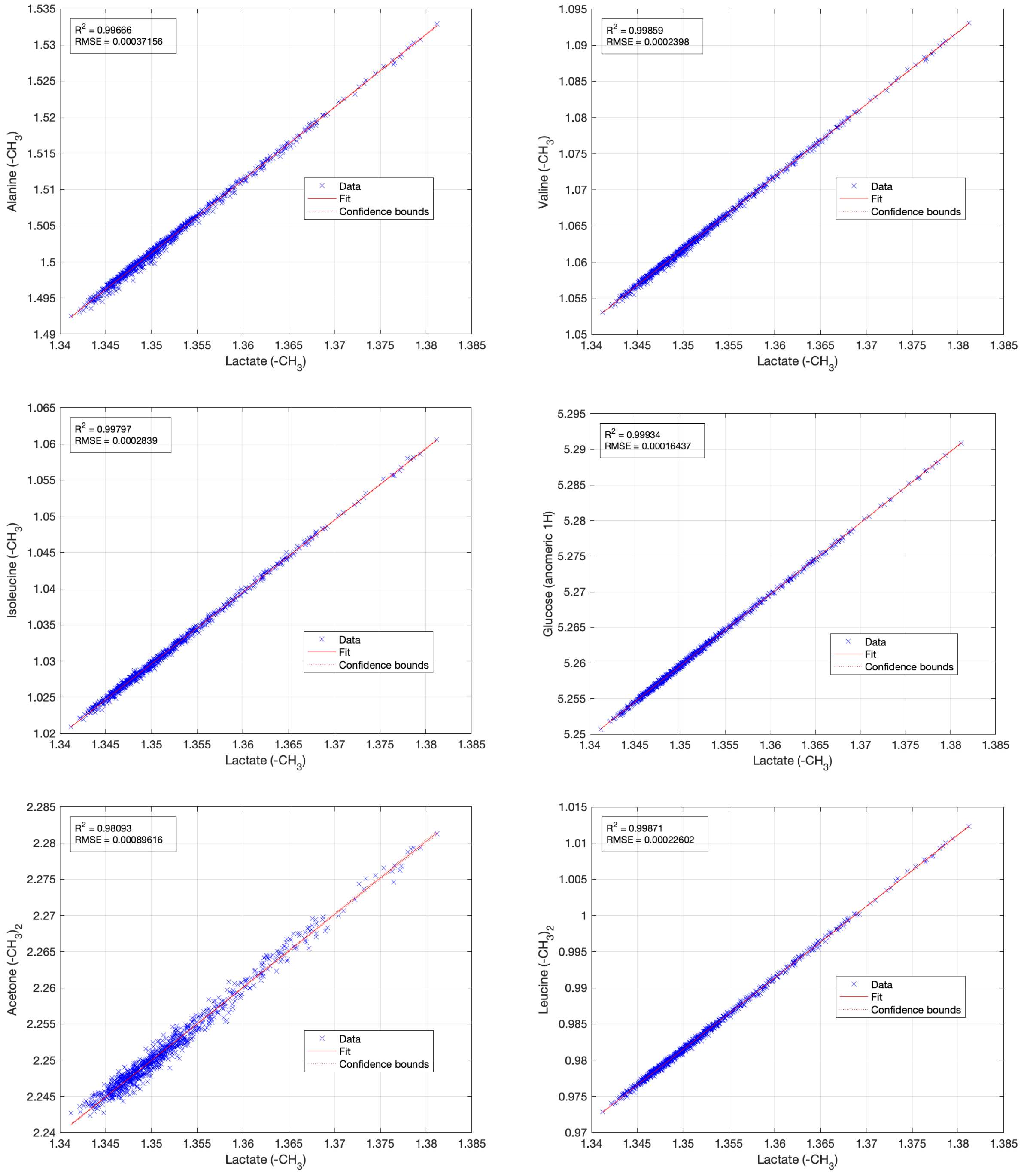

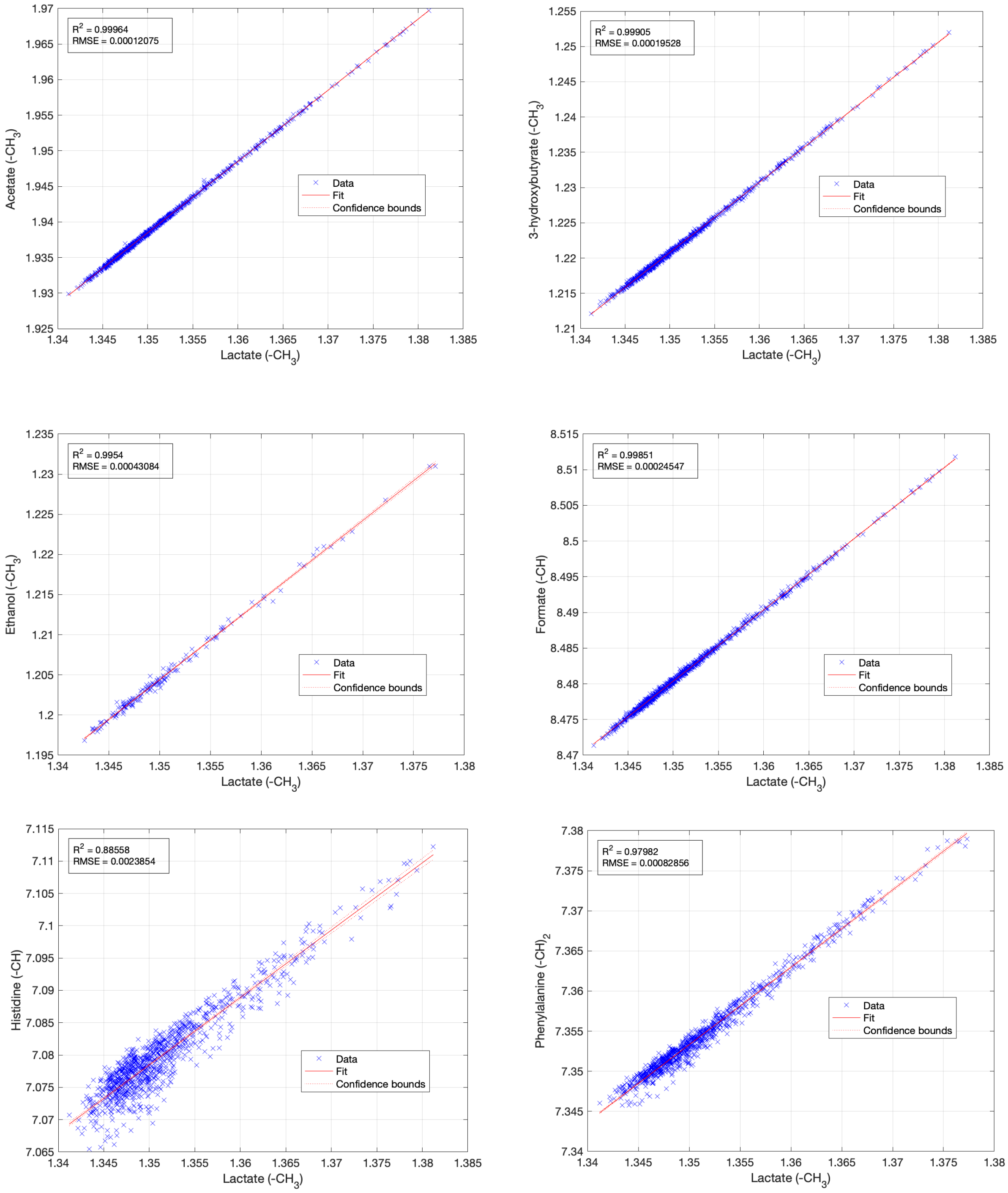

R2 = 0.99964
RMSE = 0.00012075
Acetate (-CH3)
Lactate (-CH3)
R2 = 0.99905
RMSE = 0.00019528
3-hydroxybutyrate (-CH3)
Lactate (-CH3)
R2 = 0.9954
RMSE = 0.00043084
Ethanol (-CH3)
Lactate (-CH3)
R2 = 0.99851
RMSE = 0.00024547
Formate (-CH)
Lactate (-CH3)
R2 = 0.88558
RMSE = 0.0023854
Histidine (-CH)
Lactate (-CH3)
R2 = 0.97982
RMSE = 0.00082856
Phenylalanine (-CH)2
Lactate (-CH3)
Data
Fit
Confidence bounds

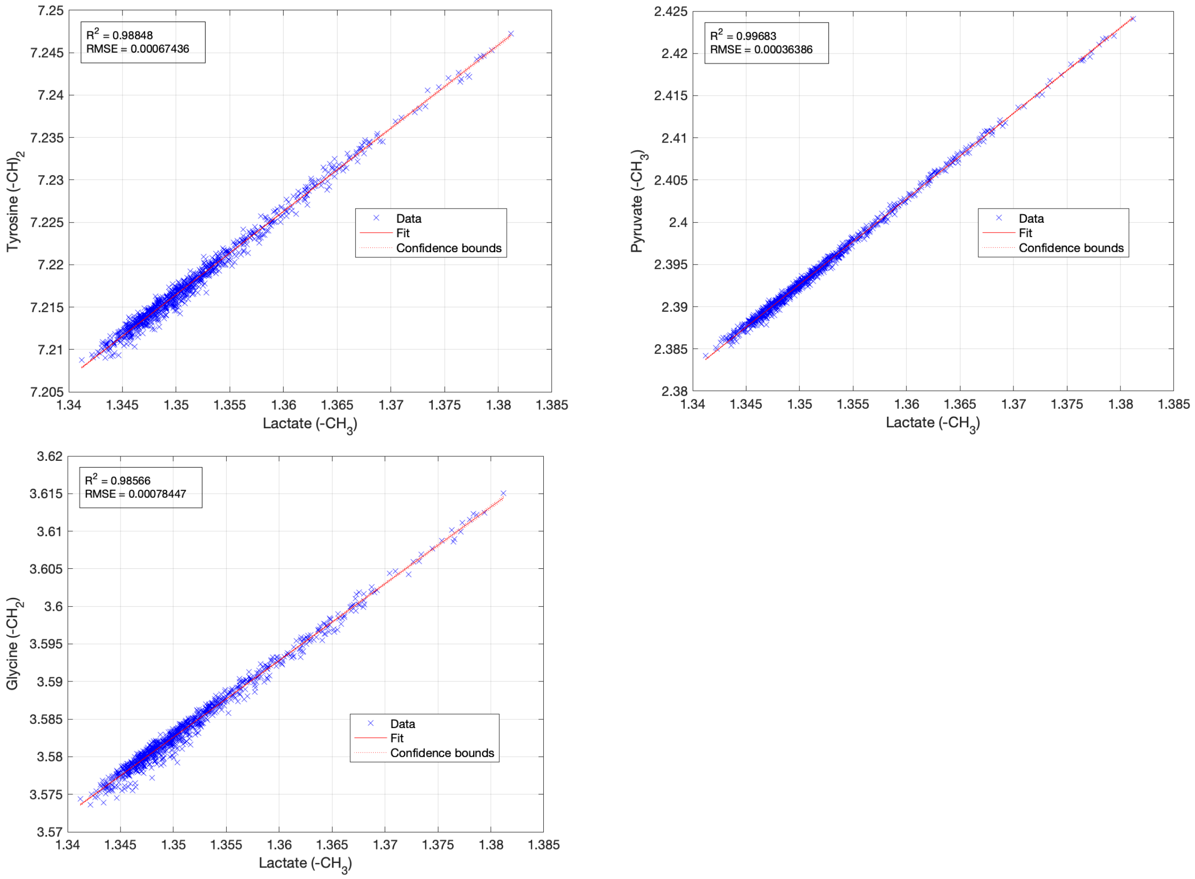

Figure S3. Scatter plots and fitted liner regression lines (y = a*x + b) for all spins systems with lactate -CH_3 δ as the predictor(x). For each fitted model, the calculated R^2 and RMSE values are depicted.

Valine as predictor (x)

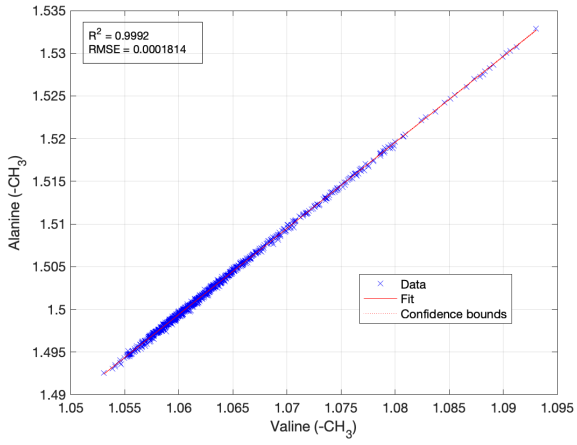

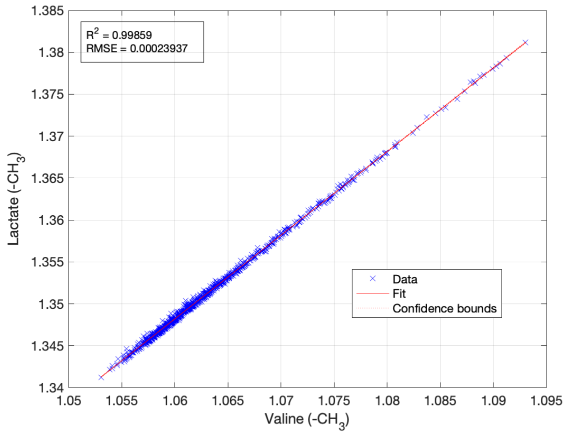

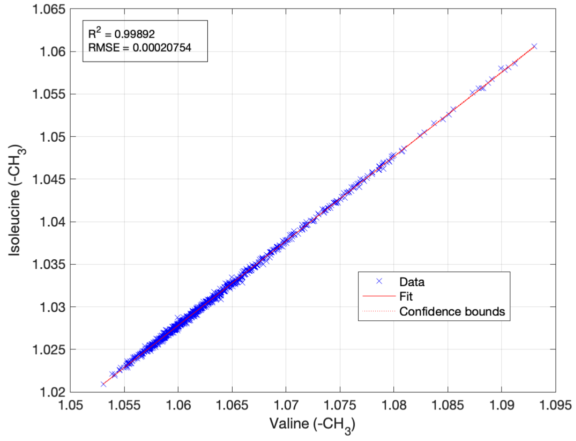

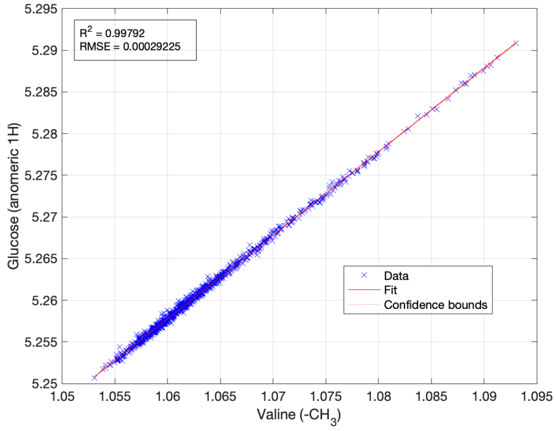

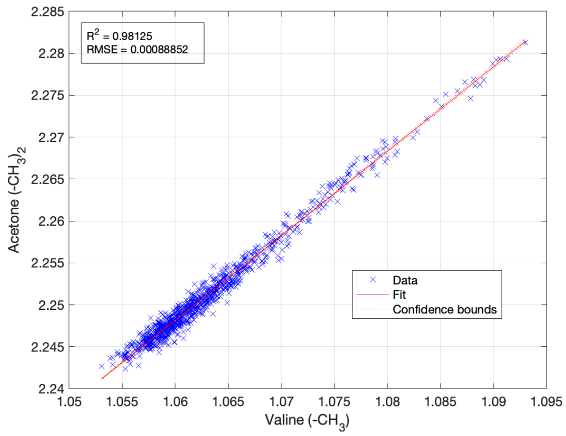

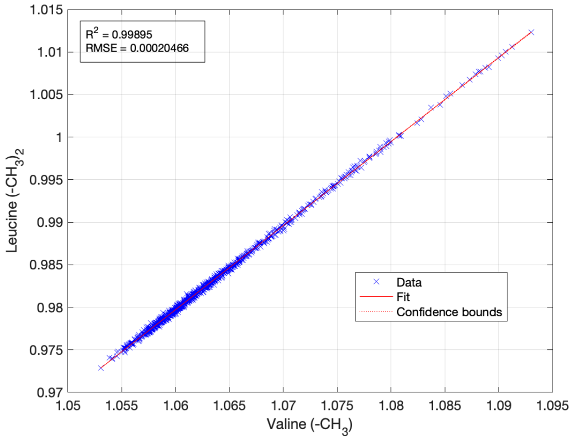

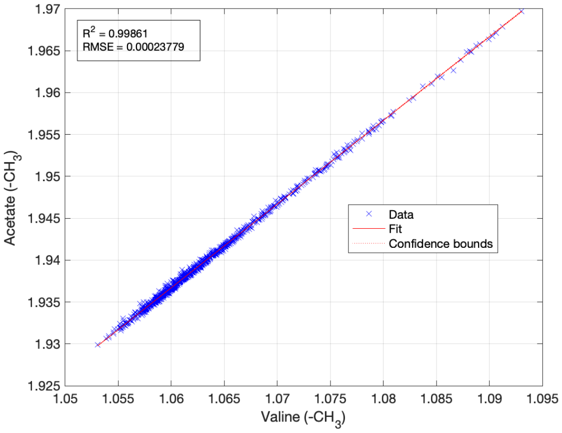

R^2 = 0.99861
RMSE = 0.00023779
Data
Fit
Confidence bounds
Acetate (-CH3)
Valine (-CH3)

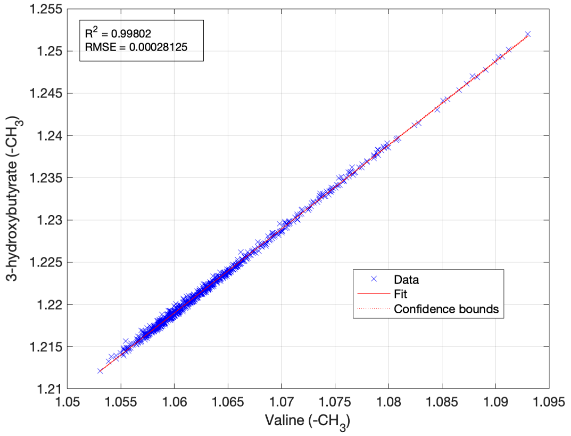

R^2 = 0.99802
RMSE = 0.00028125
Data
Fit
Confidence bounds
3-hydroxybutyrate (-CH3)
Valine (-CH3)

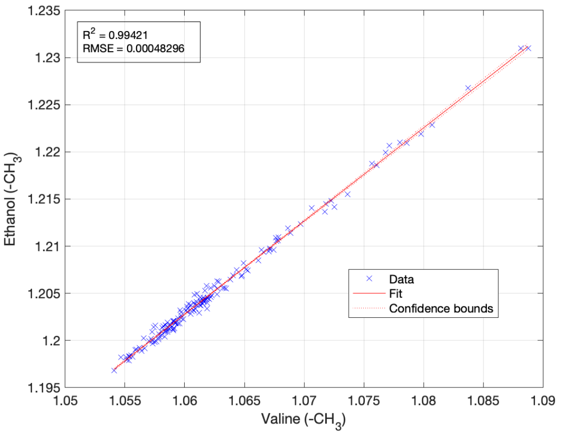

R^2 = 0.99421
RMSE = 0.00048296
Data
Fit
Confidence bounds
Ethanol (-CH3)
Valine (-CH3)

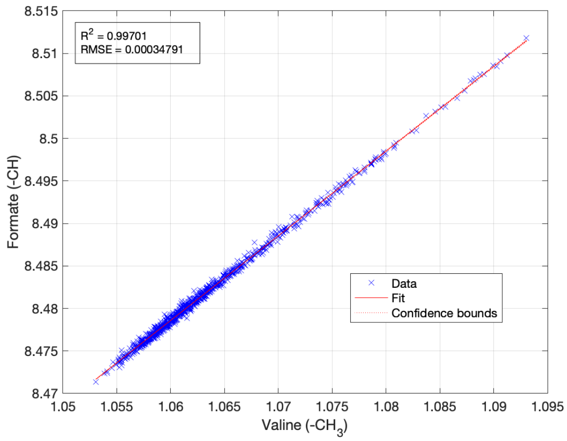

R^2 = 0.99701
RMSE = 0.00034791
Data
Fit
Confidence bounds
Formate (-CH)
Valine (-CH3)

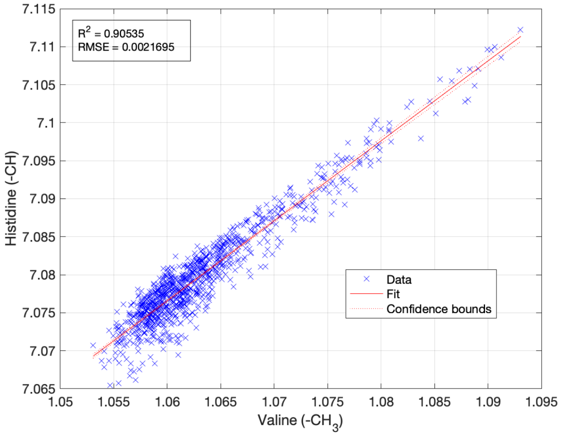

R^2 = 0.90535
RMSE = 0.0021695
Data
Fit
Confidence bounds
Histidine (-CH)
Valine (-CH3)

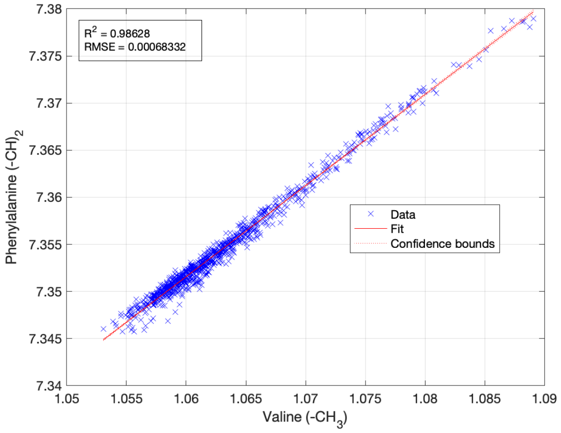

R^2 = 0.98628
RMSE = 0.00068332
Data
Fit
Confidence bounds
Phenylalanine (-CH)2
Valine (-CH3)

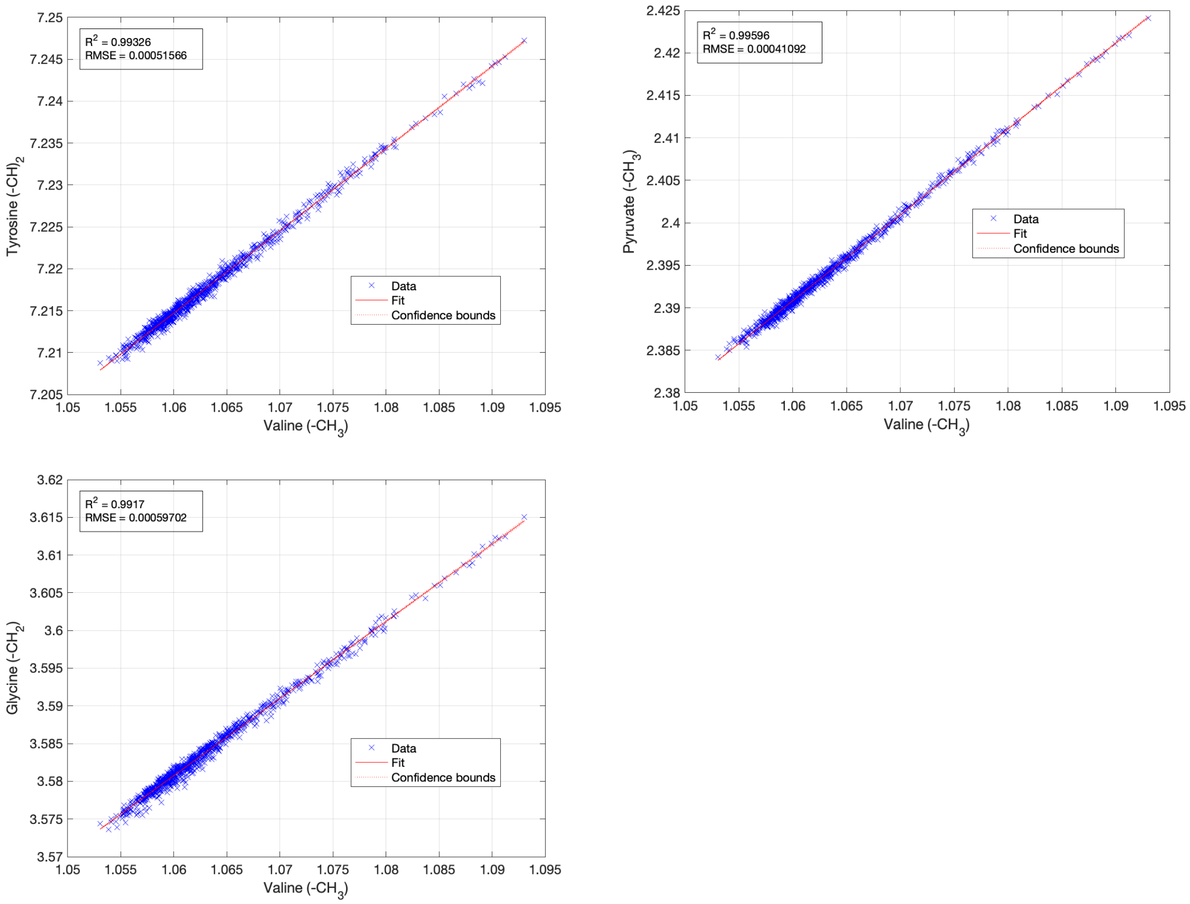

Figure S4. Scatter plots and fitted liner regression lines (y = a*x + b) for all spins systems with valine $-CH_3$ δ as the predictor(x). For each fitted model, the calculated R^2 and RMSE values are depicted.

Isoleucine as predictor (x)

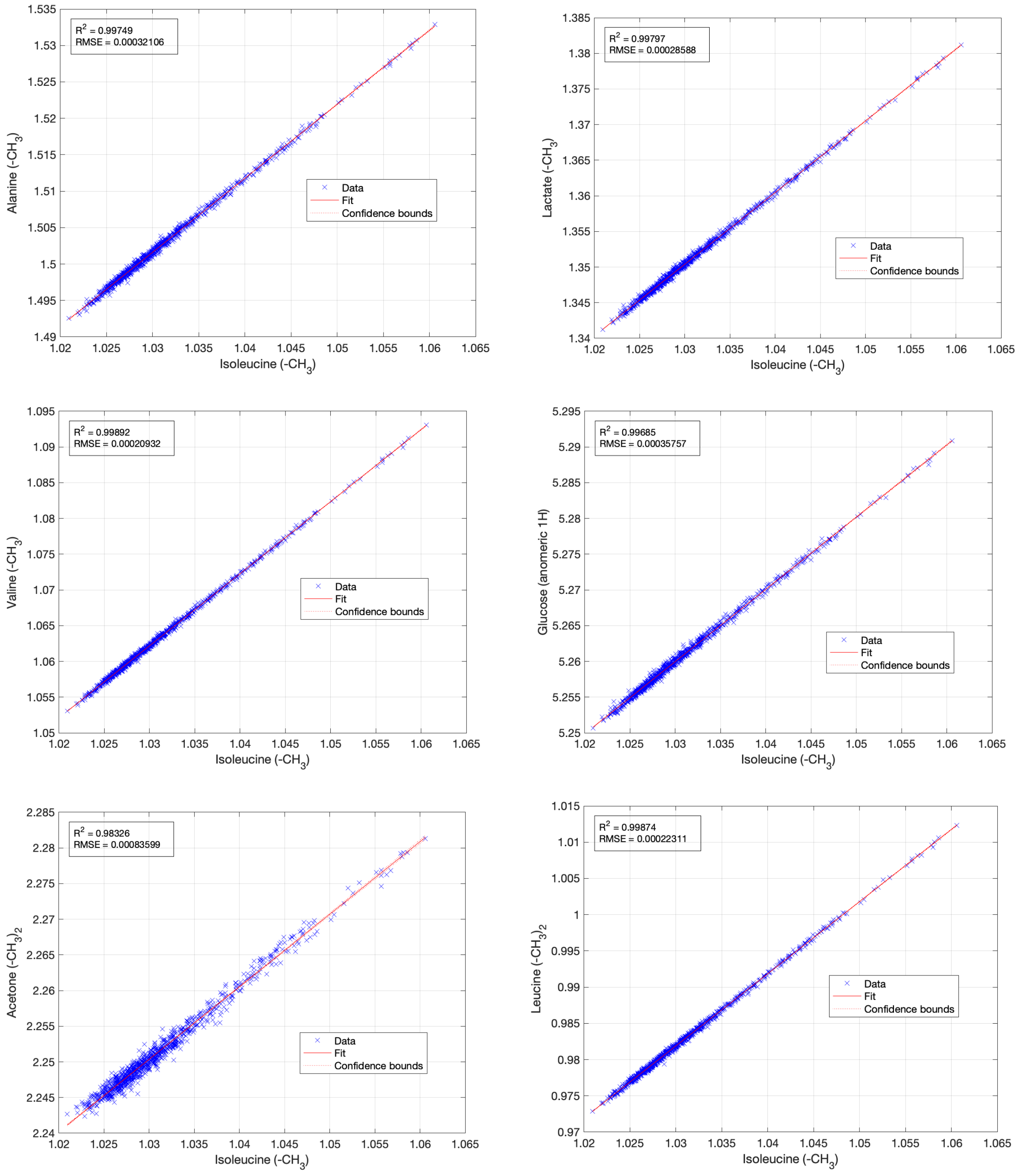

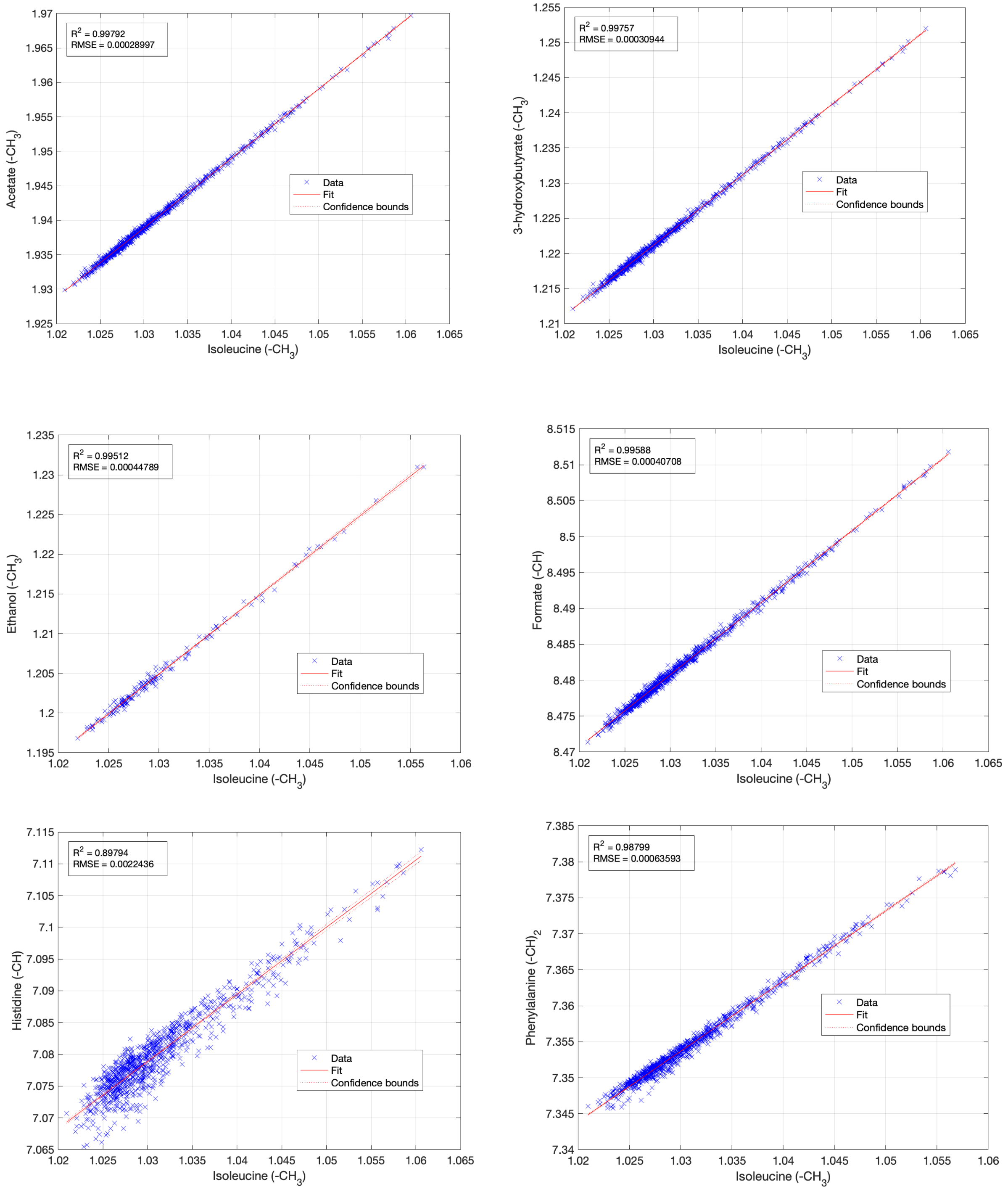
R^2 = 0.99792
RMSE = 0.00028997
Acetate (-CH$_3$)
Isoleucine (-CH$_3$)
Data
Fit
Confidence bounds
R^2 = 0.99757
RMSE = 0.00030944
3-hydroxybutyrate (-CH$_3$)
Isoleucine (-CH$_3$)
Data
Fit
Confidence bounds
R^2 = 0.99512
RMSE = 0.00044789
Ethanol (-CH$_3$)
Isoleucine (-CH$_3$)
Data
Fit
Confidence bounds
R^2 = 0.99588
RMSE = 0.00040708
Formate (-CH)
Isoleucine (-CH$_3$)
Data
Fit
Confidence bounds
R^2 = 0.89794
RMSE = 0.0022436
Histidine (-CH)
Isoleucine (-CH$_3$)
Data
Fit
Confidence bounds
R^2 = 0.98799
RMSE = 0.00063593
Phenylalanine (-CH)$_2$
Isoleucine (-CH$_3$)
Data
Fit
Confidence bounds

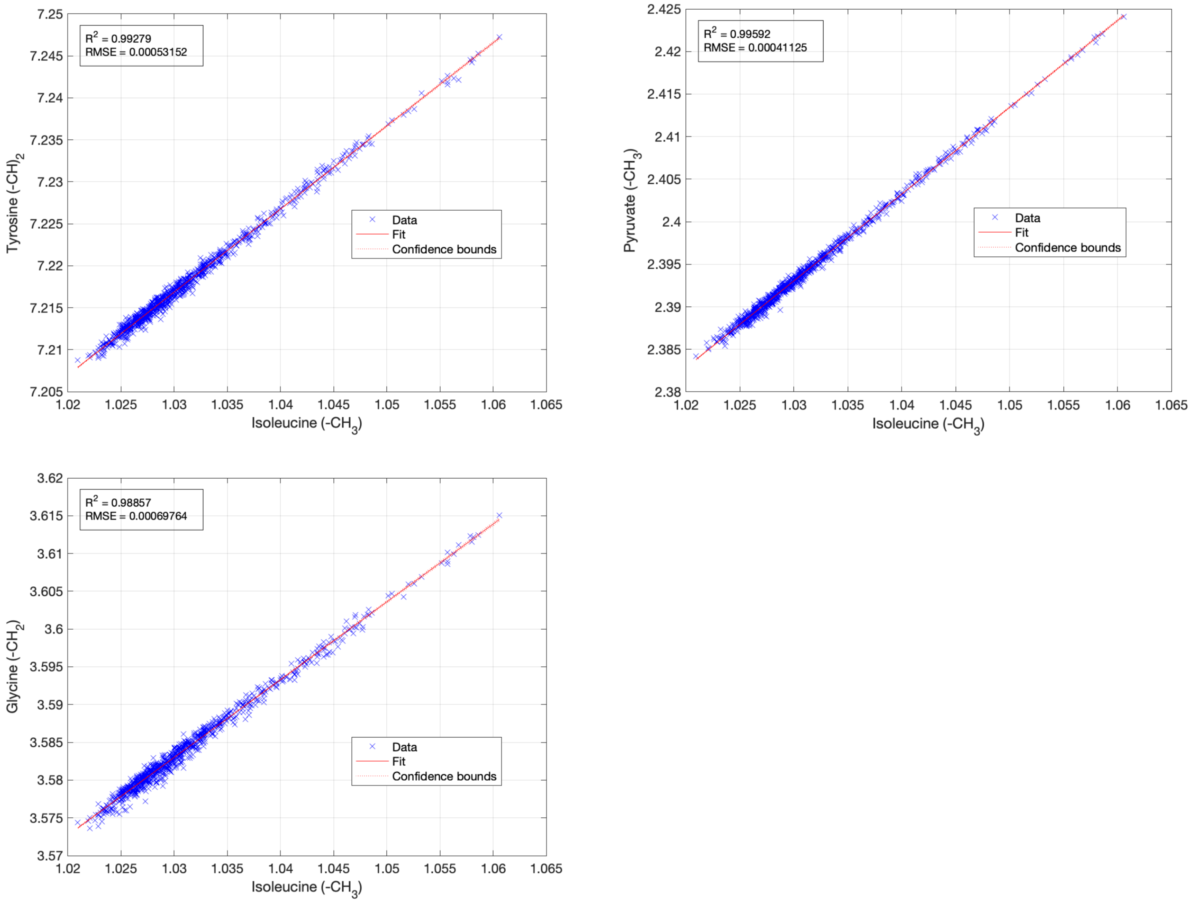

Figure S5. Scatter plots and fitted liner regression lines (y = a*x + b) for all spins systems with isoleucine -CH_3 δ as the predictor(x). For each fitted model, the calculated R^2 and RMSE values are depicted.

Glucose as predictor (x)

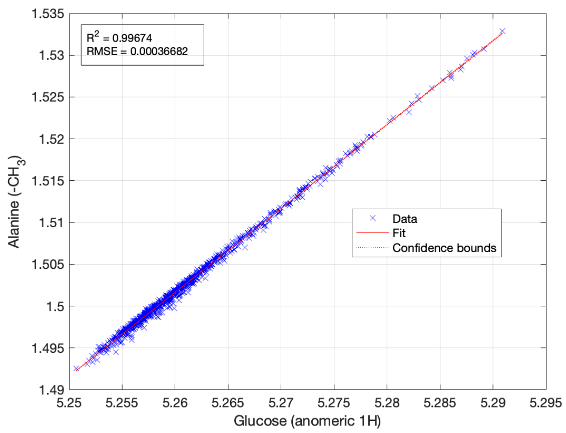

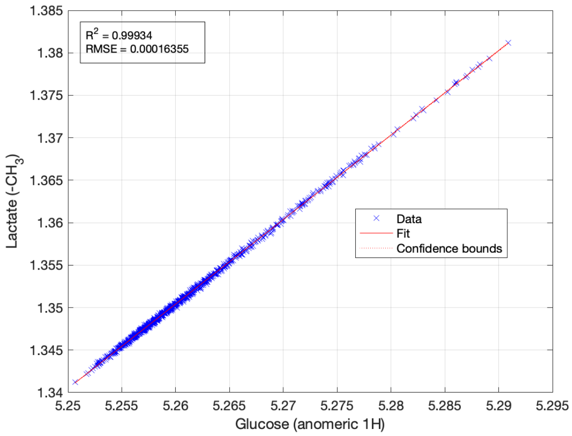

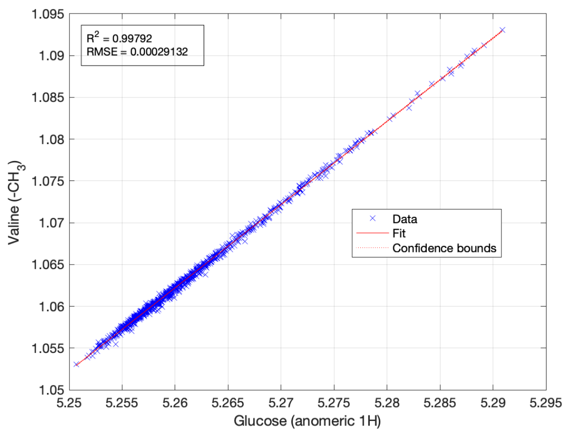

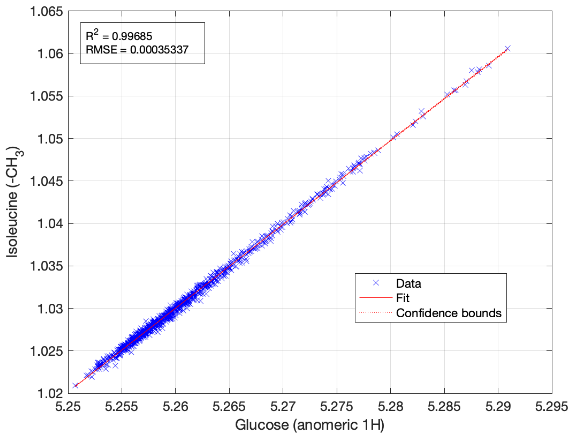

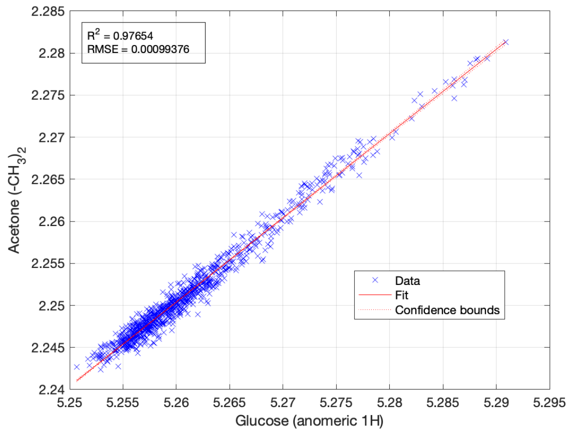

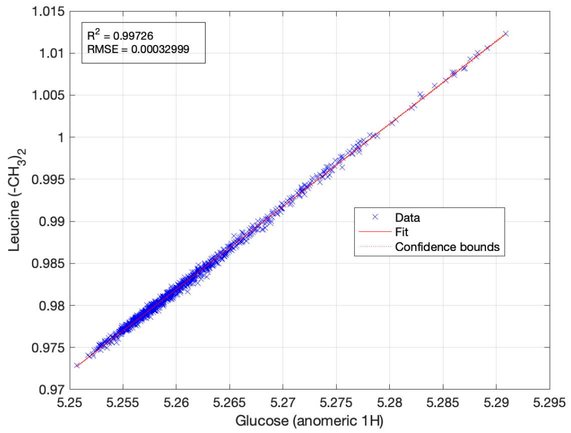

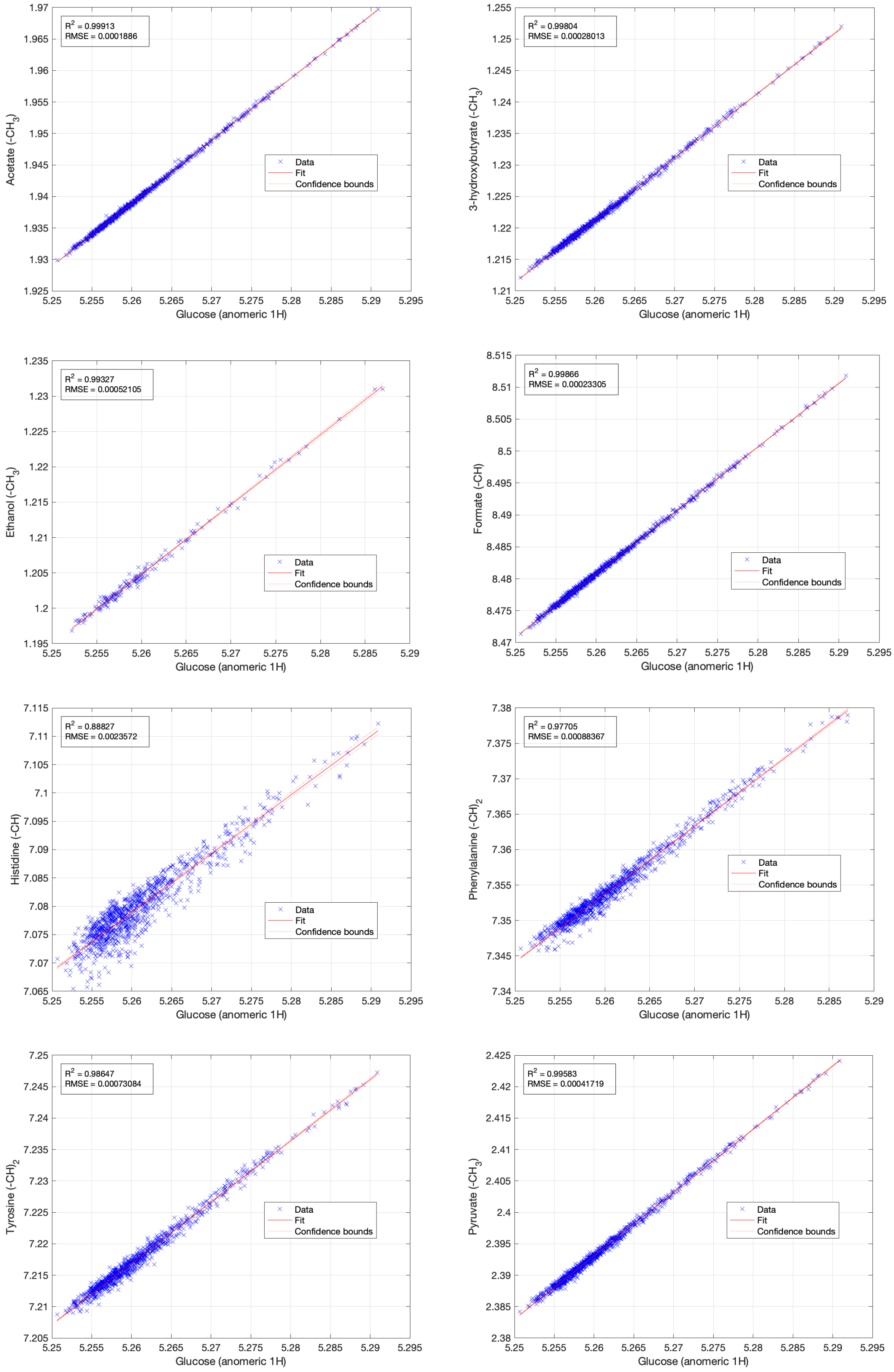

R² = 0.99913
RMSE = 0.0001886
Acetate (-CH3)
Glucose (anomeric 1H)
Data
Fit
Confidence bounds
R² = 0.99804
RMSE = 0.00028013
3-hydroxybutyrate (-CH3)
Glucose (anomeric 1H)
Data
Fit
Confidence bounds
R² = 0.99327
RMSE = 0.00052105
Ethanol (-CH3)
Glucose (anomeric 1H)
Data
Fit
Confidence bounds
R² = 0.99866
RMSE = 0.00023305
Formate (-CH)
Glucose (anomeric 1H)
Data
Fit
Confidence bounds
R² = 0.88827
RMSE = 0.0023572
Histidine (-CH)
Glucose (anomeric 1H)
Data
Fit
Confidence bounds
R² = 0.97705
RMSE = 0.00088367
Phenylalanine (-CH)2
Glucose (anomeric 1H)
Data
Fit
Confidence bounds
R² = 0.98647
RMSE = 0.00073084
Tyrosine (-CH)2
Glucose (anomeric 1H)
Data
Fit
Confidence bounds
R² = 0.99583
RMSE = 0.00041719
Pyruvate (-CH3)
Glucose (anomeric 1H)
Data
Fit
Confidence bounds

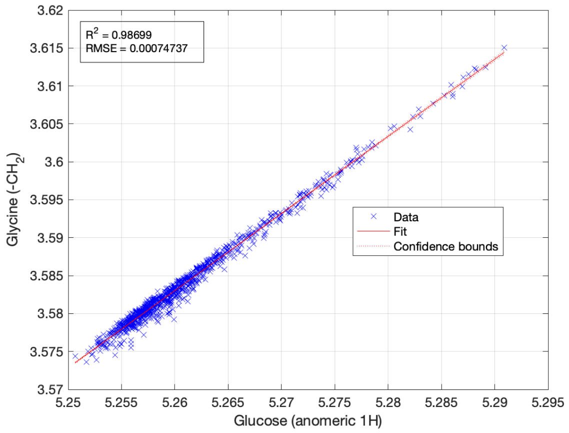

Figure S6. Scatter plots and fitted liner regression lines (y = a*x + b) for all spins systems with glucose anomeric proton δ as the predictor(x). For each fitted model, the calculated R^2 and RMSE values are depicted.

Acetone as predictor (x)

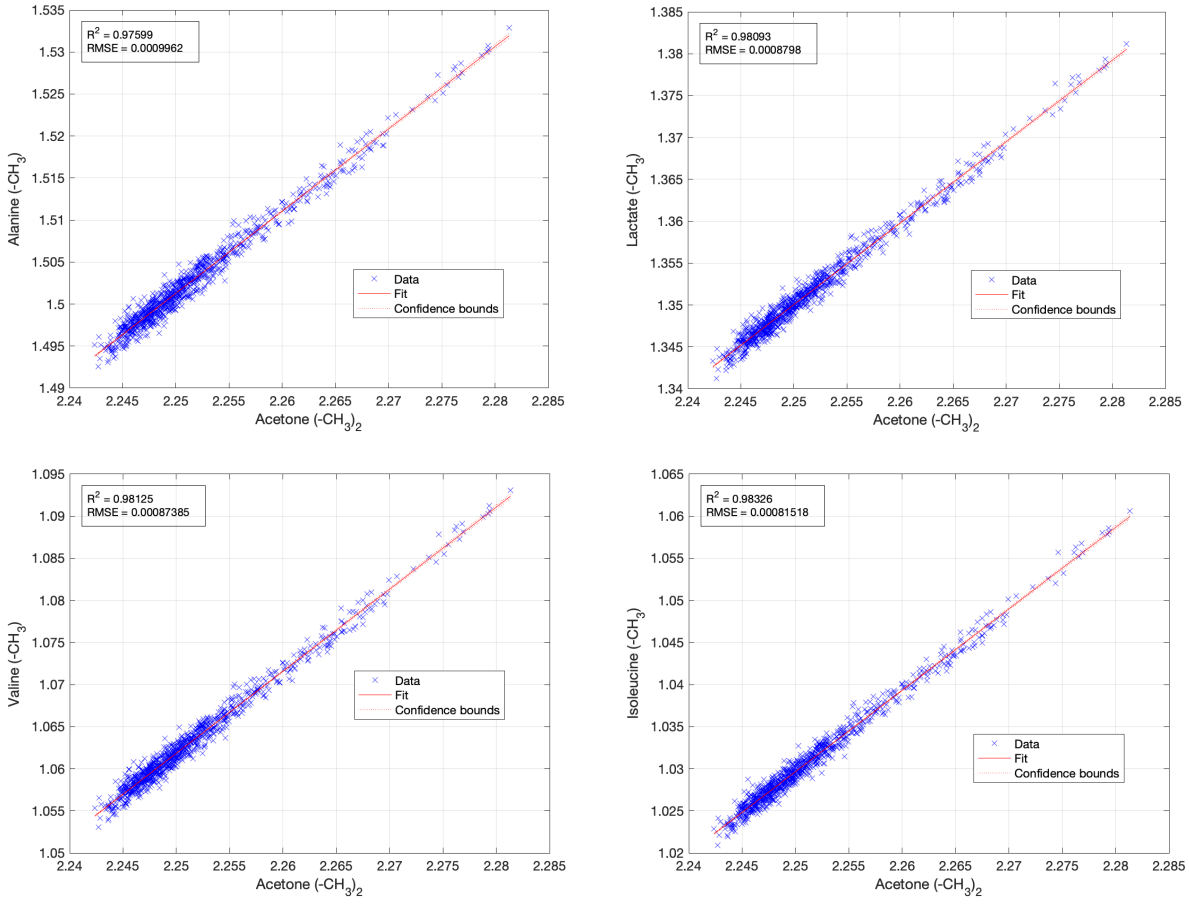

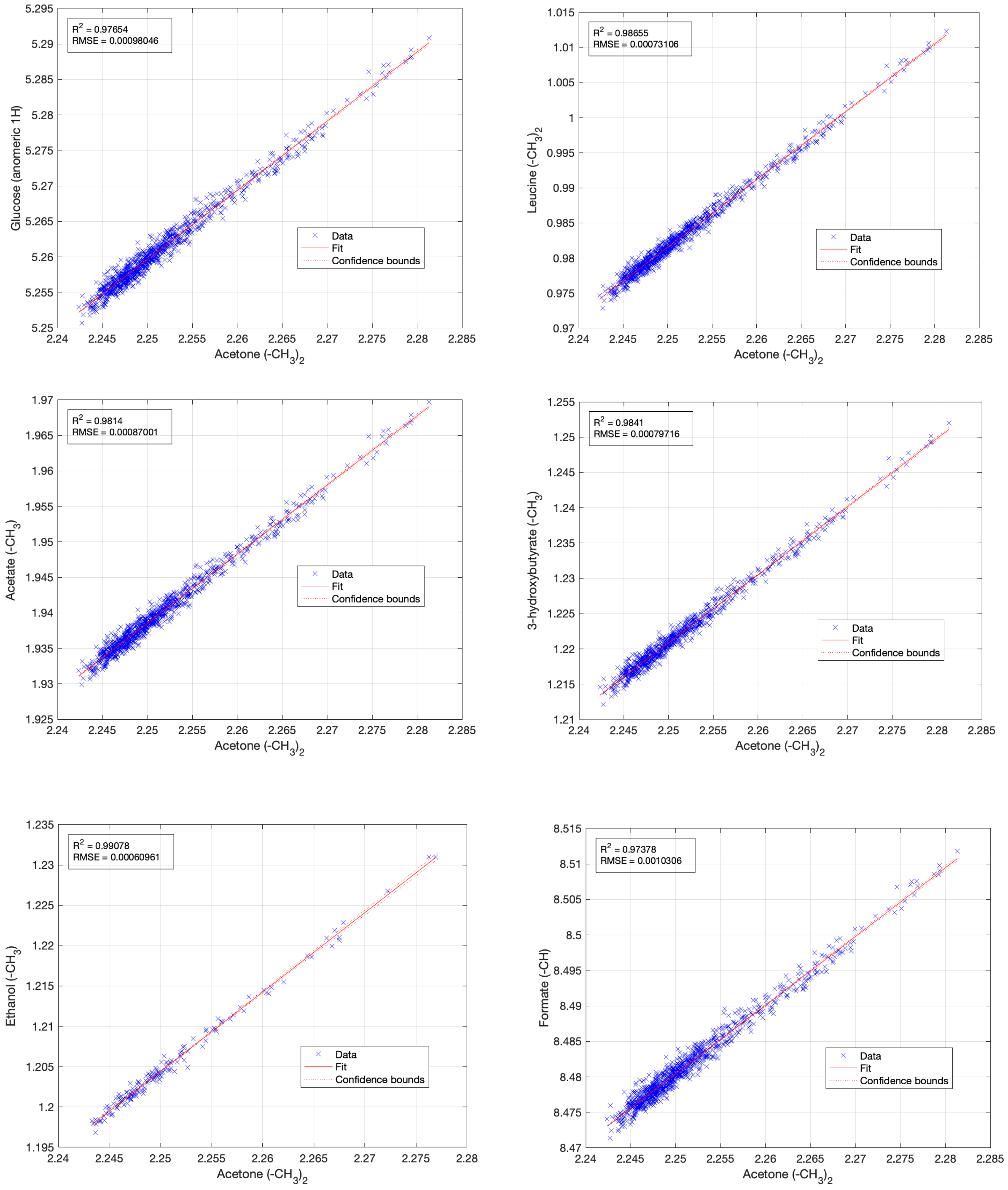
R2 = 0.97654
RMSE = 0.00098046
Glucose (anomeric 1H)
Acetone (-CH3)2
Data
Fit
Confidence bounds
R2 = 0.98655
RMSE = 0.00073106
Leucine (-CH3)2
Acetone (-CH3)2
R2 = 0.9814
RMSE = 0.00087001
Acetate (-CH3)
Acetone (-CH3)2
R2 = 0.9841
RMSE = 0.00079716
3-hydroxybutyrate (-CH3)
Acetone (-CH3)2
R2 = 0.99078
RMSE = 0.00060961
Ethanol (-CH3)
Acetone (-CH3)2
R2 = 0.97378
RMSE = 0.0010306
Formate (-CH)
Acetone (-CH3)2

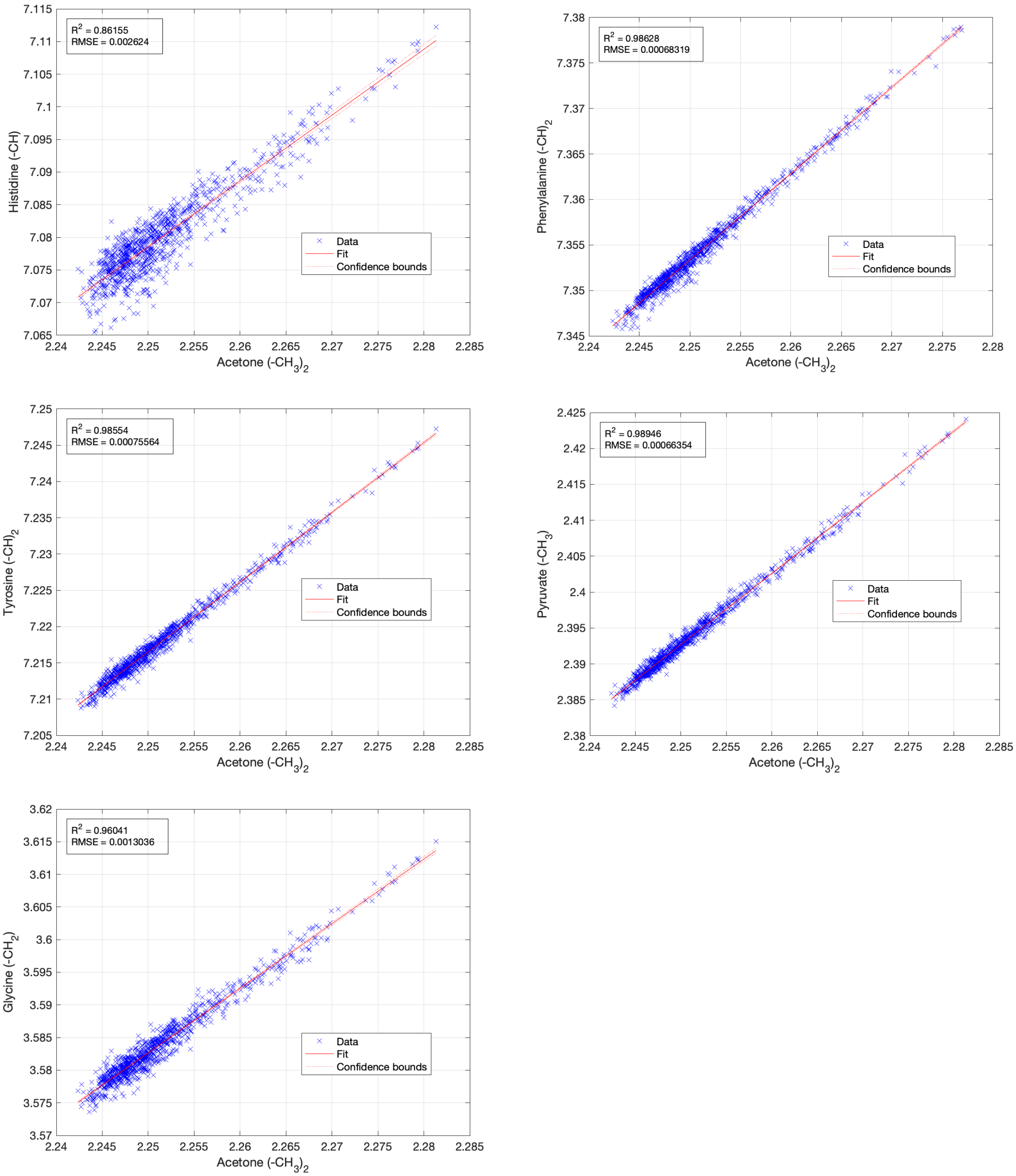

Figure S7. Scatter plots and fitted liner regression lines (y = a*x + b) for all spins systems with acetone $(-CH_3)_2$ δ as the predictor(x). For each fitted model, the calculated R^2 and RMSE values are depicted.

Leucine as predictor (x)

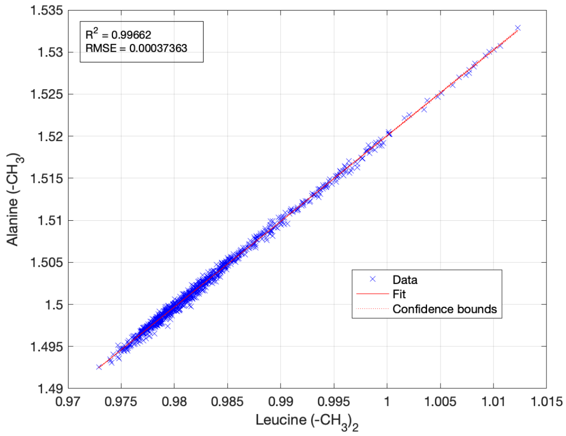

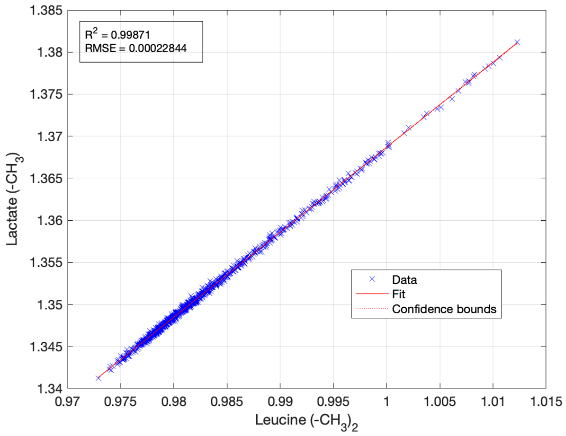

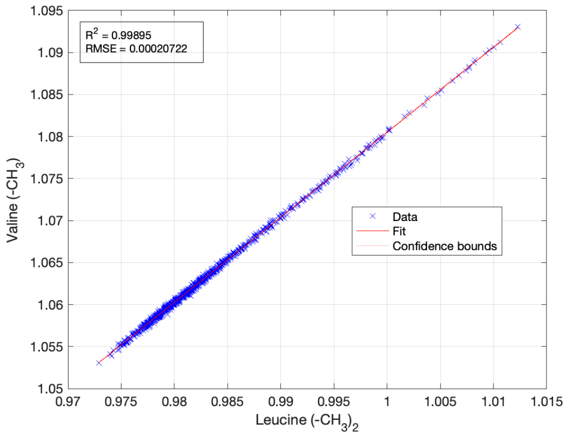

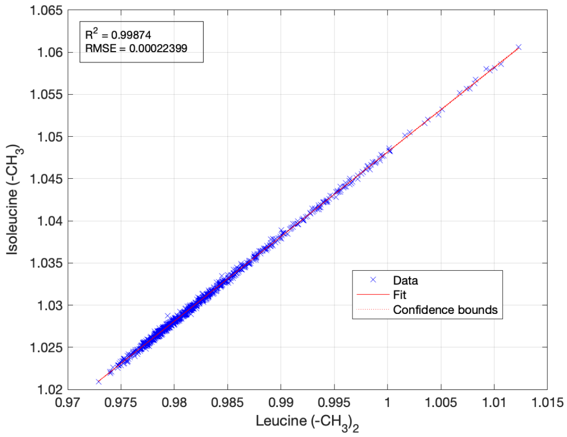

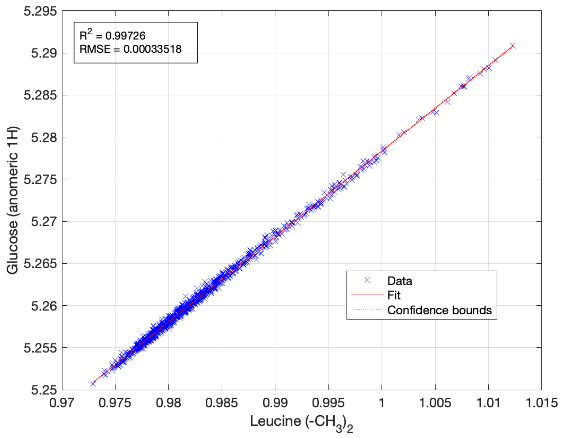

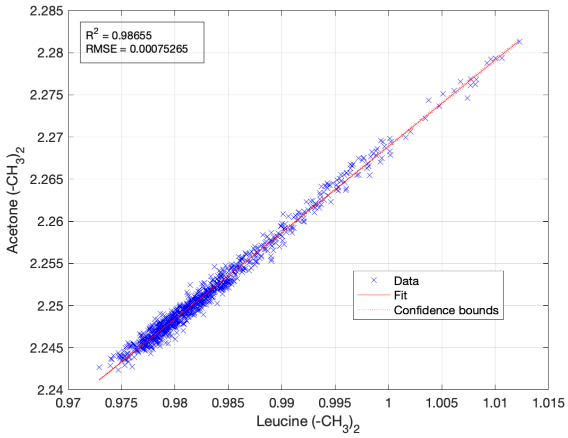

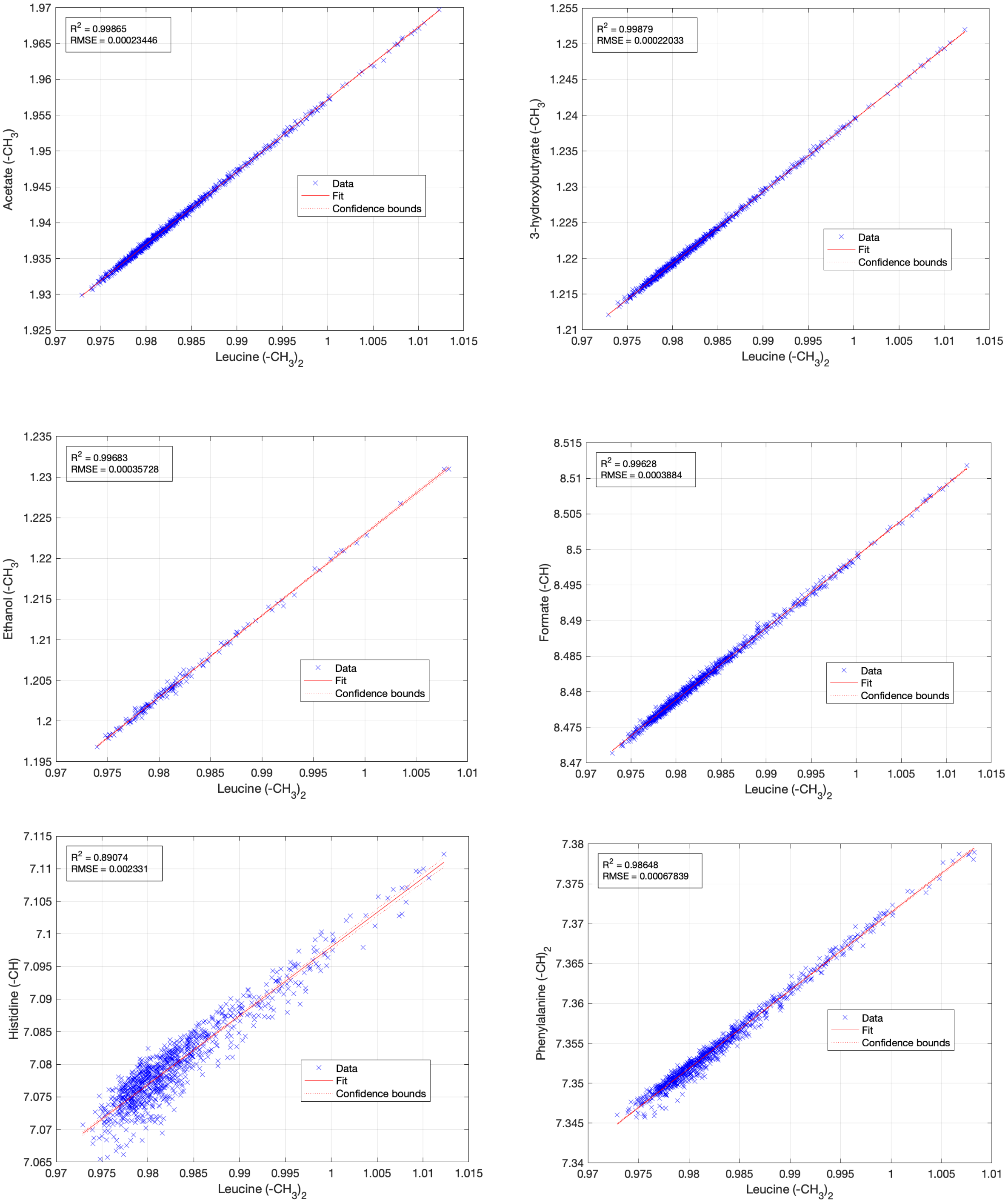
R2 = 0.99865
RMSE = 0.00023446
Data
Fit
Confidence bounds
Acetate (-CH3)
Leucine (-CH3)2
R2 = 0.99879
RMSE = 0.00022033
Data
Fit
Confidence bounds
3-hydroxybutyrate (-CH3)
Leucine (-CH3)2
R2 = 0.99683
RMSE = 0.00035728
Data
Fit
Confidence bounds
Ethanol (-CH3)
Leucine (-CH3)2
R2 = 0.99628
RMSE = 0.0003884
Data
Fit
Confidence bounds
Formate (-CH)
Leucine (-CH3)2
R2 = 0.89074
RMSE = 0.002331
Data
Fit
Confidence bounds
Histidine (-CH)
Leucine (-CH3)2
R2 = 0.98648
RMSE = 0.00067839
Data
Fit
Confidence bounds
Phenylalanine (-CH)2
Leucine (-CH3)2

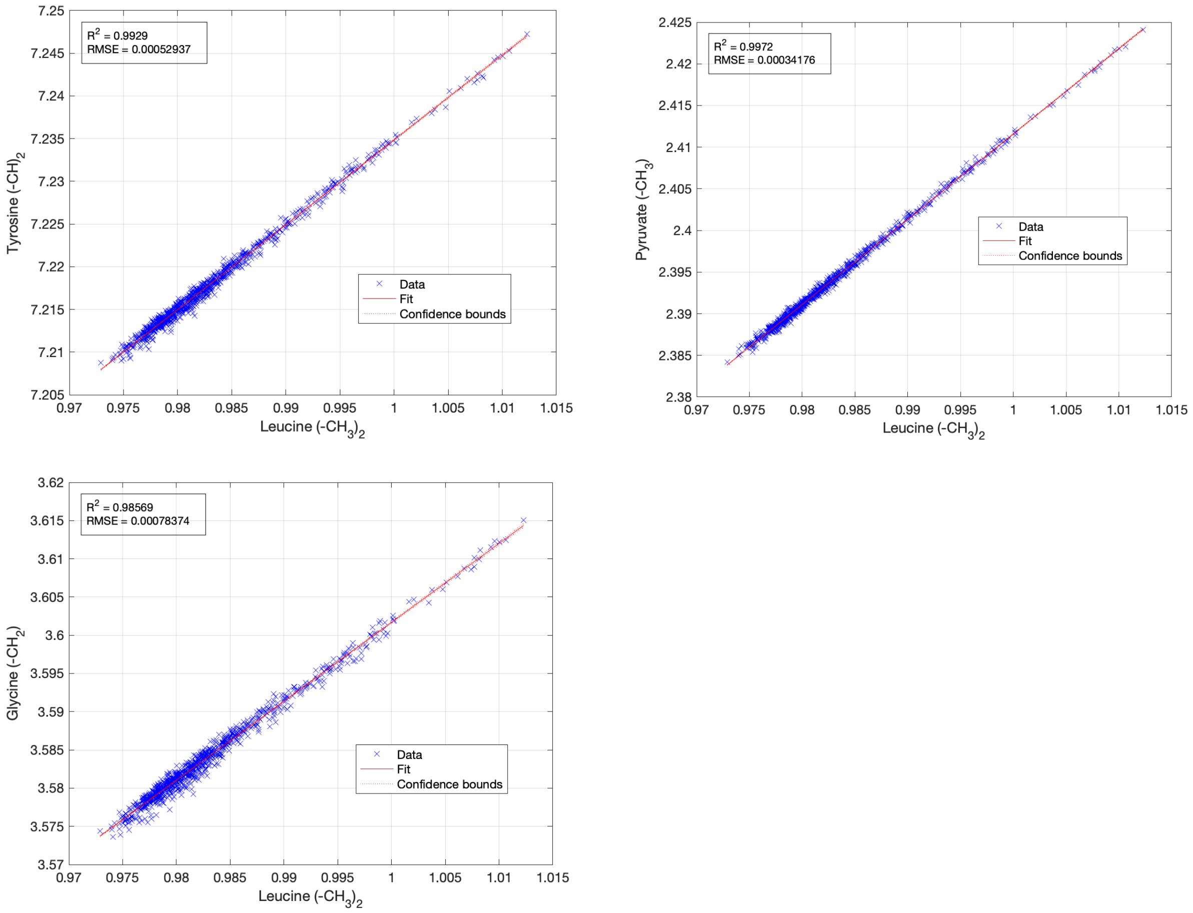

Figure S8. Scatter plots and fitted liner regression lines (y = a*x + b) for all spins systems with leucine $(-CH_3)_2$ δ as the predictor(x). For each fitted model, the calculated R^2 and RMSE values are depicted.

Acetate as predictor (x)

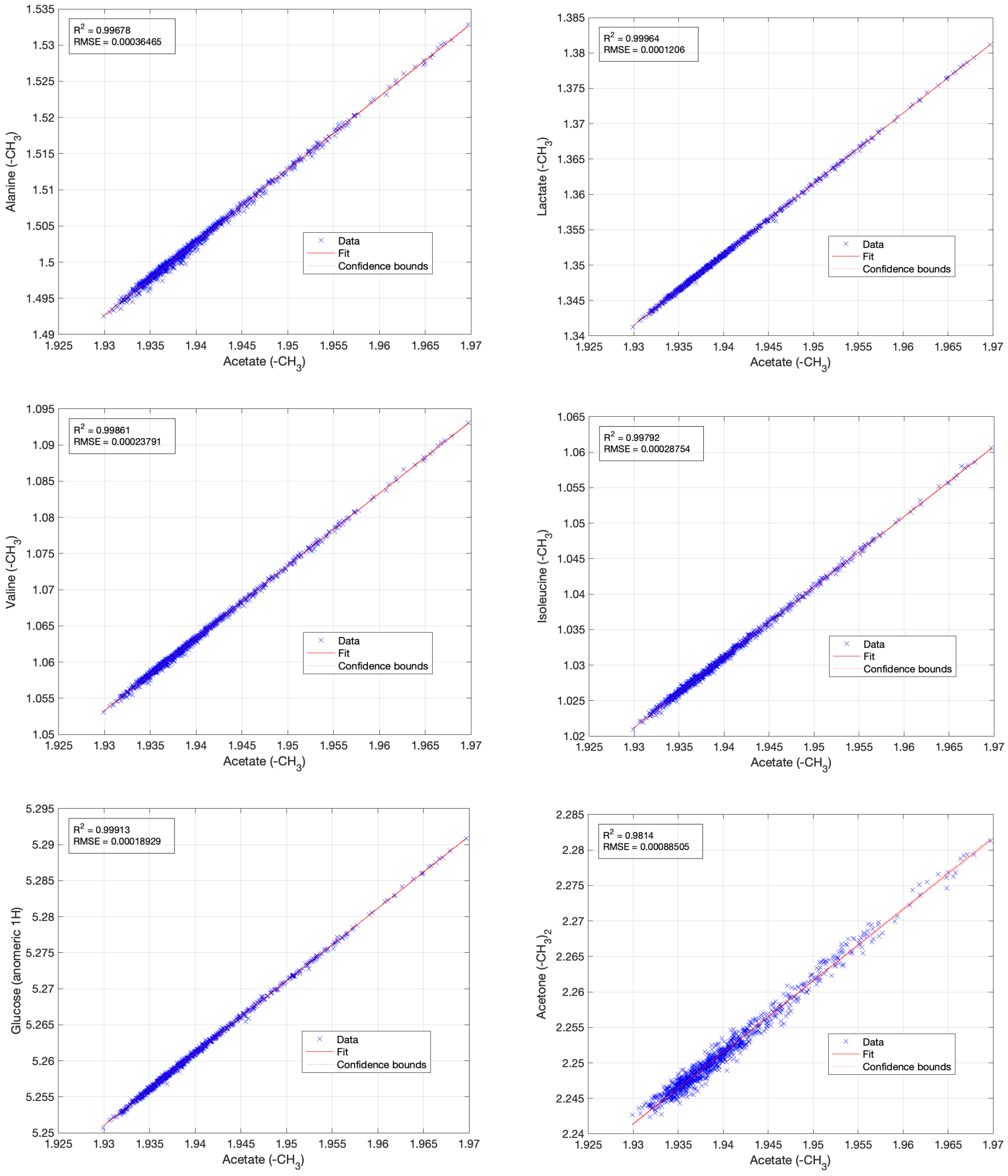

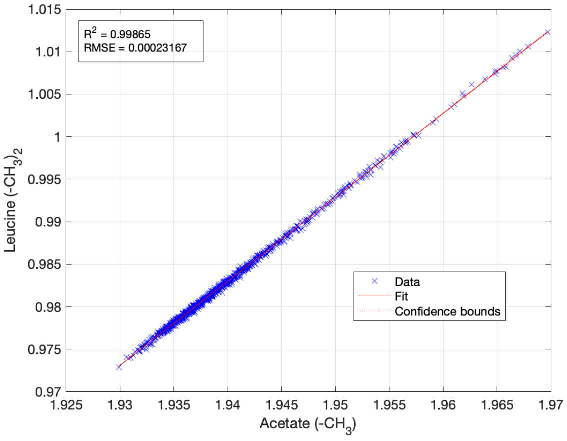
R^2 = 0.99865
RMSE = 0.00023167
Data
Fit
Confidence bounds
Leucine (-CH$_3$)$_2$
Acetate (-CH$_3$)

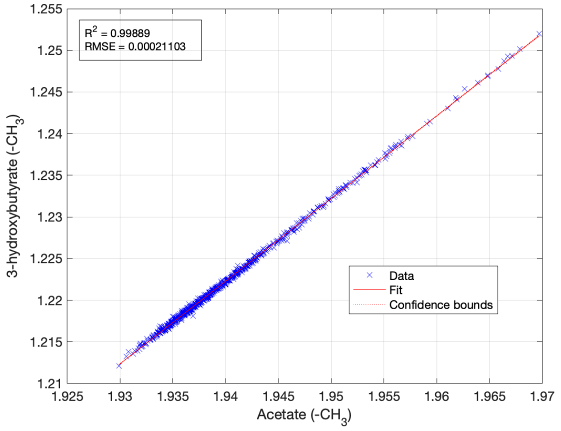
R^2 = 0.99889
RMSE = 0.00021103
Data
Fit
Confidence bounds
3-hydroxybutyrate (-CH$_3$)
Acetate (-CH$_3$)

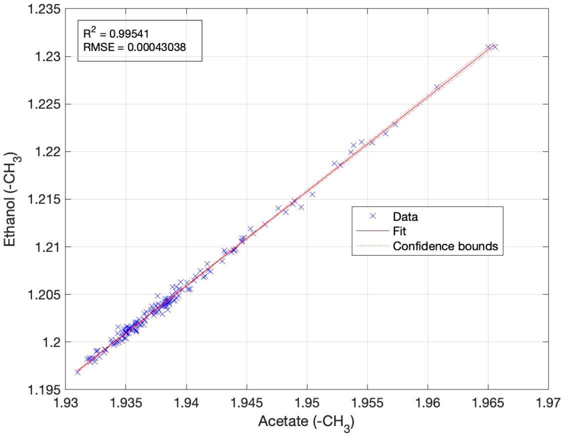
R^2 = 0.99541
RMSE = 0.00043038
Data
Fit
Confidence bounds
Ethanol (-CH$_3$)
Acetate (-CH$_3$)

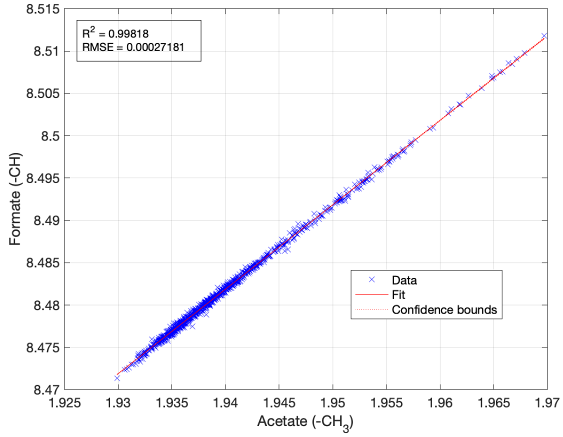
R^2 = 0.99818
RMSE = 0.00027181
Data
Fit
Confidence bounds
Formate (-CH)
Acetate (-CH$_3$)

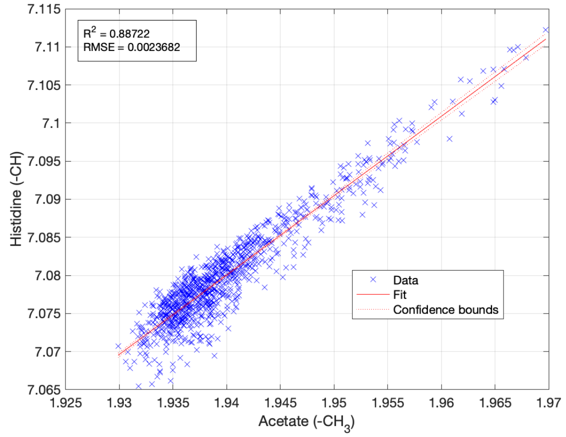
R^2 = 0.88722
RMSE = 0.0023682
Data
Fit
Confidence bounds
Histidine (-CH)
Acetate (-CH$_3$)

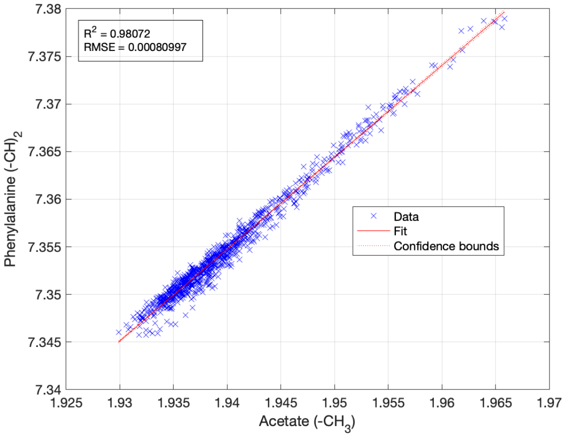
R^2 = 0.98072
RMSE = 0.00080997
Data
Fit
Confidence bounds
Phenylalanine (-CH)$_2$
Acetate (-CH$_3$)

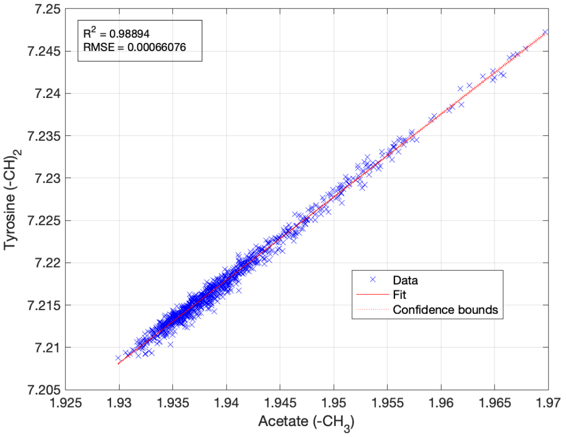
R^2 = 0.98894
RMSE = 0.00066076
Data
Fit
Confidence bounds
Tyrosine (-CH)$_2$
Acetate (-CH$_3$)

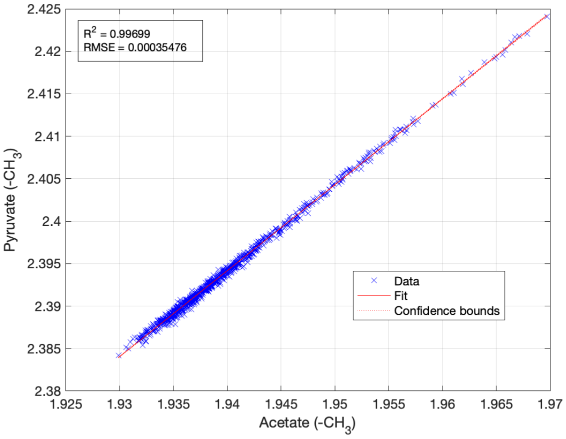
R^2 = 0.99699
RMSE = 0.00035476
Data
Fit
Confidence bounds
Pyruvate (-CH$_3$)
Acetate (-CH$_3$)

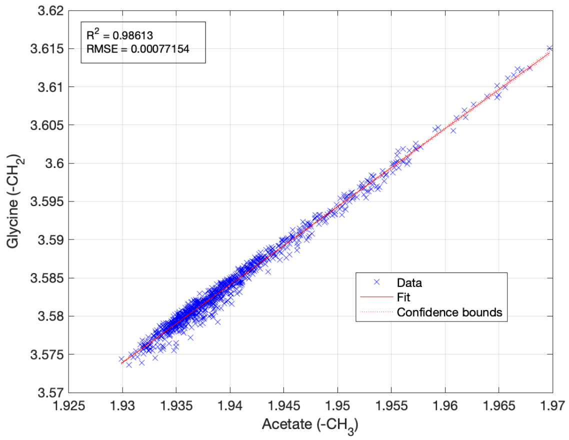

Figure S9. Scatter plots and fitted liner regression lines (y = a*x + b) for all spins systems with acetate $-CH_3$ δ as the predictor(x). For each fitted model, the calculated R^2 and RMSE values are depicted.

3-hydroxybutyrate as predictor (x)

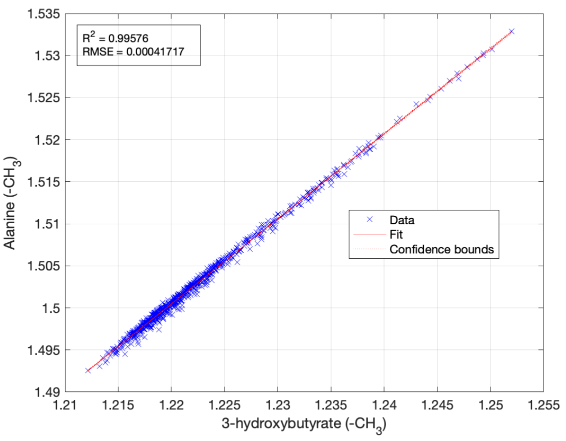

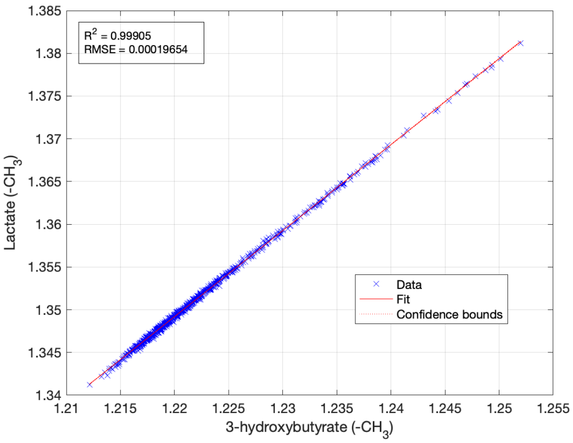

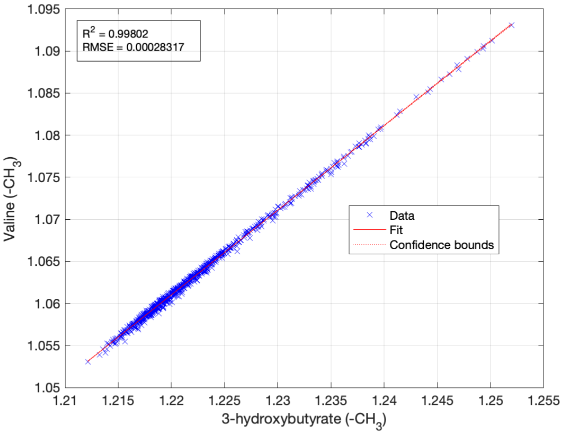

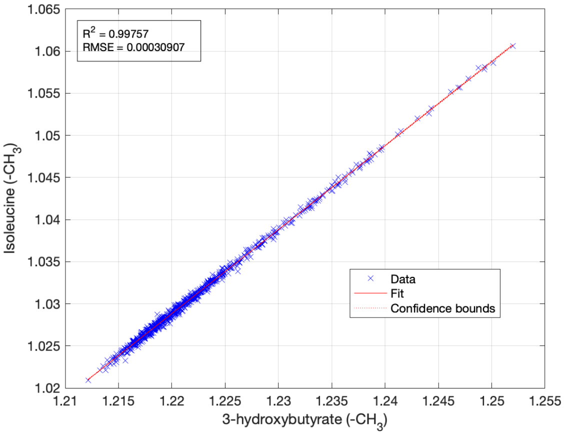

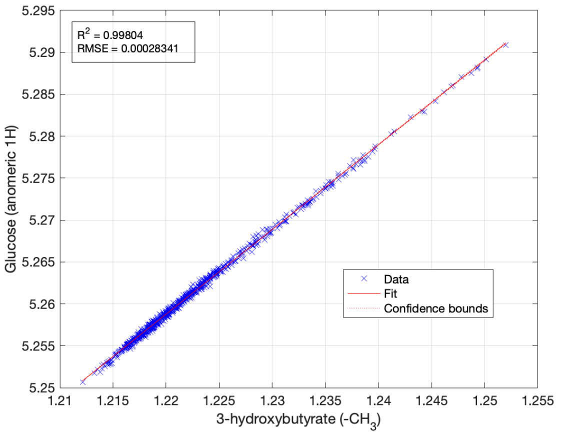

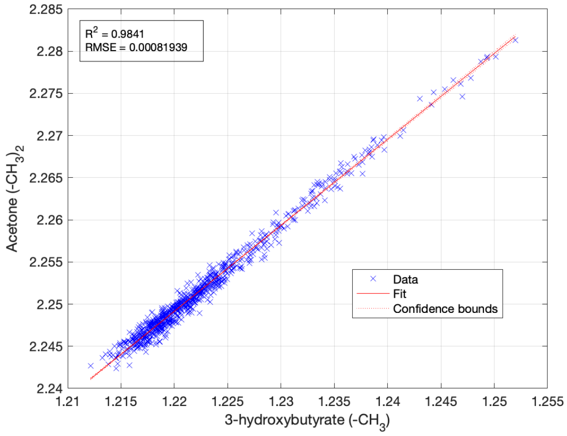

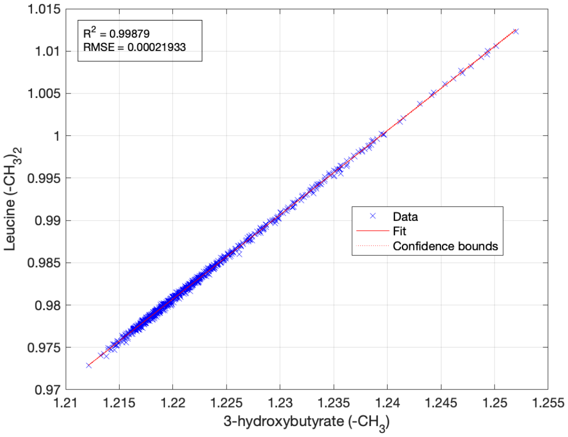
R^2 = 0.99879
RMSE = 0.00021933
Data
Fit
Confidence bounds
Leucine (-CH$_3$)$_2$
3-hydroxybutyrate (-CH$_3$)

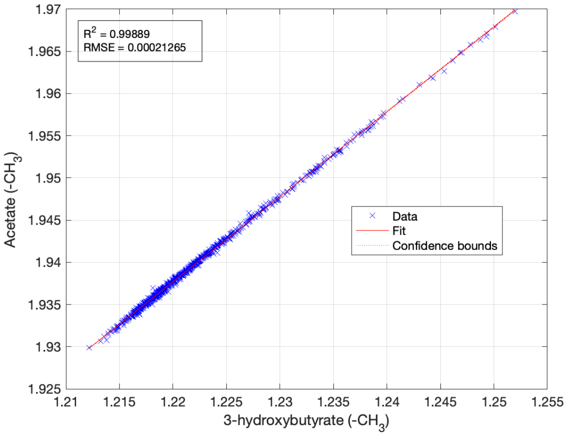
R^2 = 0.99889
RMSE = 0.00021265
Data
Fit
Confidence bounds
Acetate (-CH$_3$)
3-hydroxybutyrate (-CH$_3$)

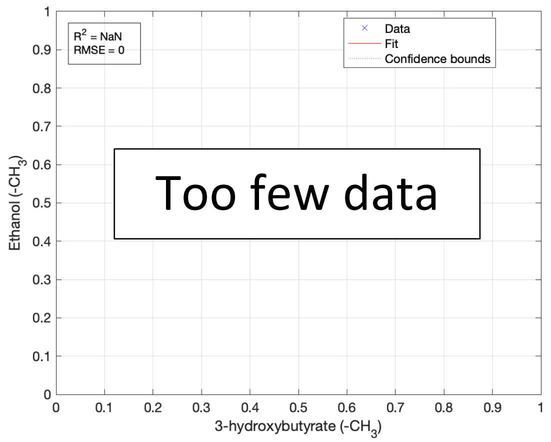
R^2 = NaN
RMSE = 0
Data
Fit
Confidence bounds
Too few data
Ethanol (-CH$_3$)
3-hydroxybutyrate (-CH$_3$)

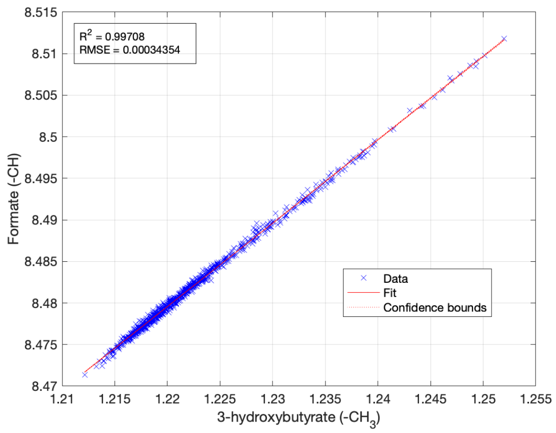
R^2 = 0.99708
RMSE = 0.00034354
Data
Fit
Confidence bounds
Formate (-CH)
3-hydroxybutyrate (-CH$_3$)

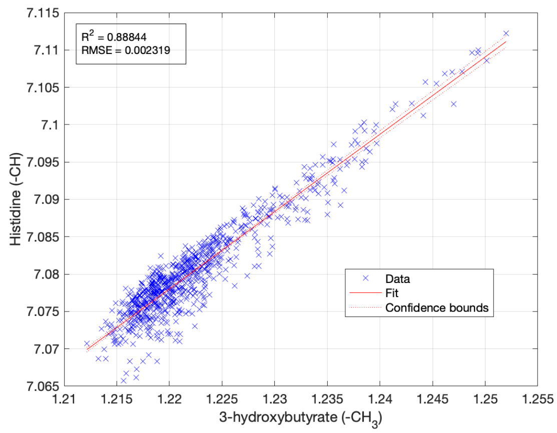
R^2 = 0.88844
RMSE = 0.002319
Data
Fit
Confidence bounds
Histidine (-CH)
3-hydroxybutyrate (-CH$_3$)

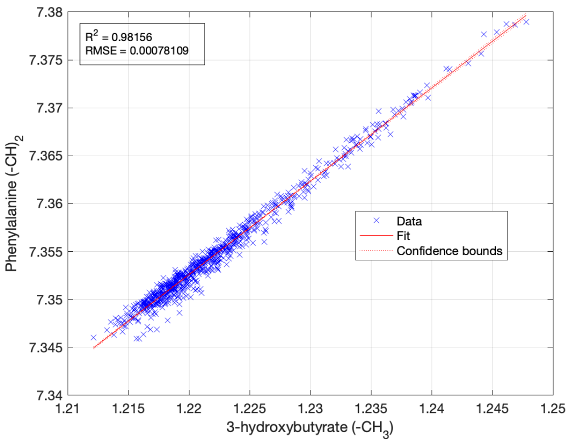
R^2 = 0.98156
RMSE = 0.00078109
Data
Fit
Confidence bounds
Phenylalanine (-CH)$_2$
3-hydroxybutyrate (-CH$_3$)

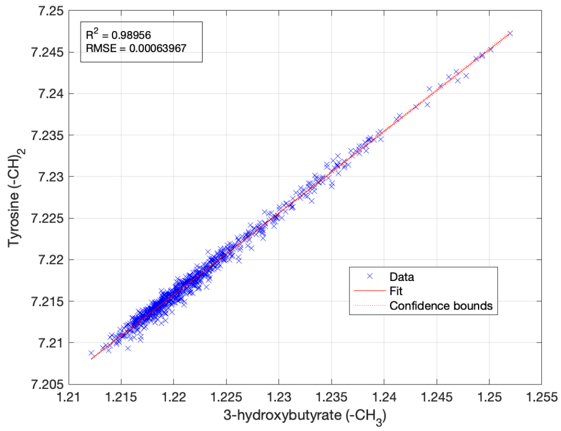
R^2 = 0.98956
RMSE = 0.00063967
Data
Fit
Confidence bounds
Tyrosine (-CH)$_2$
3-hydroxybutyrate (-CH$_3$)

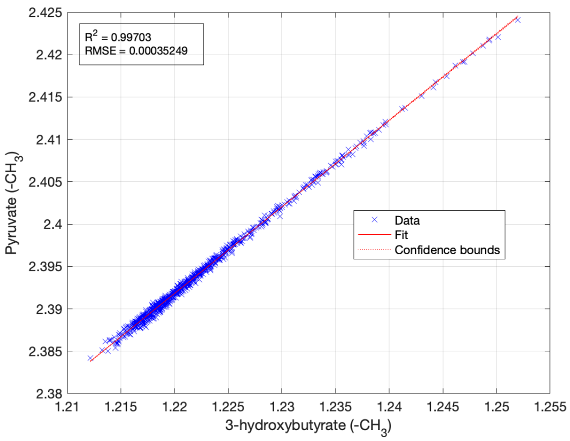
R^2 = 0.99703
RMSE = 0.00035249
Data
Fit
Confidence bounds
Pyruvate (-CH$_3$)
3-hydroxybutyrate (-CH$_3$)

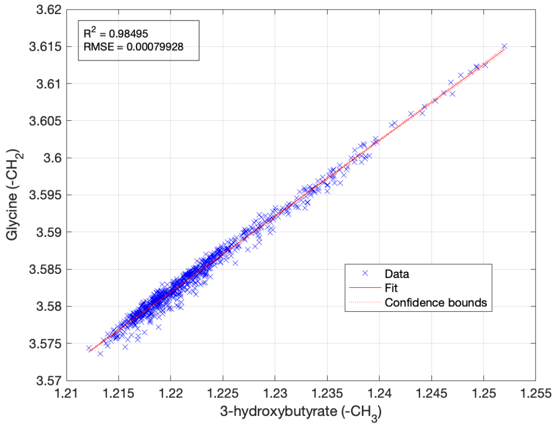

Figure S10. Scatter plots and fitted liner regression lines (y = a*x + b) for all spins systems with 3-hydroxybutyrate $-CH_3$ δ as the predictor(x). For each fitted model, the calculated R^2 and RMSE values are depicted.

Ethanol as predictor (x)

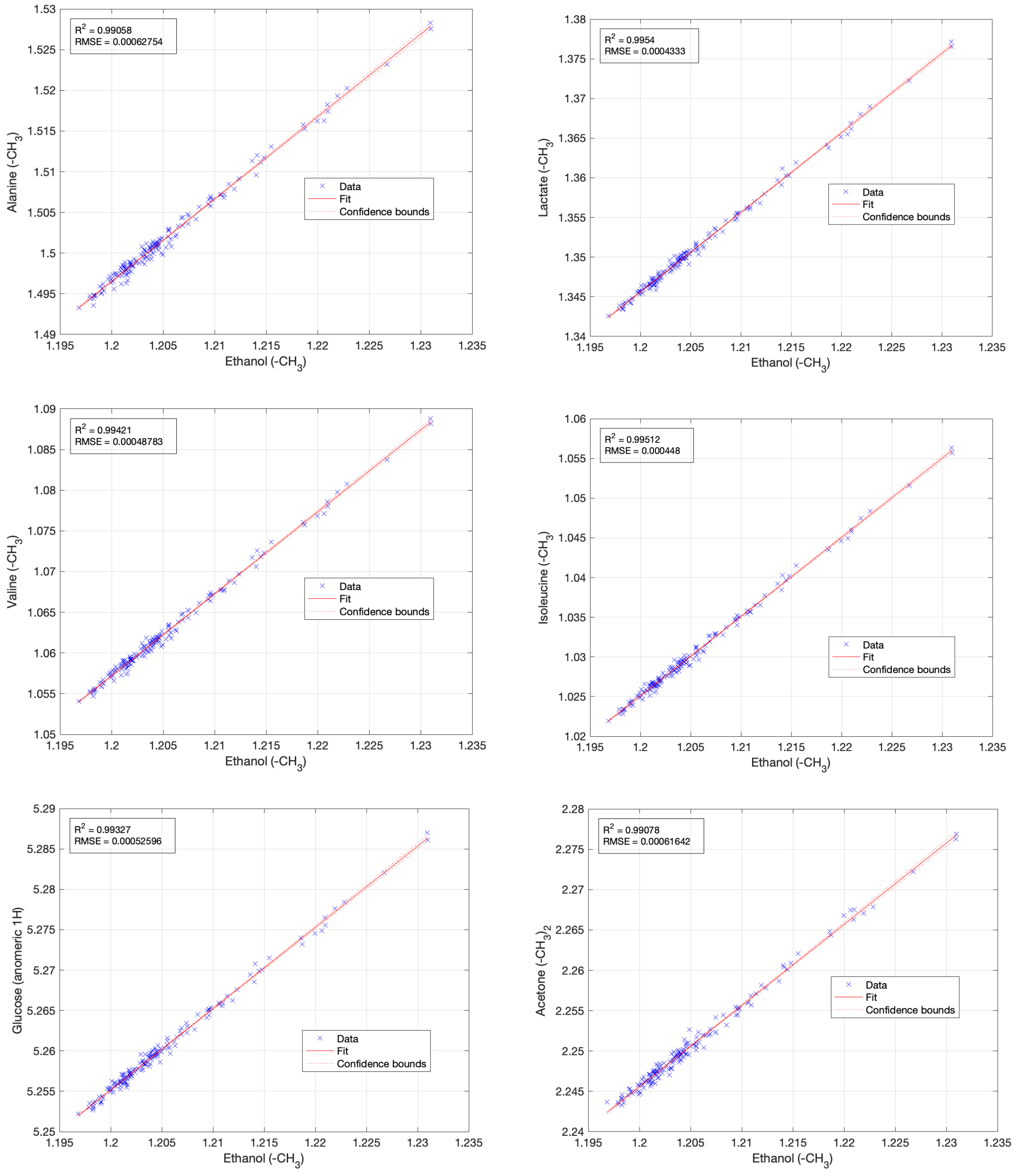

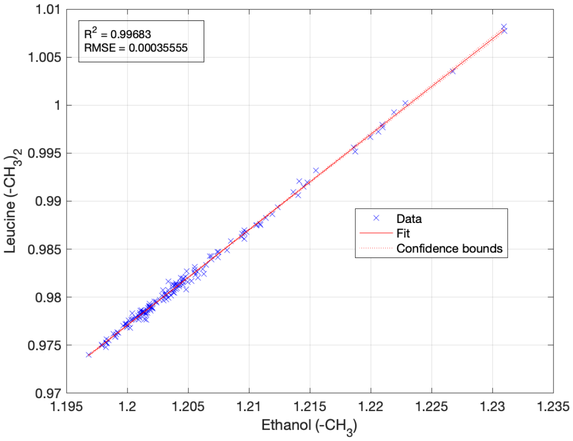

R² = 0.99683
RMSE = 0.00035555
Data
Fit
Confidence bounds
Leucine (-CH3)2
Ethanol (-CH3)

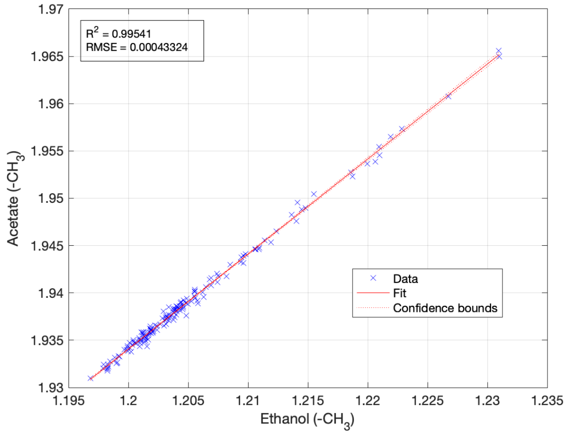

R² = 0.99541
RMSE = 0.00043324
Data
Fit
Confidence bounds
Acetate (-CH3)
Ethanol (-CH3)

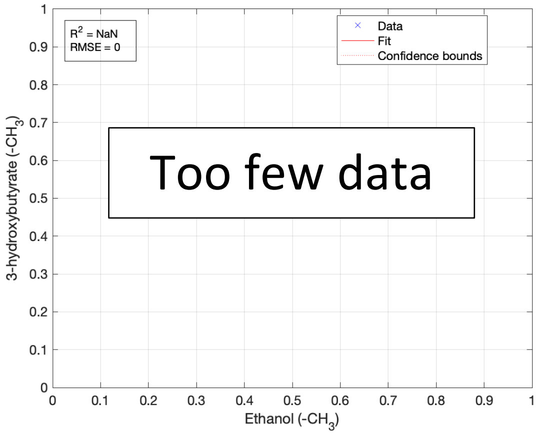

R² = NaN
RMSE = 0
Data
Fit
Confidence bounds
Too few data
3-hydroxybutyrate (-CH3)
Ethanol (-CH3)

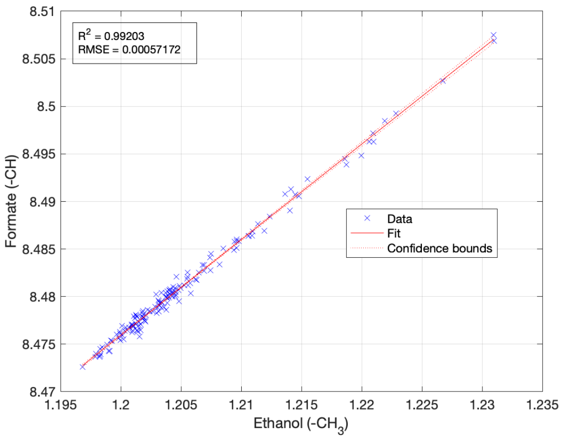

R² = 0.99203
RMSE = 0.00057172
Data
Fit
Confidence bounds
Formate (-CH)
Ethanol (-CH3)

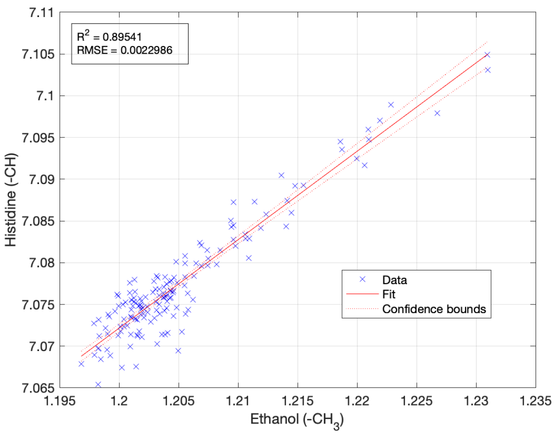

R² = 0.89541
RMSE = 0.0022986
Data
Fit
Confidence bounds
Histidine (-CH)
Ethanol (-CH3)

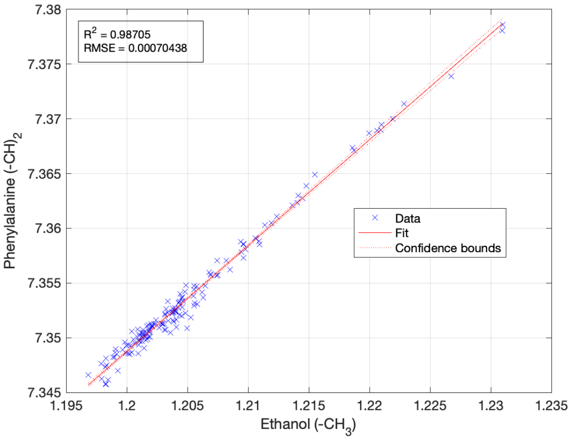

R² = 0.98705
RMSE = 0.00070438
Data
Fit
Confidence bounds
Phenylalanine (-CH)2
Ethanol (-CH3)

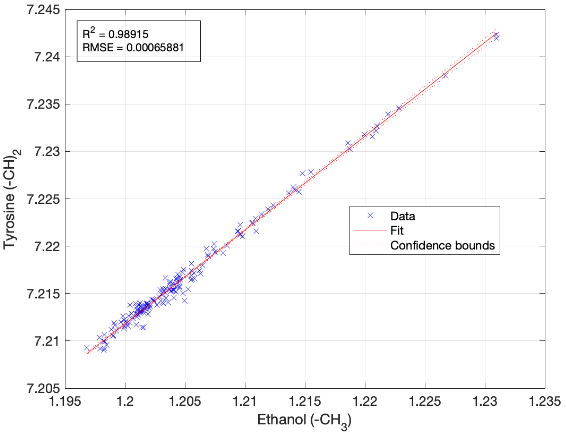

R² = 0.98915
RMSE = 0.00065881
Data
Fit
Confidence bounds
Tyrosine (-CH)2
Ethanol (-CH3)

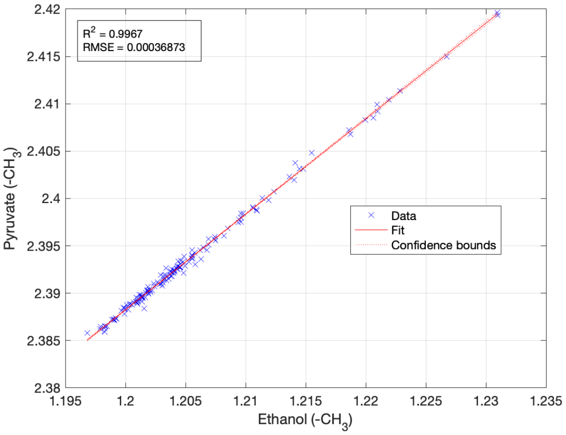

R² = 0.9967
RMSE = 0.00036873
Data
Fit
Confidence bounds
Pyruvate (-CH3)
Ethanol (-CH3)

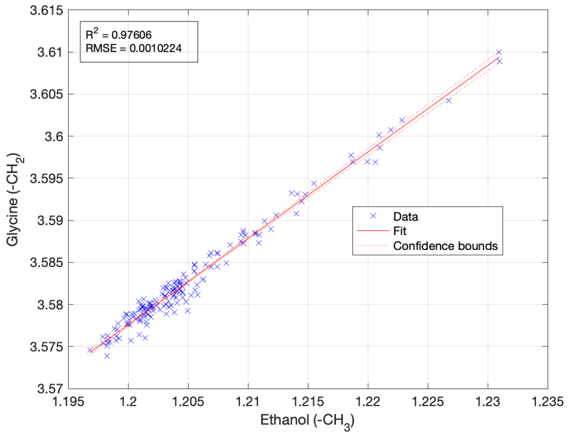

Figure S11. Scatter plots and fitted liner regression lines (y = a*x + b) for all spins systems with ethanol -CH_3 δ as the predictor(x). For each fitted model, the calculated R^2 and RMSE values are depicted.

Formate as predictor (x)

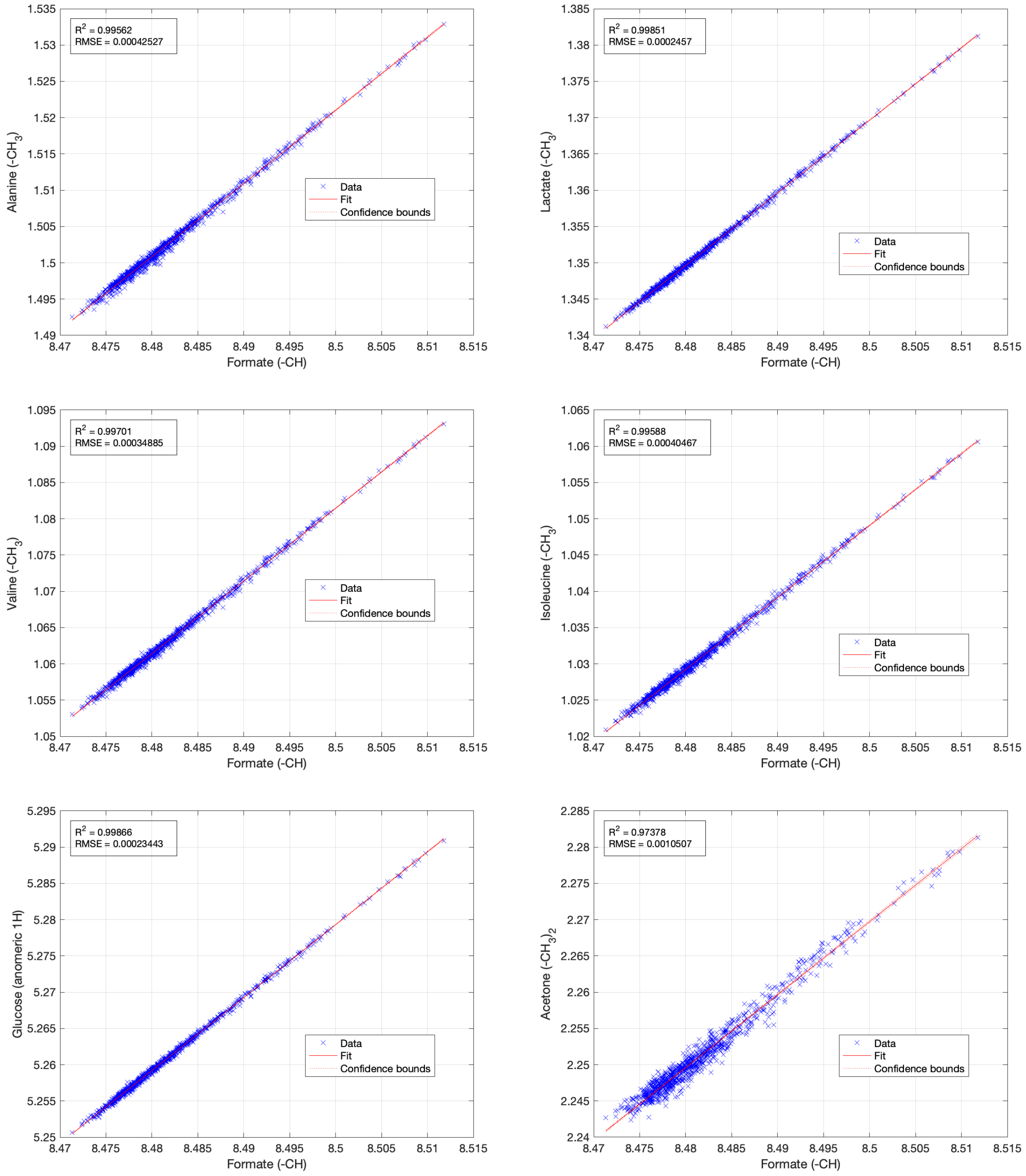

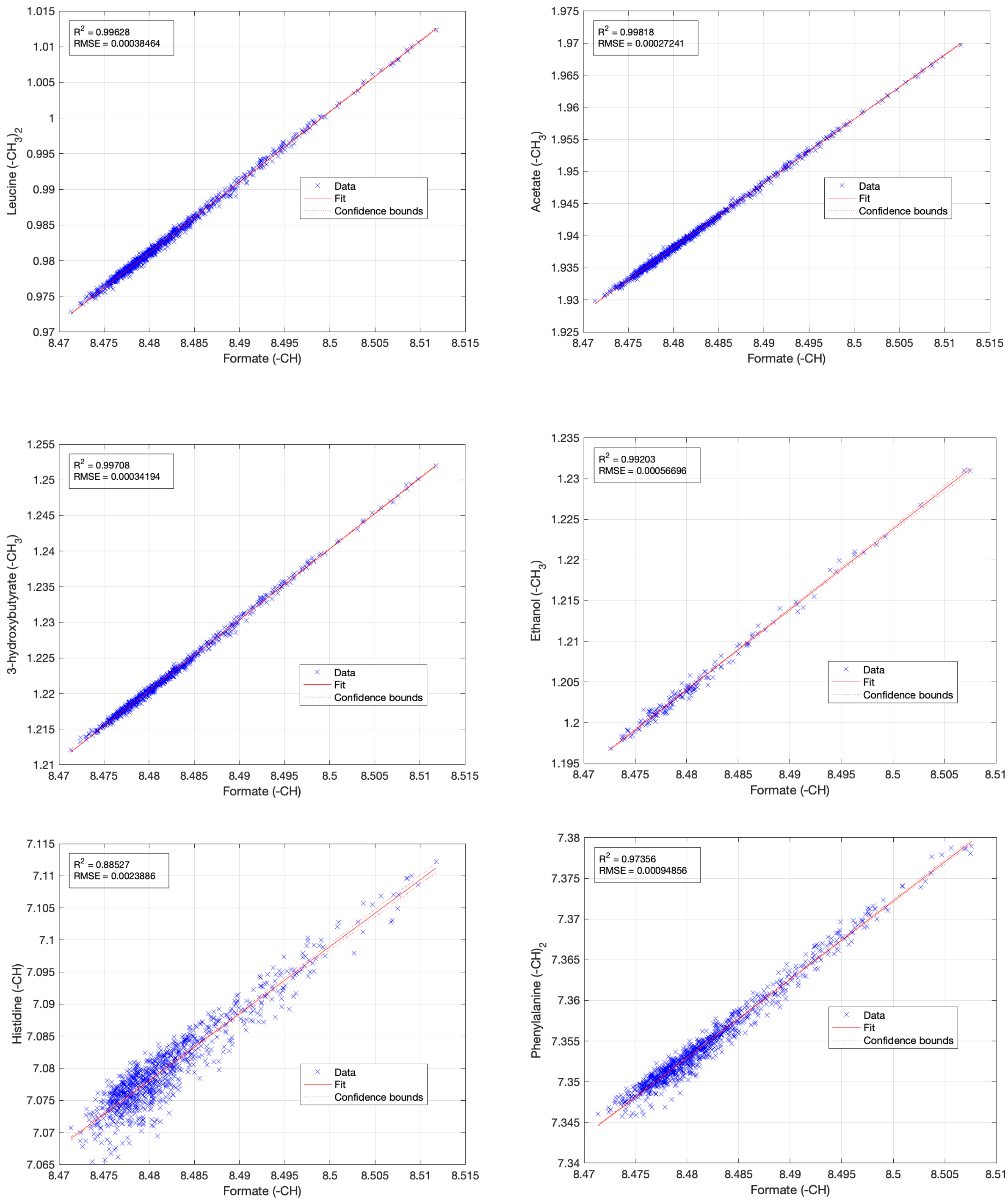
R2 = 0.99628
RMSE = 0.00038464
Leucine (-CH3)2
Formate (-CH)
Data
Fit
Confidence bounds
R2 = 0.99818
RMSE = 0.00027241
Acetate (-CH3)
Formate (-CH)
Data
Fit
Confidence bounds
R2 = 0.99708
RMSE = 0.00034194
3-hydroxybutyrate (-CH3)
Formate (-CH)
Data
Fit
Confidence bounds
R2 = 0.99203
RMSE = 0.00056696
Ethanol (-CH3)
Formate (-CH)
Data
Fit
Confidence bounds
R2 = 0.88527
RMSE = 0.0023886
Histidine (-CH)
Formate (-CH)
Data
Fit
Confidence bounds
R2 = 0.97356
RMSE = 0.00094856
Phenylalanine (-CH)2
Formate (-CH)
Data
Fit
Confidence bounds

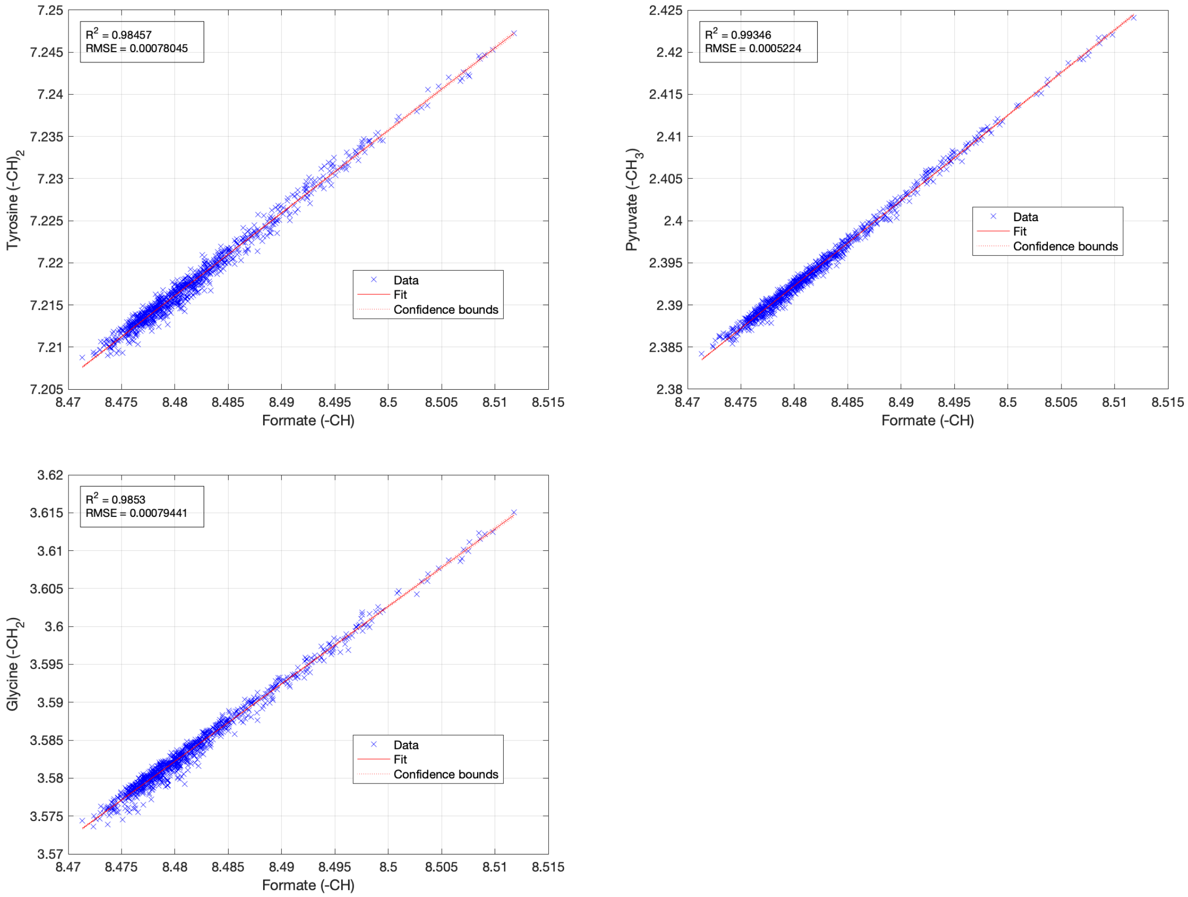

Figure S12. Scatter plots and fitted liner regression lines (y = a*x + b) for all spins systems with formate -CH δ as the predictor(x). For each fitted model, the calculated R^2 and RMSE values are depicted.

Histidine as predictor (x)

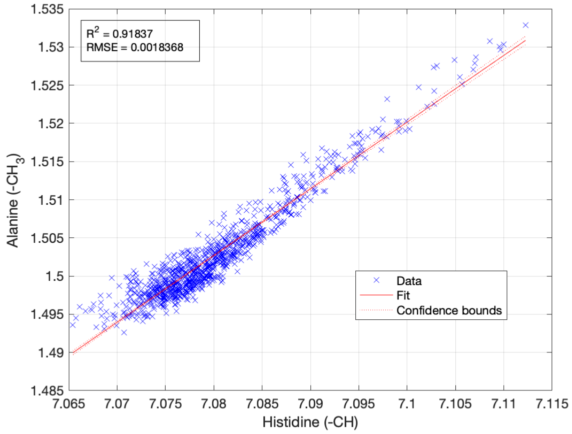

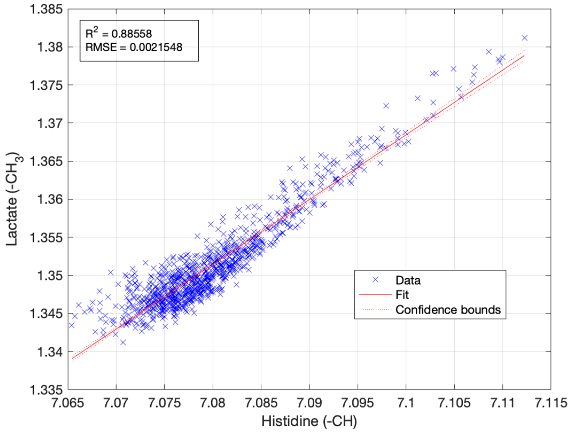

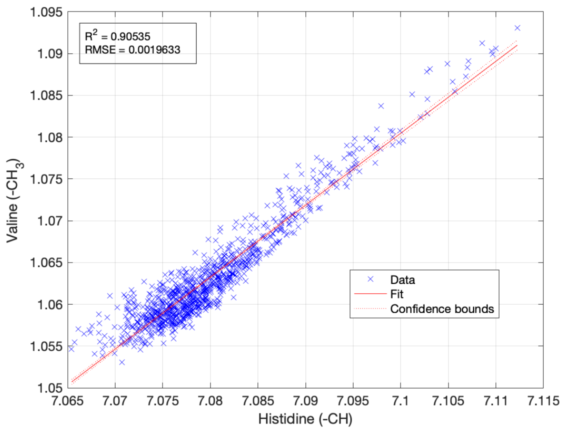

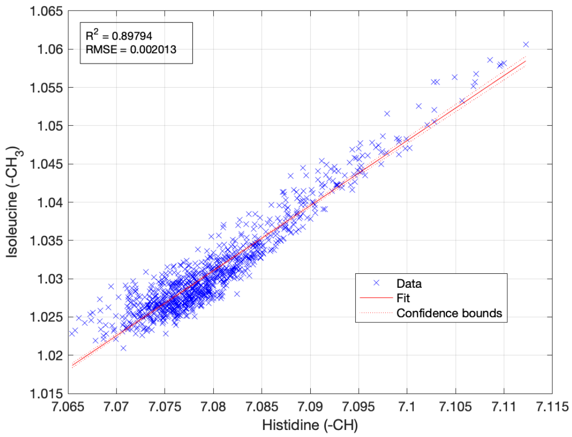

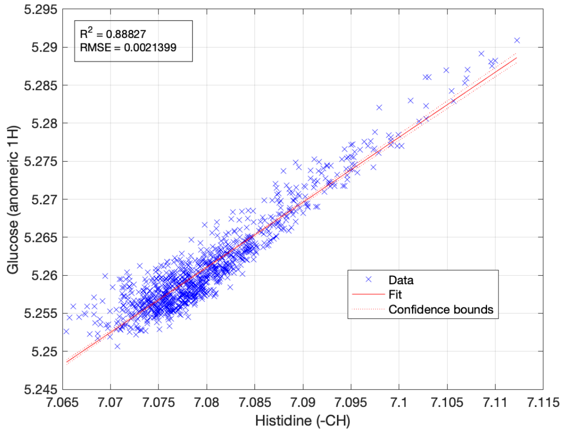

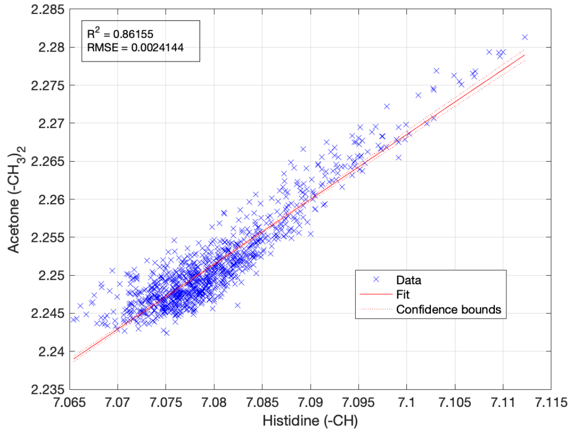

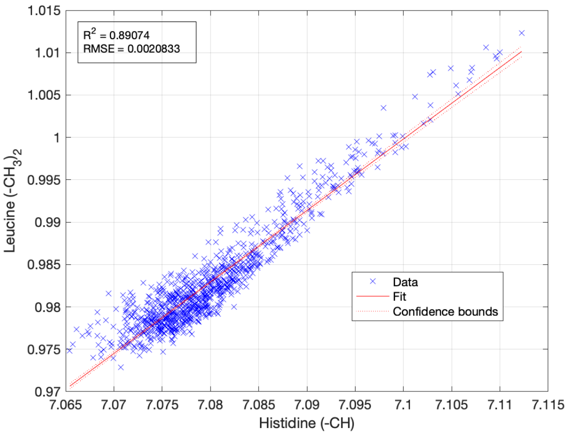
R2 = 0.89074
RMSE = 0.0020833
Data
Fit
Confidence bounds
Leucine (-CH3)2
Histidine (-CH)

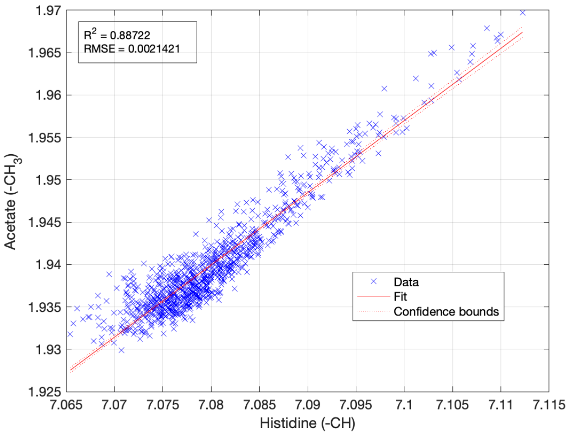
R2 = 0.88722
RMSE = 0.0021421
Data
Fit
Confidence bounds
Acetate (-CH3)
Histidine (-CH)

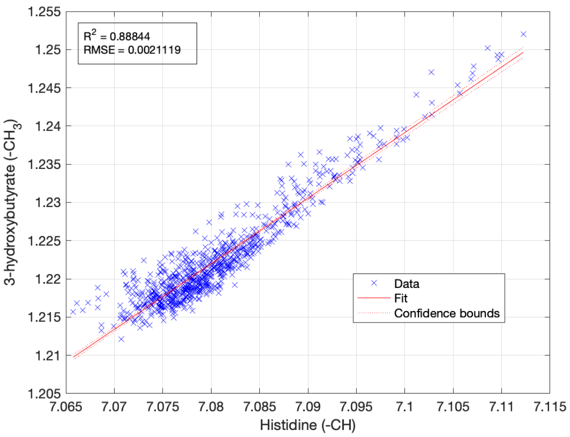
R2 = 0.88844
RMSE = 0.0021119
Data
Fit
Confidence bounds
3-hydroxybutyrate (-CH3)
Histidine (-CH)

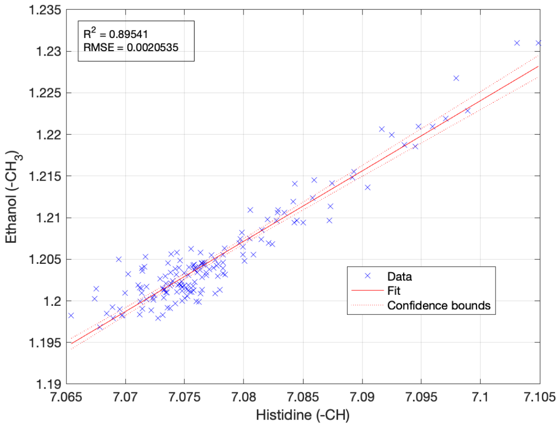
R2 = 0.89541
RMSE = 0.0020535
Data
Fit
Confidence bounds
Ethanol (-CH3)
Histidine (-CH)

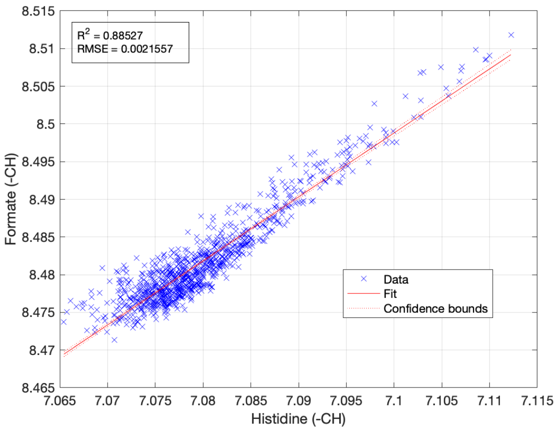
R2 = 0.88527
RMSE = 0.0021557
Data
Fit
Confidence bounds
Formate (-CH)
Histidine (-CH)

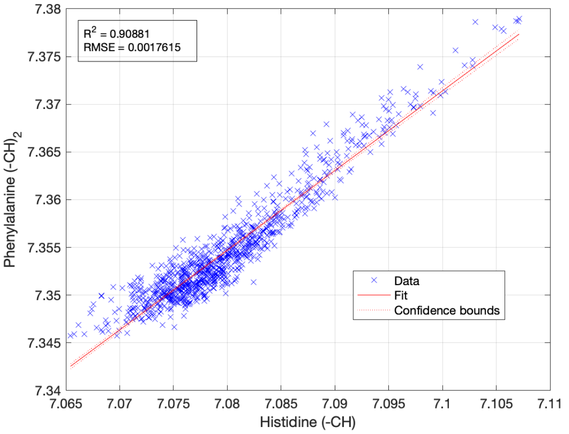
R2 = 0.90881
RMSE = 0.0017615
Data
Fit
Confidence bounds
Phenylalanine (-CH)2
Histidine (-CH)

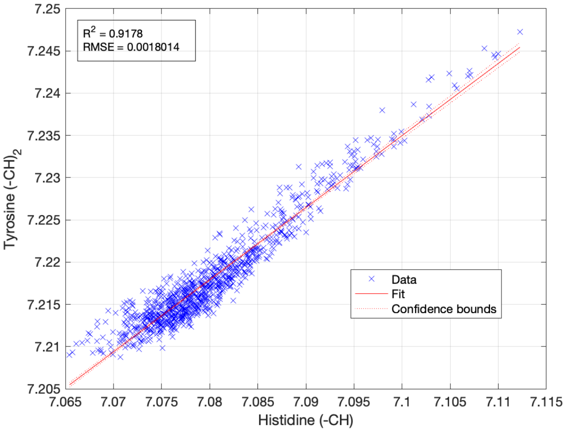
R2 = 0.9178
RMSE = 0.0018014
Data
Fit
Confidence bounds
Tyrosine (-CH)2
Histidine (-CH)

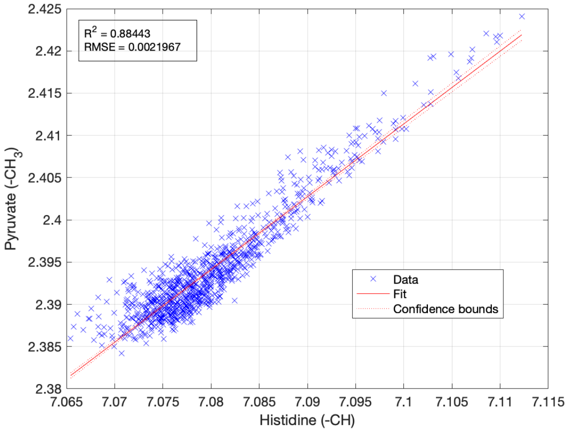
R2 = 0.88443
RMSE = 0.0021967
Data
Fit
Confidence bounds
Pyruvate (-CH3)
Histidine (-CH)

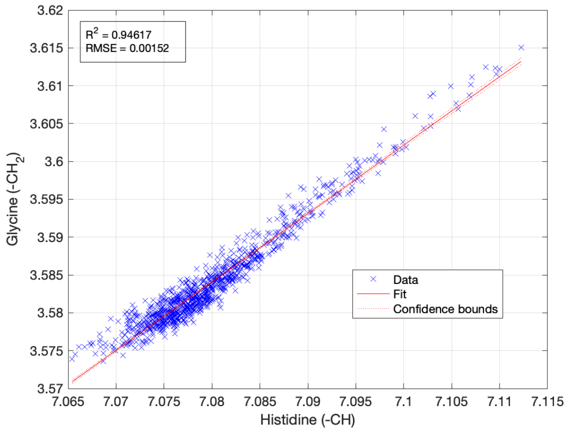

Figure S13. Scatter plots and fitted liner regression lines (y = a*x + b) for all spins systems with histidine -CH δ as the predictor(x). For each fitted model, the calculated R^2 and RMSE values are depicted.

Phenylalanine as predictor (x)

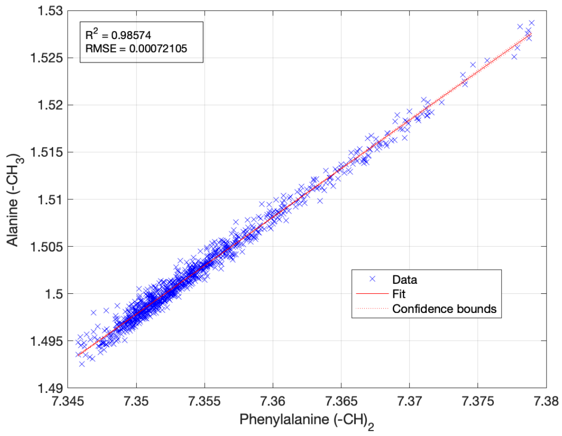

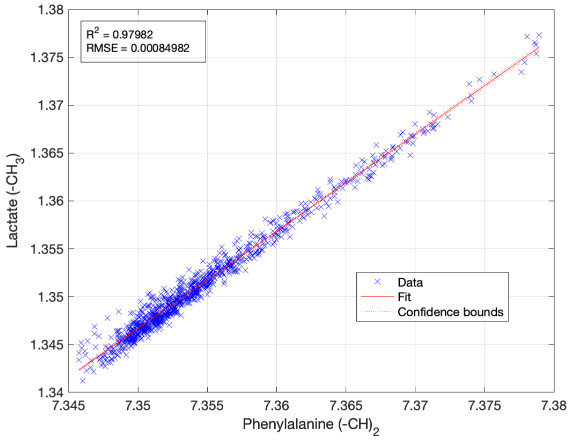

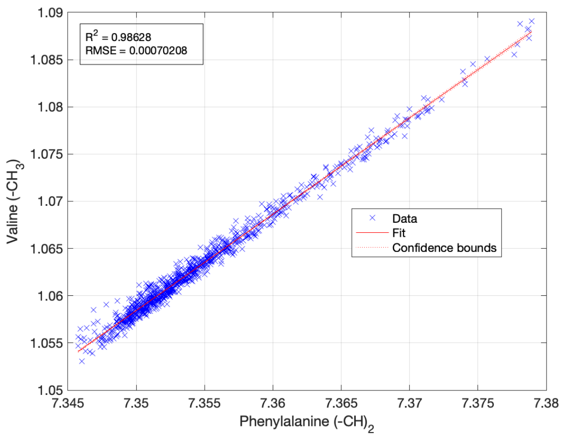

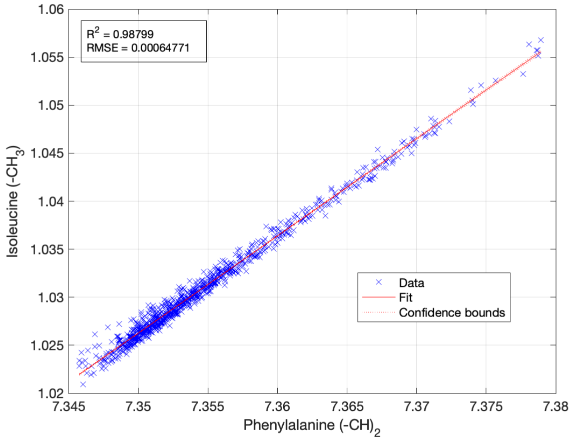

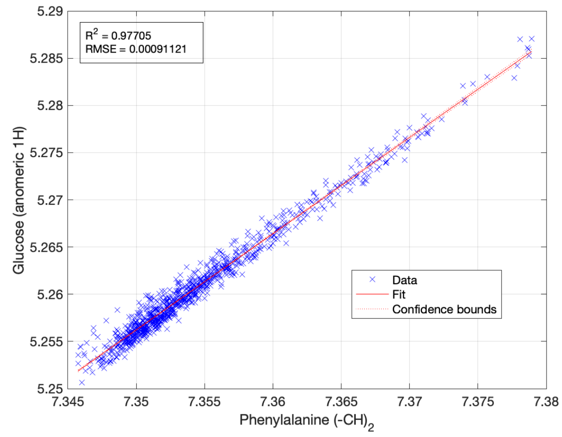

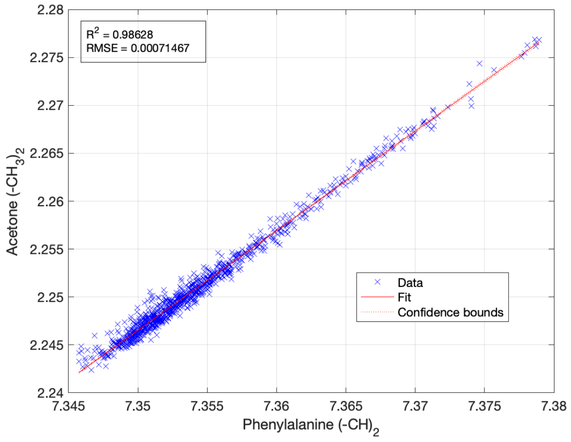

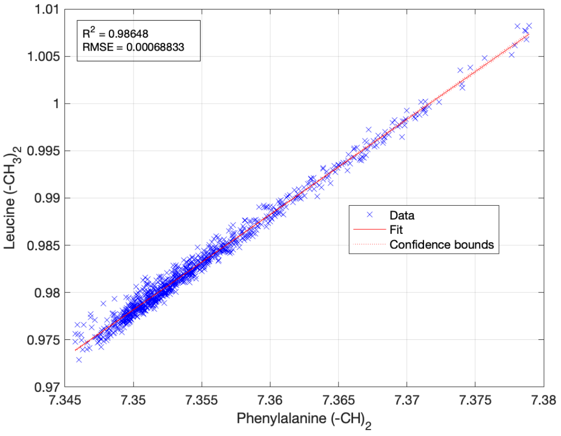
R^2 = 0.98648
RMSE = 0.00068833
Data
Fit
Confidence bounds
Leucine (-CH3)2
Phenylalanine (-CH)2

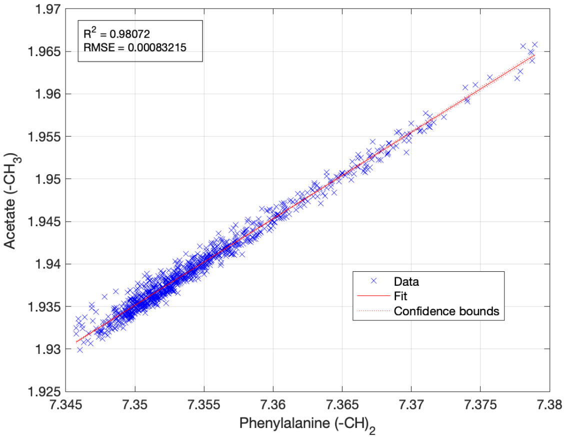
R^2 = 0.98072
RMSE = 0.00083215
Data
Fit
Confidence bounds
Acetate (-CH3)
Phenylalanine (-CH)2

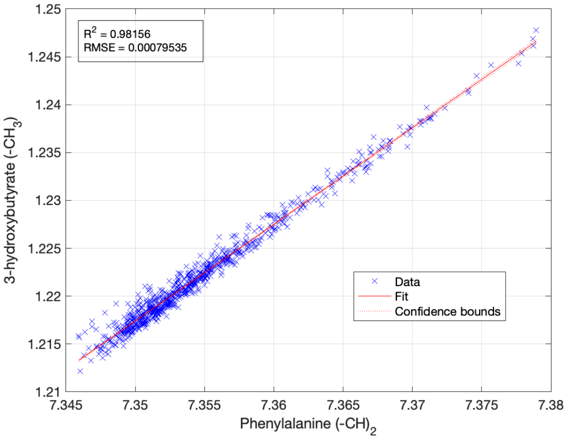
R^2 = 0.98156
RMSE = 0.00079535
Data
Fit
Confidence bounds
3-hydroxybutyrate (-CH3)
Phenylalanine (-CH)2

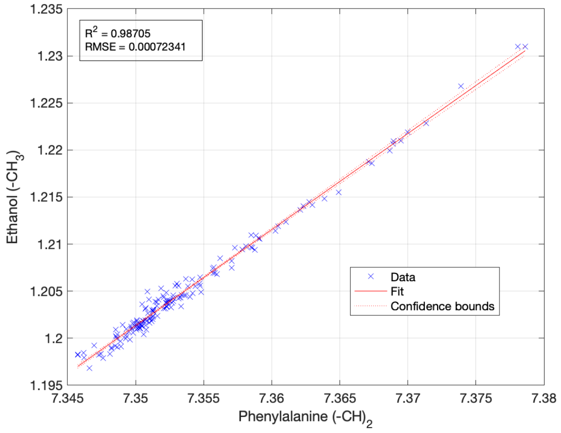
R^2 = 0.98705
RMSE = 0.00072341
Data
Fit
Confidence bounds
Ethanol (-CH3)
Phenylalanine (-CH)2

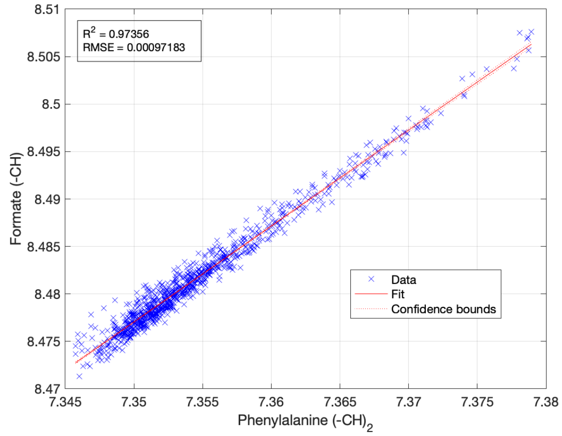
R^2 = 0.97356
RMSE = 0.00097183
Data
Fit
Confidence bounds
Formate (-CH)
Phenylalanine (-CH)2

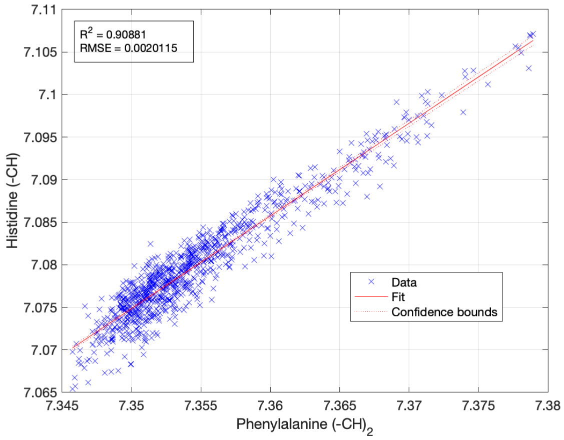
R^2 = 0.90881
RMSE = 0.0020115
Data
Fit
Confidence bounds
Histidine (-CH)
Phenylalanine (-CH)2

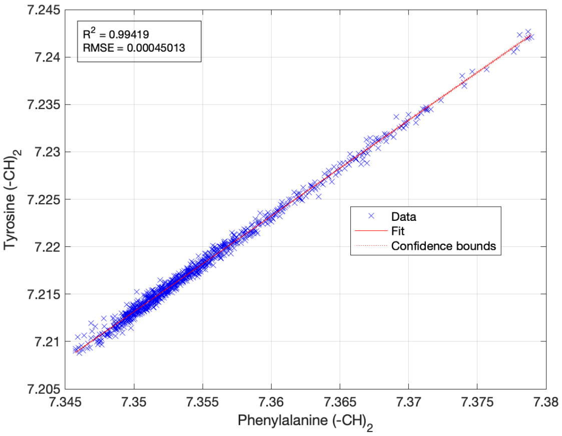
R^2 = 0.99419
RMSE = 0.00045013
Data
Fit
Confidence bounds
Tyrosine (-CH)2
Phenylalanine (-CH)2

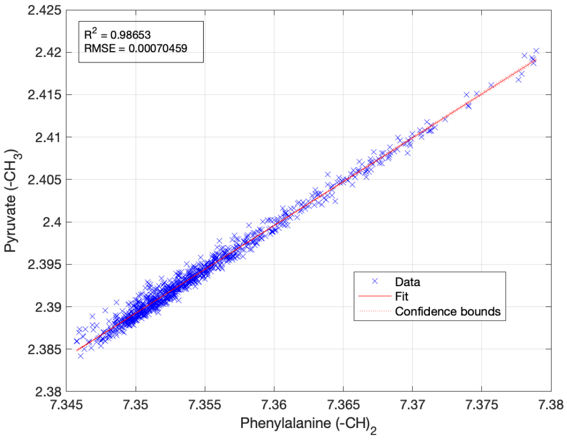
R^2 = 0.98653
RMSE = 0.00070459
Data
Fit
Confidence bounds
Pyruvate (-CH3)
Phenylalanine (-CH)2

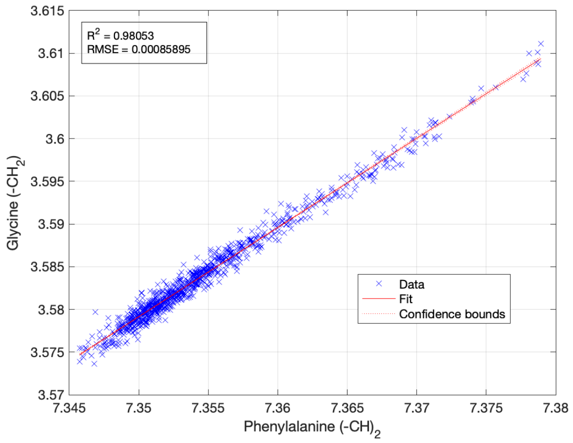

Figure S14. Scatter plots and fitted liner regression lines (y = a*x + b) for all spins systems with phenylalanine (-CH)$_2$ δ as the predictor(x). For each fitted model, the calculated R^2 and RMSE values are depicted.

Tyrosine as predictor (x)

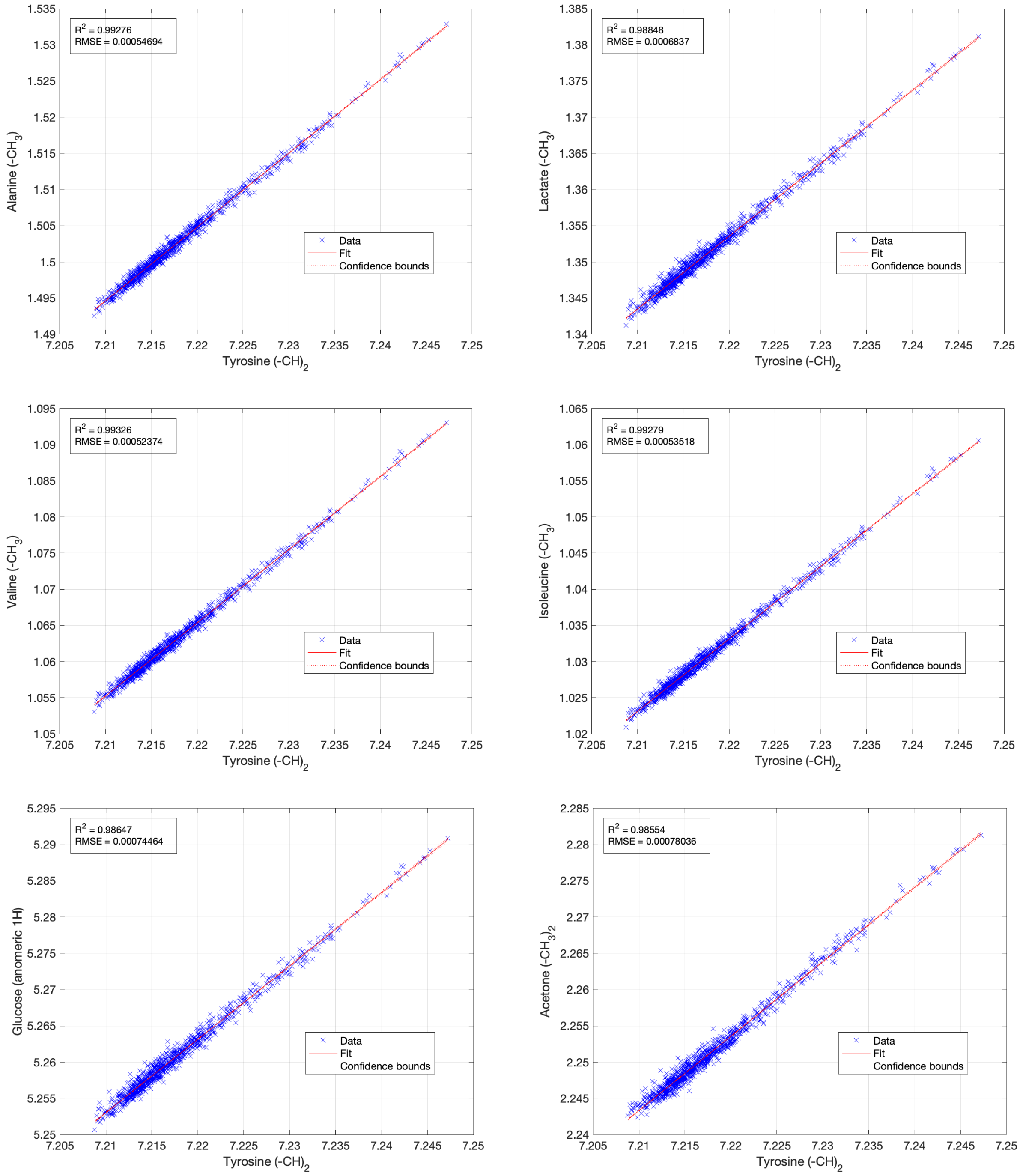

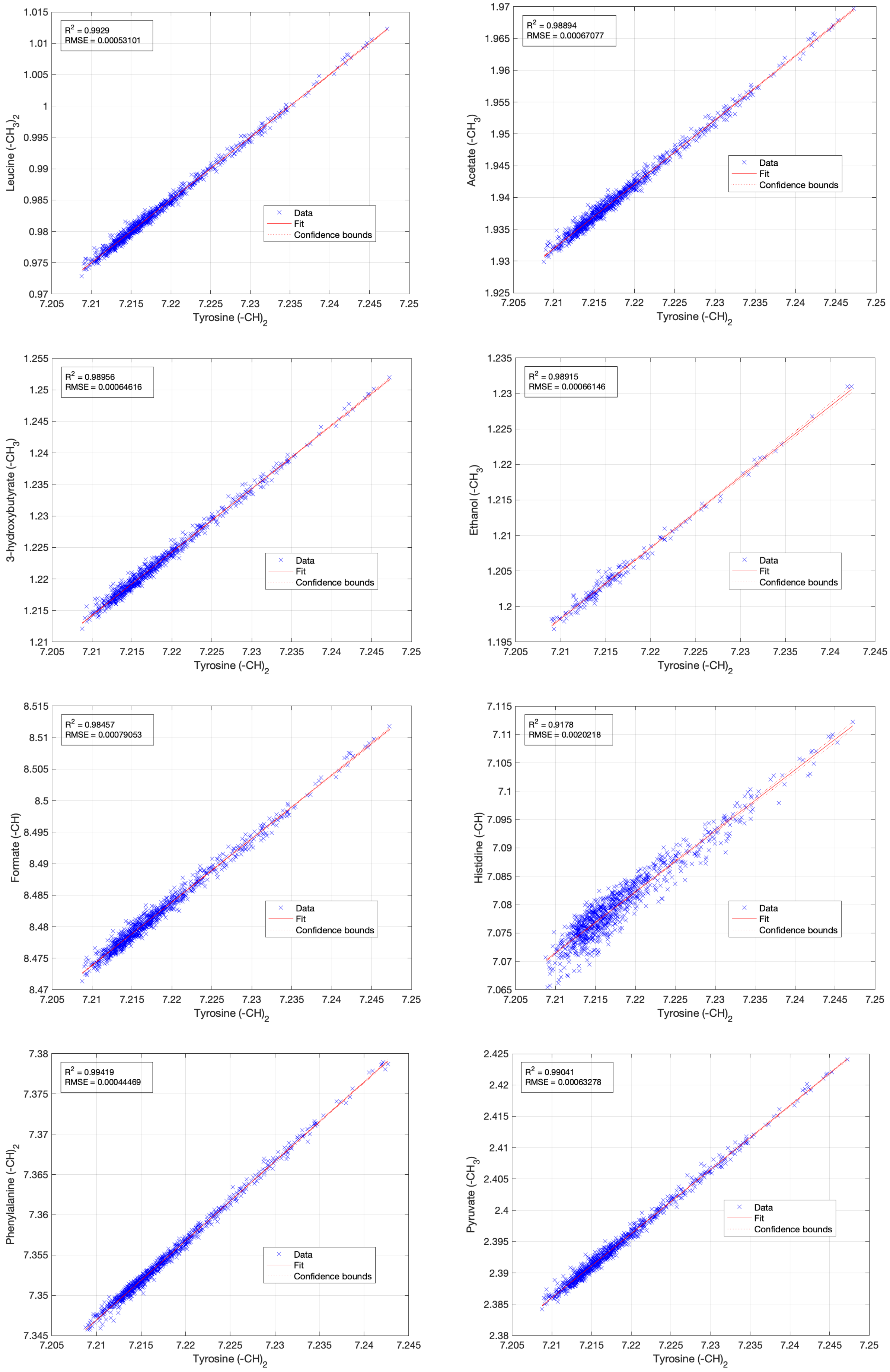

R2 = 0.9929
RMSE = 0.00053101
Leucine (-CH3)2
Tyrosine (-CH)2
Data
Fit
Confidence bounds
R2 = 0.98894
RMSE = 0.00067077
Acetate (-CH3)
Tyrosine (-CH)2
Data
Fit
Confidence bounds
R2 = 0.98956
RMSE = 0.00064616
3-hydroxybutyrate (-CH3)
Tyrosine (-CH)2
Data
Fit
Confidence bounds
R2 = 0.98915
RMSE = 0.00066146
Ethanol (-CH3)
Tyrosine (-CH)2
Data
Fit
Confidence bounds
R2 = 0.98457
RMSE = 0.00079053
Formate (-CH)
Tyrosine (-CH)2
Data
Fit
Confidence bounds
R2 = 0.9178
RMSE = 0.0020218
Histidine (-CH)
Tyrosine (-CH)2
Data
Fit
Confidence bounds
R2 = 0.99419
RMSE = 0.00044469
Phenylalanine (-CH)2
Tyrosine (-CH)2
Data
Fit
Confidence bounds
R2 = 0.99041
RMSE = 0.00063278
Pyruvate (-CH3)
Tyrosine (-CH)2
Data
Fit
Confidence bounds

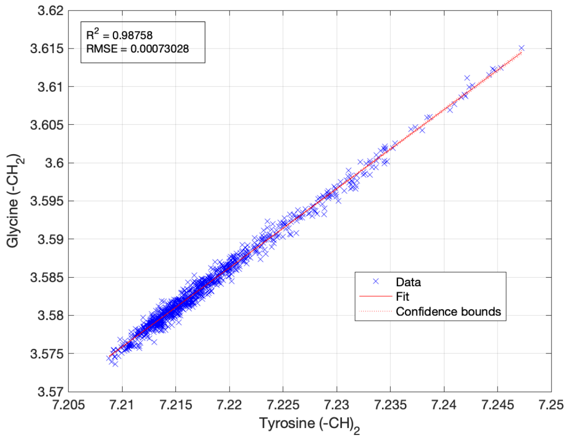

Figure S15. Scatter plots and fitted liner regression lines (y = a*x + b) for all spins systems with tyrosine $(\text{-CH})_2$ δ as the predictor(x). For each fitted model, the calculated R^2 and RMSE values are depicted.

Pyruvate as predictor (x)

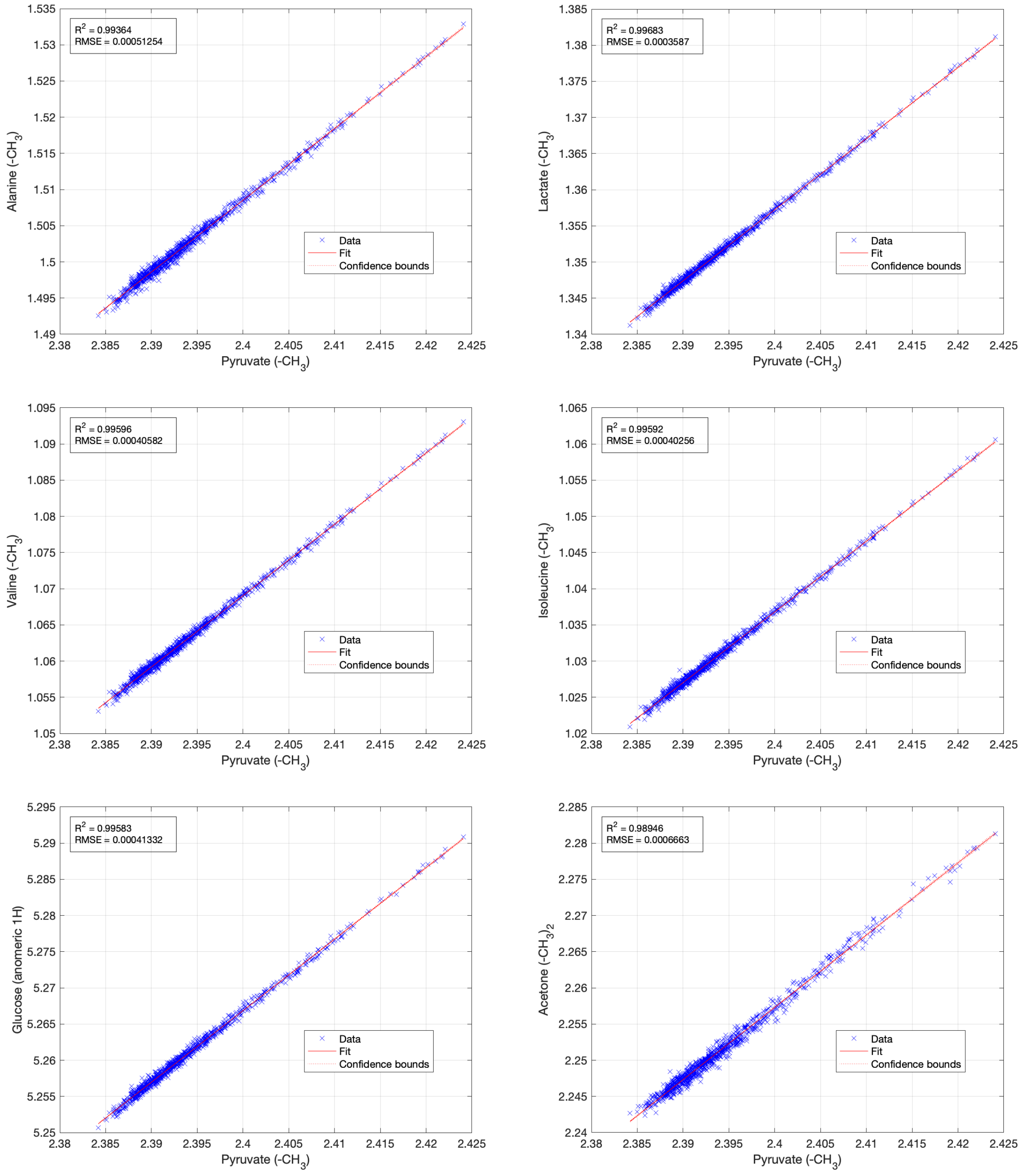

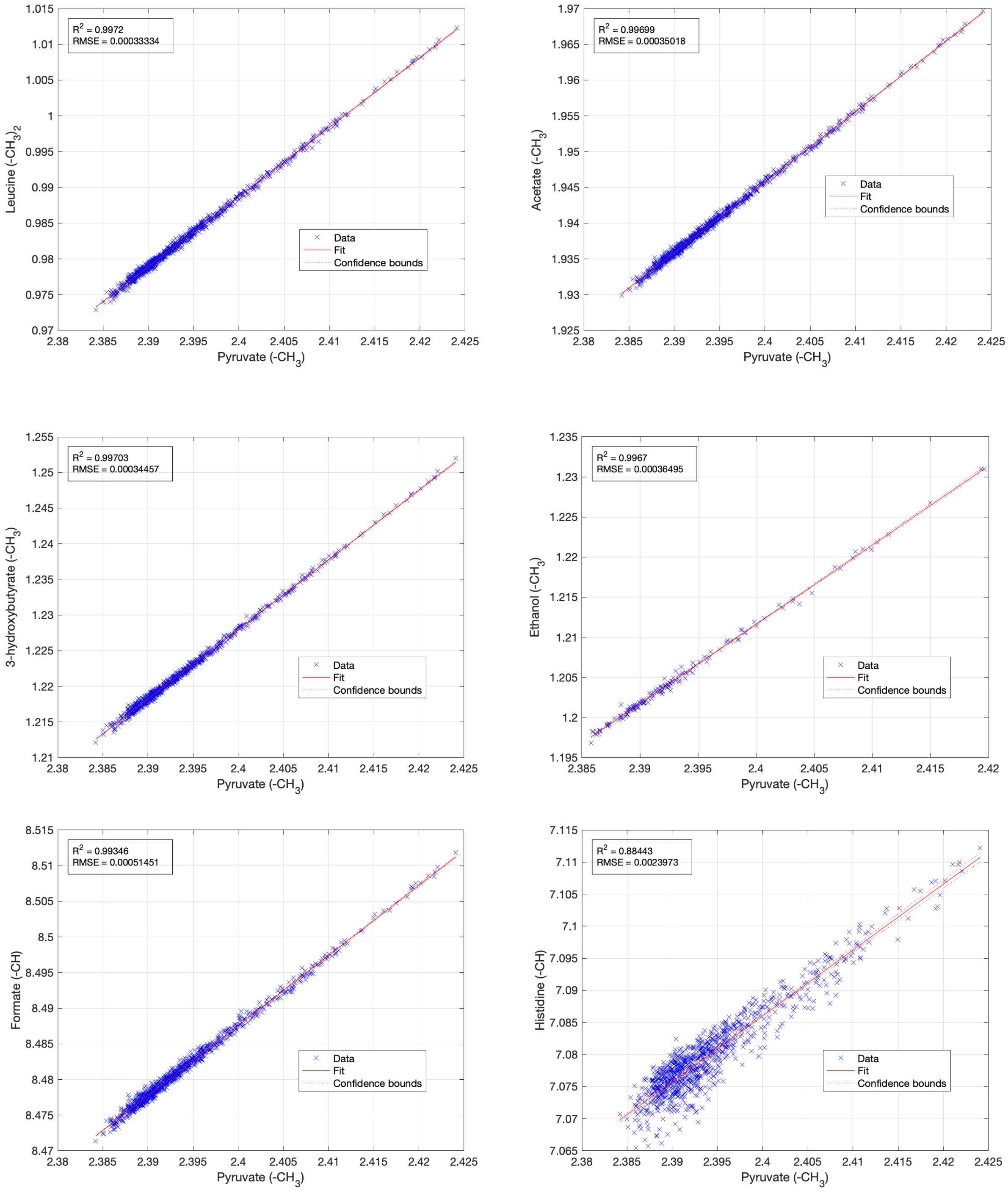
R2 = 0.9972
RMSE = 0.00033334
Leucine (-CH3)2
Pyruvate (-CH3)
Data
Fit
Confidence bounds
R2 = 0.99699
RMSE = 0.00035018
Acetate (-CH3)
R2 = 0.99703
RMSE = 0.00034457
3-hydroxybutyrate (-CH3)
R2 = 0.9967
RMSE = 0.00036495
Ethanol (-CH3)
R2 = 0.99346
RMSE = 0.00051451
Formate (-CH)
R2 = 0.88443
RMSE = 0.0023973
Histidine (-CH)

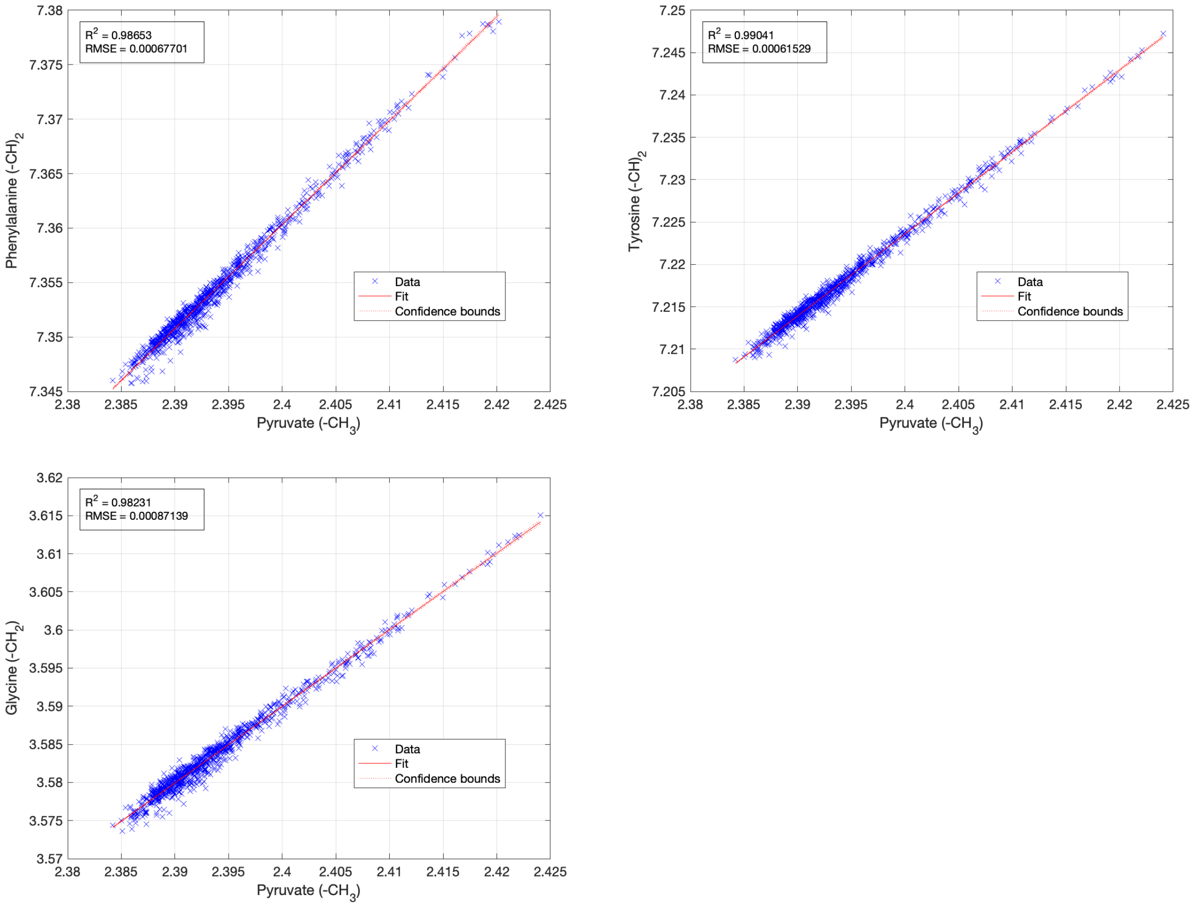

Figure S16. Scatter plots and fitted liner regression lines (y = a*x + b) for all spins systems with pyruvate $-CH_3$ δ as the predictor(x). For each fitted model, the calculated R^2 and RMSE values are depicted.

Glycine as predictor (x)

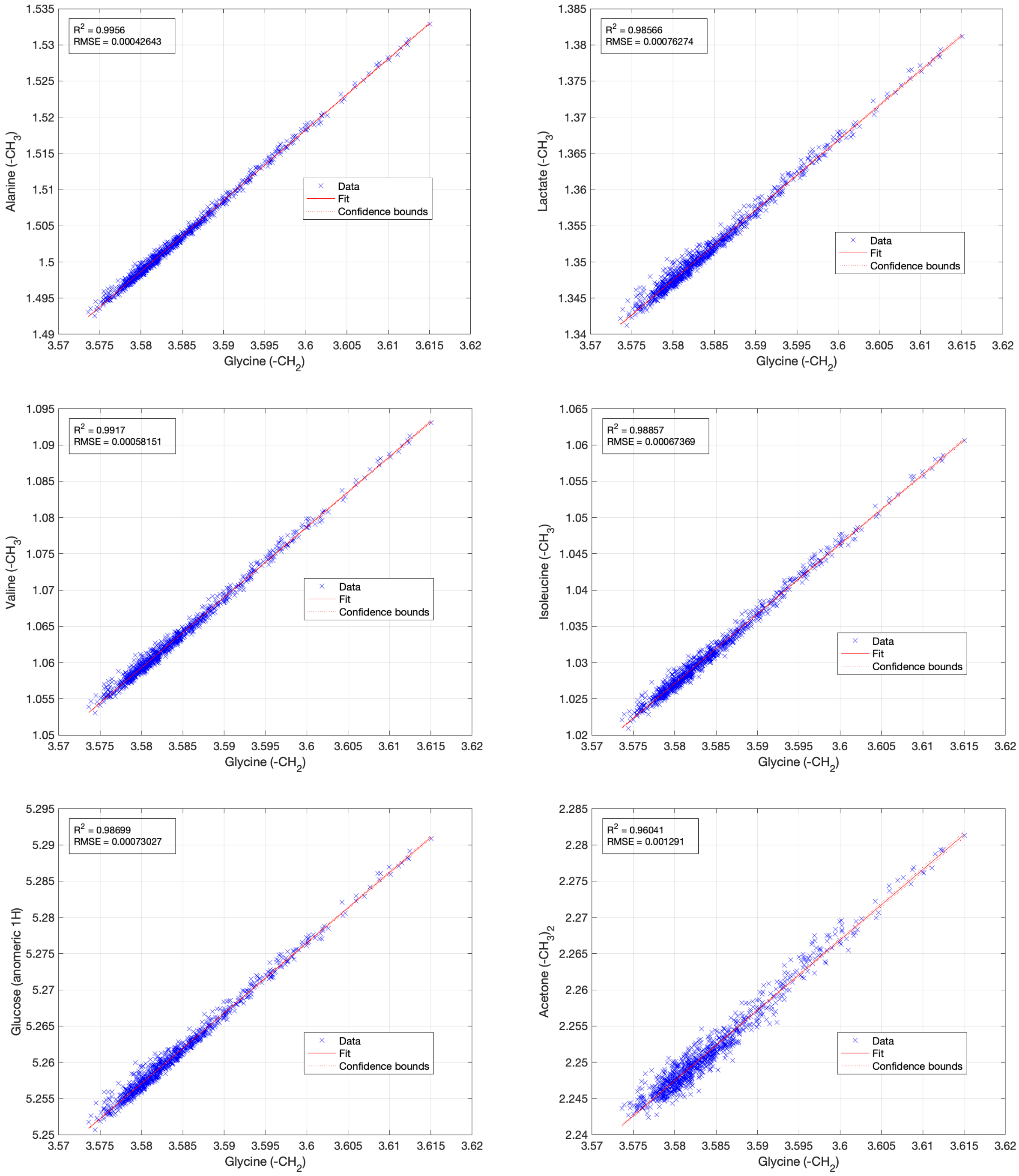

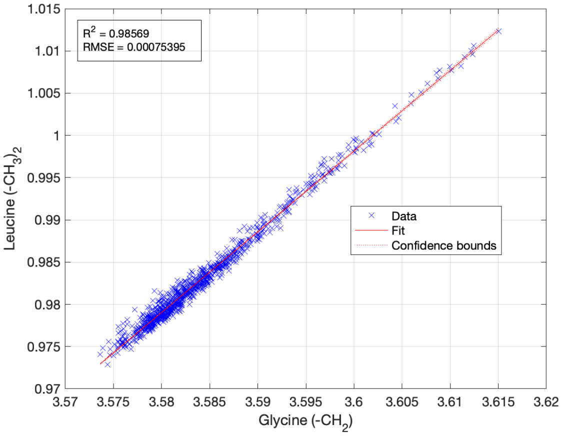
R^2 = 0.98569
RMSE = 0.00075395
Leucine $(-CH_3)_2$
Glycine $(-CH_2)$
Data
Fit
Confidence bounds

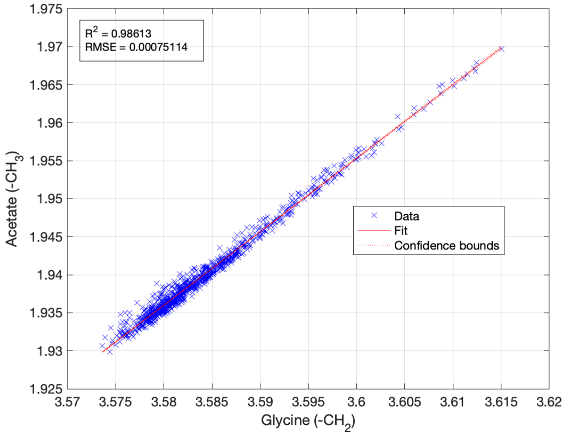
R^2 = 0.98613
RMSE = 0.00075114
Acetate $(-CH_3)$
Glycine $(-CH_2)$
Data
Fit
Confidence bounds

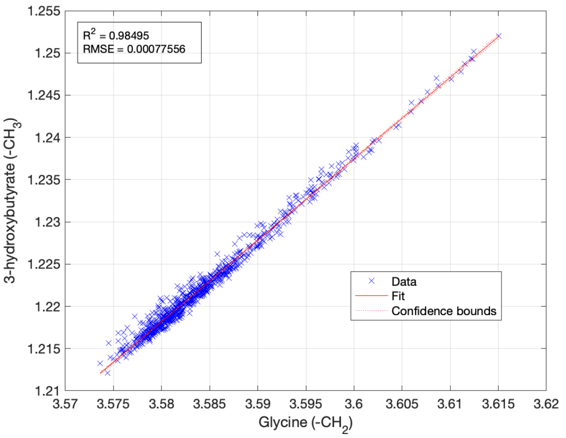
R^2 = 0.98495
RMSE = 0.00077556
3-hydroxybutyrate $(-CH_3)$
Glycine $(-CH_2)$
Data
Fit
Confidence bounds

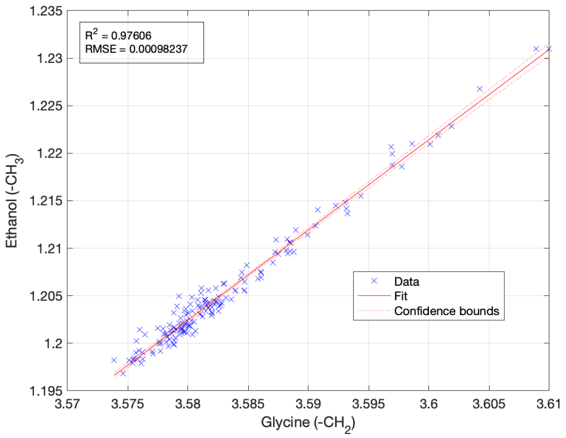
R^2 = 0.97606
RMSE = 0.00098237
Ethanol $(-CH_3)$
Glycine $(-CH_2)$
Data
Fit
Confidence bounds

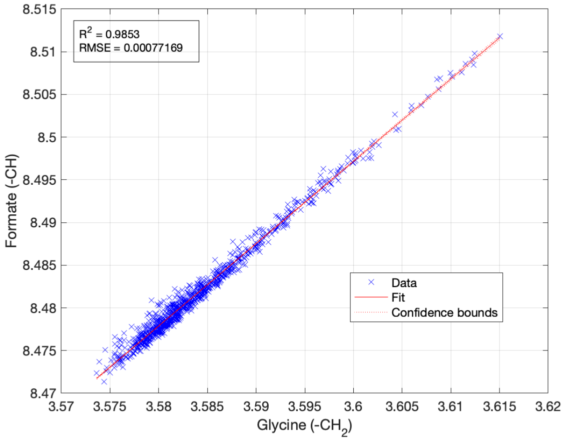
R^2 = 0.9853
RMSE = 0.00077169
Formate (-CH)
Glycine $(-CH_2)$
Data
Fit
Confidence bounds

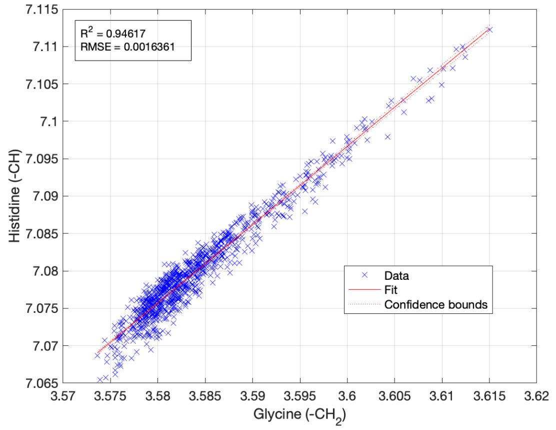
R^2 = 0.94617
RMSE = 0.0016361
Histidine (-CH)
Glycine $(-CH_2)$
Data
Fit
Confidence bounds

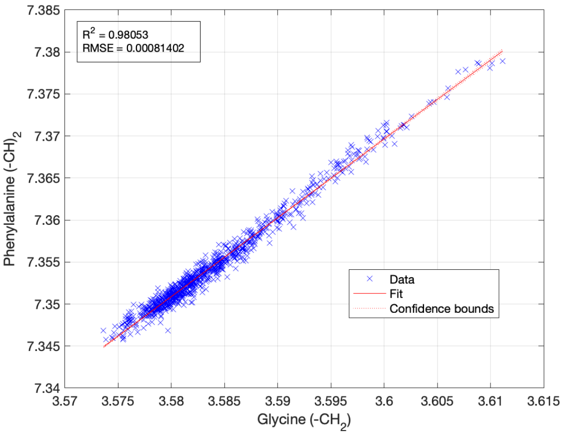
R^2 = 0.98053
RMSE = 0.00081402
Phenylalanine $(-CH)_2$
Glycine $(-CH_2)$
Data
Fit
Confidence bounds

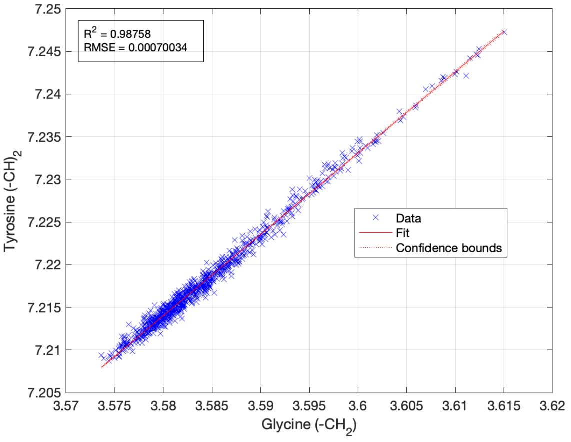
R^2 = 0.98758
RMSE = 0.00070034
Tyrosine $(-CH)_2$
Glycine $(-CH_2)$
Data
Fit
Confidence bounds

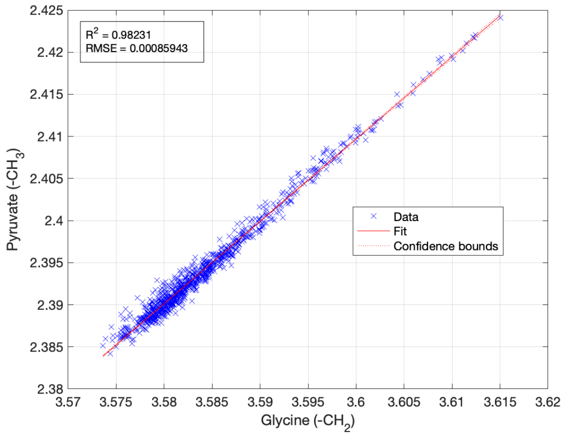

Figure S17. Scatter plots and fitted liner regression lines (y = a*x + b) for all spins systems with glycine -CH_2 δ as the predictor(x). For each fitted model, the calculated R^2 and RMSE values are depicted.

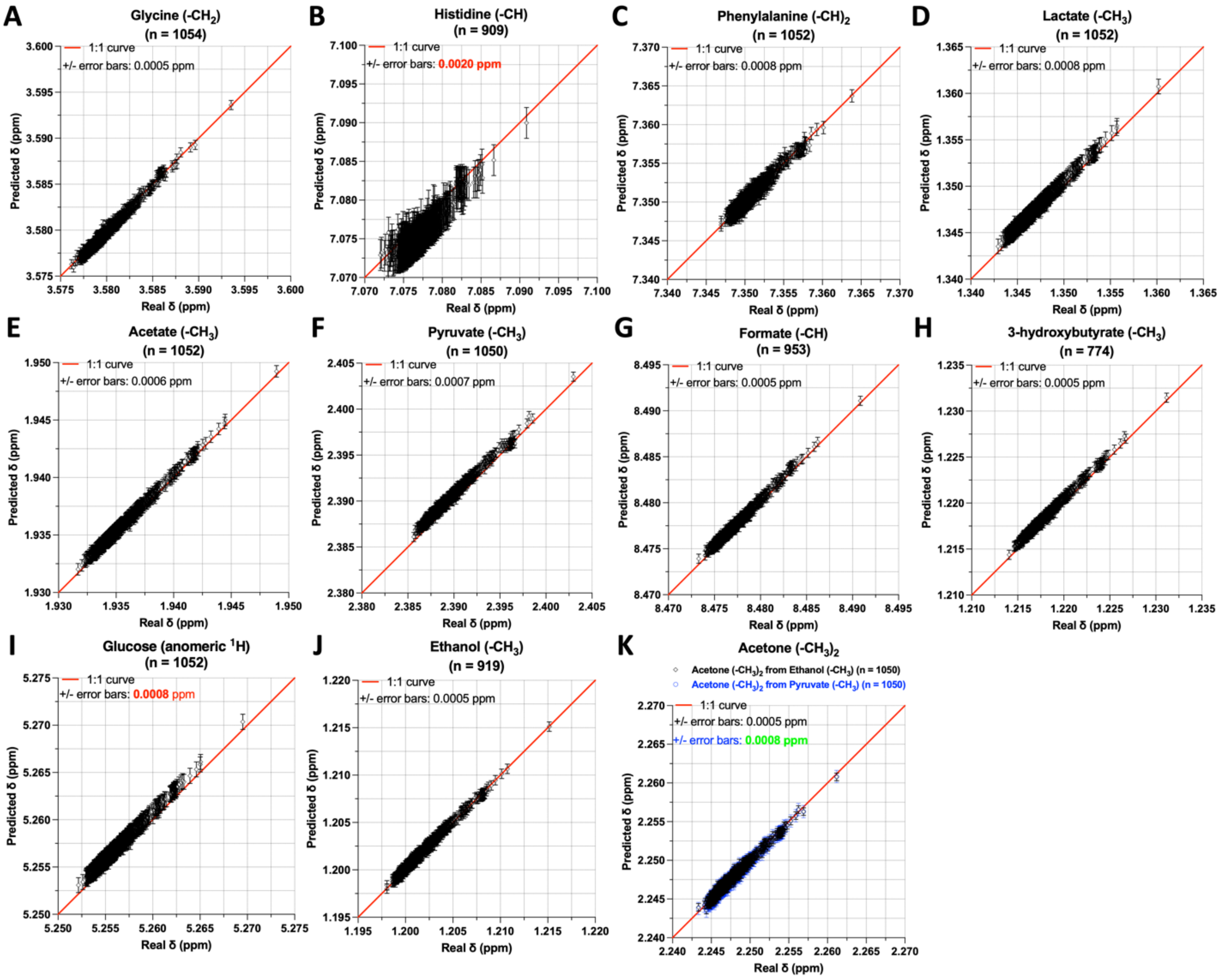

Figure S18. The performance of the final "map" (see Figure 6 of the main article) for the **automated** prediction of the studied spin systems tested in maximum 1052 plasma-EDTA spectra (i.e., the independent validation dataset). In particular, the real *δ* (i.e., assigned) values are plotted against the predicted values (in ppm). The ± error bars indicate the maximum calculated error of each model, based upon the validation datasets real *δ*. Red lines indicate the 1:1 line (i.e., perfect line). Results are for the following metabolites spin systems: (A) glycine, (B) histidine, (C) phenylalanine, (D) lactate, (E) acetate, (F) pyruvate, (G) formate, (H) 3-hydroxybutyrate, (I) glucose, (J) ethanol and (K) acetone.

Examples of chemical shifts predictions on one validation spectrum

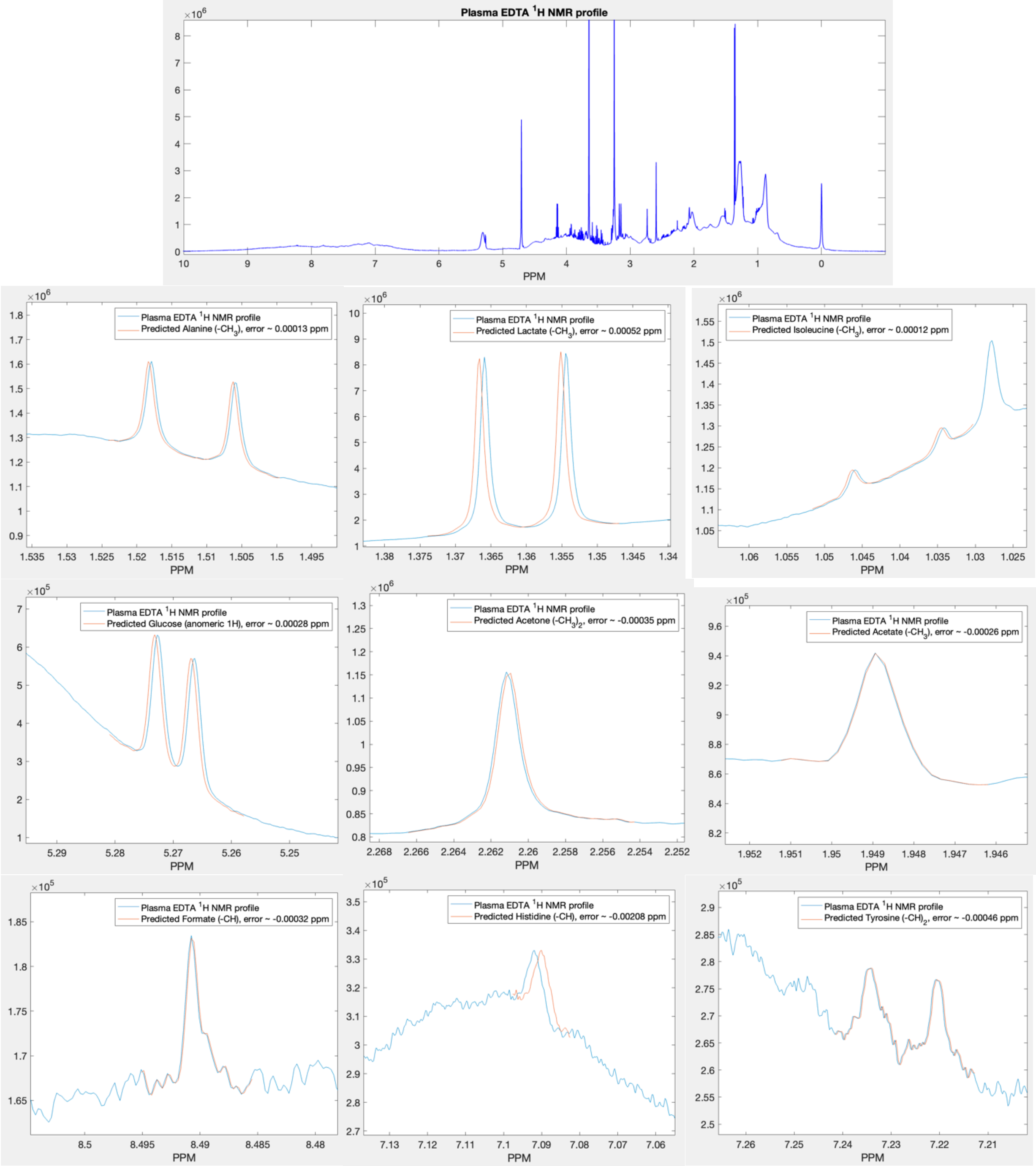

Figure S19. Examples of various predicted ^{1}H NMR chemical shifts (red line) plotted versus the real NMR profile. Plotting was achieved by calibrating the spectrum at the predicted ppm value of each spin system.

Linear regression functions from the best models and corresponding statistics

Valine as predictor (x) of Leucine (y)

Estimated Coefficients:

	Estimate	SE	tStat	pValue
(Intercept)	−0.066609	0.0011134	−59.822	2.0751e−322
x1	0.98711	0.0010471	942.68	0

Valine as predictor (x) of Isoleucine (y)

Estimated Coefficients:

	Estimate	SE	tStat	pValue
(Intercept)	−0.022576	0.0011381	−19.838	2.5394e−73
x1	0.99095	0.0010703	925.87	0

Valine as predictor (x) of Alanine (y)

Estimated Coefficients:

	Estimate	SE	tStat	pValue
(Intercept)	0.43197	0.00098692	437.7	0
x1	1.007	0.00092815	1085	0

Valine as predictor (x) of Tyrosine (y)

Estimated Coefficients:

	Estimate	SE	tStat	pValue
(Intercept)	6.1746	0.0028054	2200.9	0
x1	0.98126	0.0026384	371.92	0

Alanine as predictor (x) of Glycine (y)

Estimated Coefficients:

	Estimate	SE	tStat	pValue
(Intercept)	2.0561	0.0033167	619.91	0
x1	1.0169	0.0022071	460.73	0

Glycine as predictor (x) of Histidine (y)

Estimated Coefficients:

	Estimate	SE	tStat	pValue
(Intercept)	3.3276	0.029223	113.87	0
x1	1.047	0.0081534	128.41	0

Tyrosine as predictor (x) of Phenylalanine (y)

Estimated Coefficients:

	Estimate	SE	tStat	pValue
(Intercept)	0.2447	0.017835	13.72	3.7976e−39
x1	0.98505	0.0024709	398.66	0

Lactate as predictor (x) of Acetate (y)

Estimated Coefficients:

	Estimate	SE	tStat	pValue
(Intercept)	0.58699	0.0008365	701.72	0
x1	1.0011	0.00061893	1617.5	0

Lactate as predictor (x) of Pyruvate (y)

Estimated Coefficients:

	Estimate	SE	tStat	pValue
(Intercept)	1.0254	0.0025206	406.81	0
x1	1.0128	0.001865	543.04	0

Lactate as predictor (x) of Formate (y)

Estimated Coefficients:

	Estimate	SE	tStat	pValue
(Intercept)	7.1326	0.0017005	4194.5	0
x1	0.99834	0.0012582	793.47	0

Lactate as predictor (x) of 3-hydroxybutyrate (y)

Estimated Coefficients:

	Estimate	SE	tStat	pValue
(Intercept)	−0.11993	0.0014843	−80.802	0
x1	0.99313	0.0010981	904.42	0

Lactate as predictor (x) of Glucose (y)

Estimated Coefficients:

	Estimate	SE	tStat	pValue
(Intercept)	3.9033	0.0011386	3428.1	0
x1	1.0046	0.00084247	1192.5	0

Leucine as predictor (x) of Ethanol (y)

Estimated Coefficients:

	Estimate	SE	tStat	pValue
(Intercept)	0.21972	0.0044607	49.257	1.4234e−96
x1	1.0033	0.0045416	220.91	1.2513e−195

Ethanol as predictor (x) of Acetone (y)

Estimated Coefficients:

	Estimate	SE	tStat	pValue
(Intercept)	1.0378	0.0093971	110.44	2.8345e−149
x1	1.0065	0.0077976	129.08	1.1615e−159

Pyruvate as predictor (x) of Acetone (y)

Estimated Coefficients:

	Estimate	SE	tStat	pValue
(Intercept)	−0.13993	0.0080606	−17.36	9.4102e−59
x1	0.99885	0.0033668	296.68	0

Lactate as predictor (x) of Leucine (y)

Estimated Coefficients:

	Estimate	SE	tStat	pValue
(Intercept)	0.35862	0.0011634	308.27	0
x1	1.0101	0.0011835	853.49	0
